# Impact of windbreak design on microclimate in hot regions during cold waves: Numerical investigation

**DOI:** 10.1007/s00484-024-02668-8

**Published:** 2024-05-06

**Authors:** Mohamed E. Abdalazeem, Hamdy Hassan, Takashi Asawa, Hatem Mahmoud

**Affiliations:** 1 Department of Environmental Engineering, Egypt-Japan University of Science and Technology E-JUST, New Borg El-Arab City, Alexandria, 21934 Egypt; 2 Department of Architecture Engineering, Engineering Faculty, Assiut University, Assuit, Egypt; 3 Department of Energy Resources, Egypt-Japan University of Science and Technology E-JUST, New Borg El-Arab City, Alexandria, 21934 Egypt; 4 Department of Mechanical Engineering, Engineering Faculty, Assiut University, Assuit, Egypt; 5 Tokyo Institute of Technology, Yokohama, 226-8502 Japan; 6 Department of Architecture Engineering, Engineering Faculty, Aswan University, Aswan, 81542 Egypt

**Keywords:** Windbreak parameters design, Cold stress mitigation, Hot temperature, Urban microclimate, Thermal comfort, Envi-Met simulation

## Abstract

Winter cold wave adaptation strategies in hot climates due to climate change didn’t receive the deserved attention from previous studies. Therefore, this study comprehensively investigates the impact of various windbreak parameters on mitigating winter cold stress in hot steppe-arid climate. A microclimate model for a residential campus was built and validated through on-site measurement on a typical winter day to assess thirty-two scenarios for tree characteristics and spatial configuration windbreak parameters based on PET, wind speed, and Air Temperature (AT). Moreover, four configurations, that had best results on mitigating cold stress in winter, were tested during typical summer conditions to couple the assessment of cold and hot seasons. Additionally, environmental analysis for all scenarios was conducted. The results revealed that the most effective parameters for mitigating cold stress are tree distribution, Leaf Area Density (LAD), row number, spacing, and shape. Double rows of high LAD and medium height trees with small spacing yielded the best cold stress mitigation effect. Furthermore, the windbreak reduced the cold stress in the morning and night by 19.31% and 18.06%, respectively. It reduced AT and wind speed at night by 0.79 °C and 2.56 m/s, respectively. During summer, very hot PET area was reduced by 21.79% and 19.5% at 12:00 and 15:00, respectively.

**Supplementary Information:**

The online version contains supplementary material available at 10.1007/s00484-024-02668-8.

## Introduction

Extremely cold waves have increased during winter in recent years even in hot climates due to global climate change (Li et al. 2022) which increasing residential buildings’ energy consumption (Ko 2018), maximizing the number of deaths, deteriorating crop yield, and reducing outdoor activities in urban areas (Smith et al. 2021). Unfortunately, many urban areas aren’t invulnerable to cold waves in winter to mitigate cold stress in outdoor spaces (Li et al. 2022) due to the massive focus from previous studies on protecting urban areas from extremely hot conditions (Abdalazeem et al. 2022), (Mahmoud & Ghanem 2019), (Mahmoud & Ragab 2021). Despite Egypt's hot arid climate, Egypt experienced in January and February 2022 the coldest winter in the last 10 years, a problem that plagues the country's north every winter. In these cold waves, the temperature dropped by 7–8°C to reach less than 5°C which is lower than the average level during this time of the year in Egypt (English.ahram.org.eg 2022). These waves combined with high windspeed, heavy rain with floods, and snowfall for the first time (El-Geziry 2021) which led to massive health risks and an increasing number of deaths (Li et al. 2022). Therefore, various studies used trees belts as a windbreak (WB) to protect outdoor spaces and building envelopes from severe cold conditions, enhance the climate adaptation strategies of urban areas (Girona et al. 2019), reduce heating energy consumption (Ko 2018) and reduce air velocity (Baker et al. 2021). However, tree cover mainly used to mitigate the climate change effect and improve various outdoor thermal comfort (OTC) indexes during the summer months. (Zhao & Fong 2017).

Previous windbreak studies could be divided into several investigation points related to the main aim, climate regions, windbreak factors, investigated seasons and performance parameters, as shown in Table A1. Firstly, the main focus of previous studies to use the vegetated windbreak to protect open areas from high winds (Iwasaki et al. 2021), (Wu et al. 2022), improving air quality by reducing dust concentration (Taleb & Kayed 2021), (Chang et al. 2019), (Moskovaya et al. 2017), improving animal thermal comfort (He et al. 2017) and protect the crops yield in farms (Iwasaki et al. 2021). Secondly, For the climate regions, most windbreak studies mainly focused on reducing wind speed in farms and open areas in cold climates (Chang et al. 2019), (Yukhnovskyi et al. 2021), as shown in Table A1. For windbreak factors, there are two main categories: internal factors (Tree type, shape, height, shading coverage ratio, and canopy diameter) and external factors (number of trees, tree spacing, number of tree rows and the position of tree belt). For internal parameters, (Jian et al. 2018) found that cylinder big canopy trees were more effective in reducing wind speed in winter compared to conical and quadrilateral pyramid shapes. Also, (Afshar et al. 2018) used deciduous trees and grass in the middle of the urban park to improve OTC in winter in cold climate. However, (Li & Dong 2021) found that the mixed windbreak configuration between tall trees and small shrubs is most effective in reducing wind speed than the single row of trees. For LAD, the moderately dense-foliage tree achieved the best outdoor thermal conditions and maximum wind speed reduction during a cold storm compared to low LAD in a humid subtropical climate (Li et al. 2022). Also, (Yukhnovskyi et al. 2021) stated that moderate LAD of three crowns improved the aerodynamic of wind in farms more than the high dense windbreak in cold climate. Regarding external parameters, small tree spacing reduced wind speed and dust emissions (Chang et al. 2021), (Jian et al. 2018). Furthermore, (Bitog et al. 2012) found that reducing the tree spacing to 0.5 m is more effective in reducing air velocity compared to 0.75 m and 1.0 m by 6% and 15%, respectively through using wind tunnel experiments. Moreover, increasing the number of tree rows is more effective in reducing wind speed and air pollution (Cornelis et al. 2000), especially if these rows contain alternating trees (Bitog et al. 2012). Concerning wind direction, the best windbreak position is to be perpendicular to the wind direction to mitigate the wind speed (Taleb & Kayed 2021), (Tamang et al. 2010), (Baker et al. 2021). This effect of windbreak on reducing wind speed could reach a distance up to 15 times the windbreak height (Yukhnovskyi et al. 2021), (Bitog et al. 2012), 20 times (Baker et al. 2021), 25 times (Ma et al. 2019) or 31 times (Tamang et al. 2010). Finally, different performance parameters are used to investigate the effect of windbreak on microclimate conditions such as (AT), RH (Li et al. 2022), PM (Taleb & Kayed 2021) and land surface temperature (Iwasaki et al. 2021), (Wang et al. 2019). However, the main focus was on reducing wind speed (Wu et al. 2022), (Ma et al. 2019).

**Table 1:** Different windbreak scenarios

Sc	Tree type	Space	Position	Rows	Sc	Tree type	Space	Position	Rows
Low LAD (LAD = 2)									
S1	Ficus	2m	West & south	Single	S9	Conocarpus	2m	West & south	Single
S2		4m			S10		4m		
S3		6m			S11		6m		
S4		2m		Double	S12		2m		Double
S5		4m			S13		4m		
S6		6m			S14		6m		
S7		2m	Border &inside		S15		2m	Border &inside	
S8	Ficus (In)& Cono.(Out)				S16	Cono.(In)& Ficus (Out)			
High LAD (LAD = 5)									
S17	Ficus	2m	West & south	Single	S25	Conocarpus	2m	West & south	Single
S18		4m			S26		4m		
S19		6m			S27		6m		
S20		2m		Double	S28		2m		Double
S21		4m			S29		4m		
S22		6m			S30		6m		
S23		2m	Borders& inside		S31		2m	Borders& inside	
S24	Ficus (In)& Cono.(Out)				S32	Cono. (In)& Ficus (Out)			

Based on the authors’ survey, several points need to be investigated. Firstly, the cold stress is not placed similar significance as the heat stress inside urban areas in green cover studies, particularly in hot arid climates. Therefore, few studies investigate the feasibility of the windbreak in mitigating cold stress in hot climates. Secondly, to the author’s best knowledge, no study introduced a comprehensive investigation of different tree parameters and spatial configuration parameters on mitigating cold stress in winter. Also, most of the windbreak studies focused on reducing wind speed as a single parameter to improve thermal conditions in winter ignoring other parameters and a comprehensive thermal comfort index (Id et al. 2020), as shown in Table A1. Finally, all previous windbreak studies didn’t test the impact of their optimal scenarios in winter, during the different seasons of the year, especially under hot summer conditions to couple the assessment of cold and hot seasons. To bridge these gaps, this study aimed to comprehensively investigate the effect of different windbreak parameters (tree characteristics and spatial configuration) on outdoor thermal conditions: (PET, (AT) at the pedestrian level and windspeed) on cold days in hot climate. This study investigated the effect of LAD, tree height, tree spacing, number of tree rows and the position of tree belt on mitigating the cold stress in hot climate. To achieve this aim, A 3D ENVI-met simulation model for a low-building-density residential campus was built and validated through field measurements on a cold typical winter day in Egypt. Thirty-two scenarios investigated different windbreak parameters were simulated to figure out the best windbreak configuration in winter. The best configurations (four scenarios) were tested under typical hot summer conditions to couple the assessment of cold and hot seasons. Finally, the environmental and economic analyses were carried out for different scenarios.

## Methods and materials

The study is divided into six major sections, as shown in Figure B 1. Firstly, the theoretical part specifies the potential research gaps and the main aim of the study. Secondly, the site measurements for microclimate conditions were conducted inside a residential campus in Egypt during a winter day. After that, a holistic three-dimensional microclimate model of the residential campus was built and validated through field measurement. Furthermore, a survey of the most common tree species used as a windbreak was made to select the most widespread trees in the city of the case study. Three-dimensional models for these trees are built to be used in the simulation process. In the fifth step, thirty-two different scenarios were simulated to investigate the effect of different windbreak factors on improving wind speed reduction, PET and (AT) at the pedestrian level during different times (early morning, afternoon, and night). Finally, the best configurations of the windbreak under cold conditions were investigated in the summer conditions to ensure their validity in both cold and hot seasons.

### Study area

This study was conducted in New Borg El-Arab City (NBAC) in the north of Egypt close to the Mediterranean Sea (Lat.:30°52′05" N and Long.: 29°34′54"E) which has a hot steppe arid climate (BSh) based on Köppen climate classification (Abdalazeem et al. 2024). In winter, the city has rainy very cold weather from November to February with minimum (AT) can be ranged between 8–10 °C at night (www.meteoblue.com^2022^). These cold temperatures continued for three-quarters of the winter months. This low temperature is considered unique and extremely cold for the residents of this city (Lai et al. 2021). Also, there is a high wind speed that could reach 21.6 km/h (6m/s) with low building densities and heights which maximizes the effect of cold waves (www.freemeteo.com^2022^). The dominant wind direction in NBAC ranged between northwest, west, and southwest directions, as shown in Figure B 2.

### Urban configuration of case study

NBAC and all new Egyptian cities have the same urban features according to the Egyptian building code (Abdelazzzim et al. 2018). A residential campus for Egypt Japan University in NBAC was selected as a case study due to several reasons. Firstly, the urban density of the campus is very low (25%) with the maximum buildings’ height (16.5m) like other campuses in the city. Secondly, the exterior finishing of facades has the same color and materials which makes the reflected radiation from the facades to the outdoor spaces the same and reduces the unexpected effect on the microclimate conditions, as shown in Figure B 3. Thirdly, the campus’s occupancy rate is near full percentage. Finally, the campus suffers from the decline of green cover, and 56.3% of the campus was covered by light grey concrete pavement, as shown in Table A 2.

### Field measurements

According to the weather history of NBAC, the week between 6th-12th January has the most typical cold winter weather (https://climate.onebuilding.org^2022^), (Abdalazeem et al. 2023). Therefore, Field measurement was conducted for 30 h from 00.00 on 11th to 6.00 on 12th January on the campus to be used for the simulation validation. The maximum and minimum measured AT on this day at the reference point were 23.3°C and 8.8°C, which is close to the average recorded history on January 19°C and 9°C. The study specified an additional three measurement points which were selected on the outdoor spaces in the campus with different orientations and H/W ratios, as shown in Table A 3 and Figure B 4. To validate the model, The measurements of the reference point were used because it is away from the buildings and faces the coming wind and continuous solar radiation to avoid any obstacles that can negatively affect the measurements. The measurements in summer were carried out on a typical summer day (8th August 2022) at the same Reference Point because this day is located inside the hottest week of the year according to the file weather history of NBAC. These measurements were used as simulation input to investigate the performance of the best scenarios in summer. Based on the previous studies’ recommendations, the field measurements for microclimate conditions were conducted at a level almost like pedestrian levels such as 1.5 m (He et al. 2017), (Zheng et al. 2016) up to 2.0 m (Galal et al. 2020), (Baker et al. 2021), (Tamang et al. 2010). However, these measurements in this study were monitored on the 1.8 m height from the ground because it is exactly the human height, and the most effective height to express human thermal comfort (Mahmoud et al. 2021).

Outdoor AT, Relative Humidity (RH), the concentration of CO_2_ level (ppm), Mean Radiant Temperature (MRT) and wind speed were measured using different measurement tools, as shown in Table A 4. The maximum and minimum (AT) during the daytime and night at different points were between 20°C- 23.3°C and 8.8°C- 9.5°C, respectively, as shown in Figure B 5.a,b. The maximum and minimum RH at different points ranged between 58.2%- 59.9% and 16.9%- 22.2%, respectively, as shown in Figure B 5.a,b. Also, the maximum and average wind speed in different measurement points at 1.5 m height reached up to 12.5 m/s and 3.6 m/s coming from the southwest (160° from the north), as shown in Figure B 5.c. For the CO_2_ concentration, the level ranged between 389–449 ppm at different points, as shown in Figure B 5.d. In summer, the outdoor AT and RH ranged between 24.3°C- 40°C and 32.5%- 75.5%, respectively, as shown in Figure B 6. While the average wind speed recorded in summer was 1.5 m/s.

### Simulation model

The ENVI-met is the most used software to simulate microclimate conditions compared to other software: EBM-based models, TRNSYS, STEVE and Urban Weather Generator (UMG) (Liu et al. 2021). ENVI-met gives comprehensive calculations for different thermal comfort indices such as PET with high-resolution output for complex geometries. Therefore, numerous studies used ENVI-met to predict the thermal performance of urban trees on microclimate conditions in summer (Salman & Saleem 2021), and winter (X. Li et al. 2022). Additionally, ENVI-met V5.0.3 increased the ability to create 3D trees models with different and complex geometries, which improved the accuracy of the simulation process (Ouyang et al. 2023). Therefore, the residential campus model was built using ENVI-met V5.0.3 software, as shown in Figure B 7. Based on previous studies, the most recommended configuration was followed during building the model to ensure its feasibility. The model size was 140 × 110 × 30 grids while the horizontal grid cell size was 2 × 2m. Seven additional grids were around the campus model to minimize the turbulence. The simulation process used simple forcing simulation to consider the AT and RH for each hour at the reference point. Table A 6 showed the main materials that were used in this model and their settings. Also, Table A 5 clarifies the file's main inputs such as the location, grid size, etc.… However, ENVI-met simulation input required the wind speed at 10 m height. As a result, the calculated wind speed at 10 m height from the ground is 3.67 m/s according to Eq. 1 (www.calculatoratoz.com^2022^).

$$\mathrm{Wind Speed at z height}=\left(\mathrm{Friction Velocity}\div {\text{Von}} \mathrm{K\acute{a} }\mathrm{rm\acute{a} }{\text{n}} {\text{Constant}}\right)\times {\text{ln}}\left(\mathrm{Height z above the Surface }\div \mathrm{Roughness Height of the Surface}\right)$$ 1.

### Model validation

Previous studies compared different simulated thermal parameters with measured data at specific points which was mainly AT (X. Li et al. 2022), or RH with AT (Galal et al. 2020). However, this ENVI-met model was validated through AT, RH and MRT measurements at different points on the campus according to the ASHRE standards (Aboelata 2021). The simulation process was run from midnight on 11th January 2022 to 6 a.m. on 12th January 2022 (30 h) to validate the 24 h, as shown in Table A 5. Six hours were added to the beginning of the simulation time to ensure the accuracy of the results. The comparison between measured and simulated AT, RH and MRT showed that they correlate well, as shown in Figure B 8. This validation depended on three metrics: Root Mean Square Error (RMSE), Mean Absolute Percentage Error (MAPE) and coefficient of determination or Linear regression coefficient (R2). A good Linear regression coefficient (R2) was between 0.9249–0.9484, 0.9744–0.9774 and 0.9249–0.9484 for AT, RH and MRT for different points, as shown in Figure B 9. Also, RMSE values between measured and simulated results were 0.88–0.968, 1.21–1.87 and 0.70–0.99 for AT, RH and MRT for different points which is a good relation compared to other studies (Morakinyo et al. 2018). For MAPE, the values were between 4.85–5.48%, 2.46–5.49% and 3.76–4.38% for AT, RH and MRT for different points, as shown in Table A 7. Finally, the validity of the model has been probed successfully.

### Survey of tree types

Several studies used 3d model trees from the Envi-met database in the simulation (Li et al. 2022). However, this study conducted a site survey of the common trees in NBAC which are Ficus family and Conocarpus. Ficus Benjamina is the most widespread tree type from the Ficus family which is distinguished by the high LAD with medium height. While Conocarpus is the most famous tree used as a windbreak in Egypt because of its columnar shape, high LAD and height (20 m). Therefore, this study used the Ficus Benjamina tree and the Conocarpus tree as windbreaks and built 3D models for these trees using the Albero section in ENVI-met V5.0.3 software. Table A 8 shows the used characteristics for these tree models. For LAD, it was found that low, medium and high LAD equals 0.5– 1.5, 2.0 and 5 (Tsoka et al. 2021). To investigate the effect of LAD, this study proposed to build the two trees using medium and high LAD values (2 and 5).

### Scenarios design

Thirty-two scenarios were proposed using two different trees to study the impact of different windbreak parameters, as shown in Table 1. Firstly, this study used the single and double lines of trees for each LAD of two different trees, as shown in Figure B 10. For tree spacing, this study investigated the small tree spacing which less, equal, and higher tree widths (2, 4 and 6 m), as shown in Figure B 11. For LAD, as mentioned before, this study proposed the investigation of medium and high LAD (2 and 5). Moreover, windbreak had an effective distance behind the trees depending on the tree height (Tamang et al. 2010). Due to the limited effect of windbreak on some areas on the campus, this study proposed eight different scenarios (S7, 8, 15, 16, 23, 24, 31 and 32) to investigate the impact of increasing the trees inside the campus with the borders. Windbreak in all scenarios was applied upwind direction which was coming from the southwest.

### Outdoor thermal comfort parameters

Physiological Equivalent Temperature (PET), AT and wind speed were used to assess the effect of windbreak on mitigating Cold stress during the morning (7.00) and night (23.00) and improving OTC during the afternoon (15.00). Like the measurements, the thermal index and parameters were simulated at 1.8 m height from the ground (Mahmoud et al. 2021). Physiological Equivalent Temperature (PET) is a thermal comfort index that is affected by different thermal parameters: AT, RH, MRT, windspeed, metabolic rate, and clothing insulation (Höppe 1999). Therefore, it is considered a representative index for OTC in many studies (Kumar & Sharma 2020), (Mahmoud et al. 2021), (Pecelj et al. 2021). This index has nine grades to express the level of thermal comfort from extremely cold to extremely hot stress (Höppe 1999), as shown in Table A 9. The PET distribution for the model area was analyzed, and the percentage area for every PET category was calculated in different scenarios at different times during the day (7.00, 15,00 and 23.00), as shown in Table A 11 and A 12. Reducing the cold PET grade (4–8°C) area was used as a representative parameter of the ability of windbreak scenarios to mitigate cold stress because of the high percentage of cold PET grade (4–8°C). However, in the afternoon time (15.00), The results of the PET analysis for different scenarios were between slightly cool, comfortable, warm and hot grades. Therefore, the results focus on the effect of different windbreak factors to increase the comfortable area and reduce the areas of warm and hot PET grades. Additionally, four main areas were observed inside the campus to investigate the impact of windbreak on wind speed and (AT) at the pedestrian level at different times compared to BCS. These areas are behind windbreak in the open area (Area 1), high H/W ratio (Area 2), the building’s corner (Area 3) windbreak behind WB in low H/W ratio (Area 4), as shown in Figure B 12.

## Results and discussion

### Impact of tree spacing

**Fig. 1:**
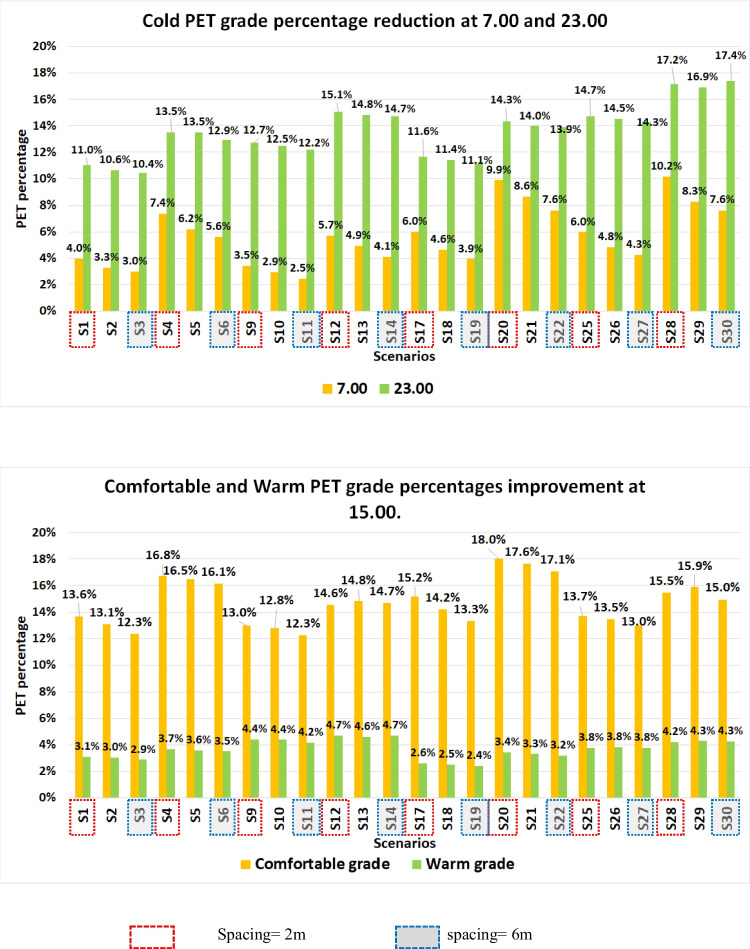
Cold, comfortable and warm PET grade area change percentage compared to BCS at different times. **a**. Cold PET grade area reduction percentage compared to BCS in the early morning (7.00) and night (23.00). **b**. Increasing comfortable grade area and reducing warm PET grade area for different scenarios compared to BCS at 15.00

Generally, reducing tree spacing less than the tee crown’s diameter proved its ability to reduce cold PET area inside the campus regardless of the tree type. Small Ficus tree spacing (2 m) reduced the cold PET area in the early morning and night up to 2.27% and 0.57% compared to high tree spacing (6 m). While these percentage slightly increased for Conocarpus tree in the morning and night up to 2.53% and 0.57%, respectively, as shown in Fig. 1a. This reduction occurred due to reducing the gaps or holes in the windbreak that ban the high wind speed from increasing the cold stress inside the campus. During the daytime, small Ficus tree spaces improved the comfortable PET grade area by 1.83% and reduced the warm PET area percentage up to 0.22% compared to high tree spacing due to increasing the shading, especially beside the windbreak. However, the same distance in Conocarpus scenarios had a limited effect on the same percentages only up to 0.70% and 0.23%, as shown in Fig. 1b. For example, reducing tree spacing to 2 m increased the area affected by increasing PET behind the tree belt compared to BCS up to 2.1°C and 2.4°C for S17 and S20, as shown in Figure B 13.

Small tree spacing for two trees reduced wind speed in the early morning and night in all focused areas (Area 1–4), especially in areas beside the buildings’ corners and in a high H/W ratio up to 1.5 m/s. However, this positive effect of windbreak was reduced in areas with a low H/W ratio up to only 0.75 m/s, as shown in Figure B 15. While using high tree spacing (4 and 6m) which more than Conocarpus’s diameter turned to a negative effect and increased the wind speed up to 0.5m/s due to the existence of the gaps between trees of the windbreak, as shown in Figure B 15.b. Regarding AT, small tree spacing for Ficus (2m) (S20) increased AT in areas with high H/W ratio (Area 2) at night and morning up to 0.65°C and 0.5°C, respectively compared to 0.5°C and 0.4°C for high tree spacing (6m) (S22), as shown Figure B 14.c. However, the impact of low Conocarpus tree spacing on increasing AT appeared far away from the windbreak position which could reach up to 0.7°C, while the increase behind the windbreak was only 0.3°C, as shown Figure B 14.d. During the noon, the impact of windbreak with small spaces led to reducing AT up to 1.2°C, especially on the west side of the campus due to the provided windbreak shading compared to lower impact due to high tree spaces.

Finally, small tree spacing less than the tree diameter makes an overlap between the trees’ crowns which increases the windbreak density against the high wind speed at night and increases the shading during the noon time. This mitigating effect of cold stress appeared behind the windbreak and in narrow spaces between buildings. These results complied with previous studies that addressed the importance of reducing the tree spacing up to 0.5m (Chang et al. 2021), (Jian et al. 2018), (Bitog et al. 2012).

### Impact of the number of trees rows

Double tree rows could reduce the cold PET grade area by 10.15% and 17.15% at 7.00 and 23.00 compared to BCS, as shown in Figure B 16.a. This positive impact increased with the Conocarpus more than Ficus tree due to Conocarpus’s big height. During the afternoon, Double rows increased the comfortable PET area by 18.04% and reduced the warm PET area up to 4.69% compared to BCS, as shown in Figure B 16.b. However, Ficus tree had the advantage of increasing the Comfortable area more than Conocarpus due to its crown shape which increased the shading. For example, the reduction of PET reached up to 2.4°C for double rows compared to 2.1°C for single rows, as shown in Figure B 17.

Regarding windspeed, using double tree rows reduced the wind speed for different scenarios at all different times, and maximized the areas behind the tree belt affected by windbreak up to 40 m. Double rows of Ficus trees reduced wind speed in the early morning and night up to 1.5m/s behind the windbreak compared to only 1.25m/s for a single row. This reduction in wind speed is slightly reduced for Conocarpus scenarios, as shown in Figure B 18. This reduction in wind speed appeared clearly in all investigated areas (Area 1–4) inside the campus, especially in areas with high H/W ratios and around the building corners. For AT, double Ficus tree rows increased AT in the morning up to 0.5°C compared to 0.3°C for a single-row scenario. This increase in AT at night due to double rows of trees reduced up to 0.3°C compared to 0.2°C inside most areas of campus. However, the double Conocarpus rows are more effective in increasing AT compared to Ficus tree. The AT increase reached above 0.6°C and 0.5°C for double and single-row scenarios, as shown in Figure B 19.

Finally, the Ficus double row achieved the best results in reducing cold stress at night and improving comfortable area percentage during the daytime. However, increasing tree rows of the columnar tree hadn’t a massive effect on increasing the comfortable area during daytime. Moreover, during the afternoon, double tree rows with high tree spacing were slightly better in increasing the comfortable percentage compared to small tree spacing. To our best knowledge, it was the first result that linked the privilege of increasing tree rows with different parameters.

### Impact of trees’ distribution

This section focuses on the eight scenarios that increase the urban trees inside the campus (S7, 8, 15, 16, 23, 24, 31 and 32). Increasing trees inside the campus could decrease the cold PET grade area percentages compared to BCS at 7.00 and 23.00 up to 19.31% (S24) and 18.06% (S31), respectively, as shown in Figure B 20.a. Also, these scenarios increased the comfortable PET area and reduced the warm PET area compared to BCS up to 25.21% (S23) and 5.36% (S15), respectively, as shown in Figure B 20.b. Therefore, Conocarpus on the borders and inside the site (S31) achieved the best results in mitigating cold stress at night. However, Ficus trees inside and on the borders (S23) achieved a remarkable effect on increasing comfortable PET grade area percentage during the afternoon. For example, increasing trees inside the campus (S23) increased PET in the early morning between 1.2- 2.4°C in most of the campus area. While this tree distribution reduced PET up to 9°C at noon time in open areas due to three shades, however, the areas behind the building may suffer from increasing the PET due to reducing wind speed which mitigated the heat stress at noon time, as shown in Figure B 21. Regarding windspeed, planting Ficus trees inside and on the border of the campus (S23) achieved maximum wind speed reduction during early morning, afternoon and night up to 2.47 m/s, 2.43 m/s and 2.50 m/s, respectively, as shown in Figure B 22. However, S 24 achieved the maximum increase in (AT) at the pedestrian level during morning and night up to 1.18°C and 0.79°C, respectively, as shown in Figure B 23.

Generally, using Ficus trees with high LAD inside and on the site’s borders is the most suitable scenario to improve the comfortable area during the afternoon and reduce the cold stress in the morning. However, using Conocarpus with high LAD inside and on the site’s borders had a better effect on mitigating cold stress at night. These results provide guidance to deal with the weak areas that needed more protection in the residential campus in hot climates.

### Impact of the leaf area density (LAD)

This section compared S7, S15, S23, and S31 to investigate the effect of increasing LAD to mitigate cold stress. High LAD reduced the cold PET area percentage in the early morning and night compared to BCS by (18.90% and 15.54%) and (18.95% and 18.06%) for S23 and S31, respectively. However, this percentage massively reduced for medium LAD compared to BCS by (13.78% and 14.64%) and (11.47% and 16.57%) for S7 and S15, respectively. During the afternoon, high LAD increased the comfortable area percentage compared to BCS by 25.21% and 19.30% for S23 and S31, respectively. However, medium LAD achieved a lower percentage compared to BCS by 22.51% and 17.60% for S7 and S15, respectively, as shown in Figure B 20.

For Windspeed, increasing LAD increased the maximum wind speed reduction at 7.00 from 2.20m/s to 2.47m/s and from 1.98m/s to 2.27m/s for Ficus and Conocarpus, respectively. This positive effect slightly increased at night between 0.03- 0.11m/s. However, this positive effect on reducing wind speed was the smallest during the daytime. For example, Using Ficus tree with high LAD (S23) achieved the maximum mitigation on wind speed inside most area of the campus between 1.25- 2.25m/s compared to 0.75- 1.5 m/s for the same tree distribution with medium LAD (S7), as shown in Figure B 24. For AT, high LAD increased the maximum AT in the early morning and night by (1.06°C and 0.72°C) and (1.09°C and 0.74°C) for S23 and S31, respectively. However, the medium LAD had a smaller effect on the maximum AT reduction in the early morning and night by (0.77°C and 0.55°C) and (0.74°C and 0.50°C) for S7 and S15, respectively. For example, the average AT in most area of campus increased in the morning and night by 0.5°C and 0.4°C for high LAD (S31) compared to 0.3°C and 0.2°C for medium LAD (S15), as shown in Fig. 25.

### Impact of shape and height of the trees

**Fig. 2:**
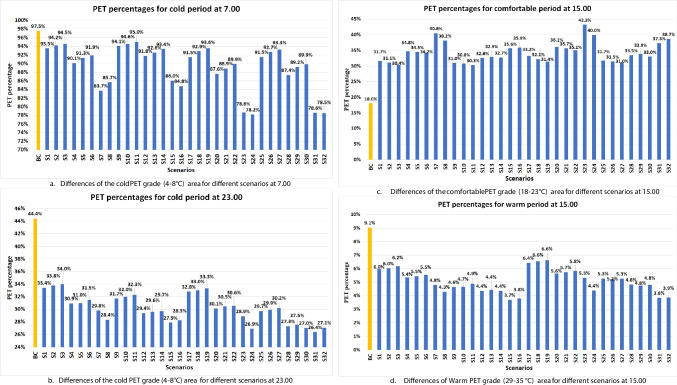
Differences in area for Cold PET grade at 7.00 and 23.00, the comfortable PET grade (18–23 °C) and the warm PET grade (29–35 °C) at 15.00. **a**. Differences of the cold PET grade (4–8 °C) area for different scenarios at 7.00. **b**. Differences of the cold PET grade (4–8 °C) area for different scenarios at 23.00. **c**. Differences of the comfortable PET grade (18–23 °C) area for different scenarios at 15.00. **d**. Differences of Warm PET grade (29–35 °C) area for different scenarios at 15.00

Ficus With round shape and medium LAD achieved cold stress PET area reduction between 2.98–13.78% and 10.43–14.64% for S1-7 at 7.00 and 23.00, respectively. However, the cold stress reduction due to using Conocarpus columnar shape tree with the same LAD ranged between 2.47%- 11.47% and 12.17–16.57% for S9-15 at 7.00 and 23.00, respectively. For high LAD, Ficus tree could reduce cold PET area between 3.94- 18.90% and 11.11- 15.54% for S 17–23 at 7.00 and 23.00, respectively. However, Conocarpus tree achieved cold PET area reduction ranging between 4.25- 18.95% and 14.26- 18.06% 7.00 and 23.00, respectively, as shown in Fig. 2a, b. During the afternoon, Ficus improved the comfortable PET area percentage up to 22.51% and 25.21% for medium and high LAD, respectively. However, Conocarpus could increase the comfortable PET area only up to 17.60% and 19.30% for medium and high LAD, respectively, as shown in Fig. 2c, d. For windspeed, Ficus round shape tree with medium LAD could reduce wind speed up to 2.28m/s, 2.24m/s and 2.28m/s at 7.00, 15.00 and 23.00, respectively. However, Conocarpus columnar shape tree with the same LAD only reduced wind speed up to 2.08m/s, 2.03m/s and 2.09m/s at the same times. Also, these privilege of Ficus trees was repeated with high LAD scenarios. Moreover, the columnar shape of the tree with high tree spaces more than the tree’s diameter led to an increase in the wind speed between these trees. For the effective range of windbreak, the results found that the effect of windbreak on reducing wind speed extended to 1–2 times of tree height between 20 to 40m, but wind speed increased again beyond this distance, as shown in Figure B 24. For AT, Ficus could increase maximum AT during early morning and night up to 0.77°C and 0.55°C for medium LAD and up to 1.06°C and 0.72°C for high LAD. However, Conocarpus could increase maximum AT during early morning and night up to 0.74°C and 0.50°C for medium LAD and up to 1.09°C and 0.74°C for high LAD.

### The order of the effective factors

Regarding the significance of differences in reducing cold PET grades for different scenarios, reducing tree spacing from 4 to 2 m by approximately is more effective in reducing cold PET grade than reducing spacing from 6 to 4 m. For increasing tree rows, the effect of increasing additional tree rows is slightly more effective in reducing cold PET grade for round medium-height trees (Ficus) than columnar and tall trees (Conocarpus), as shown in Figure B 16.a. For LAD, increasing the density of tree leaves is more effective in reducing the area of cold PET grade for tall trees (Conocarpus) than medium-height trees (Ficus).

As a result, it was found that the most effective order of windbreak parameters to reduce cold stress in the early morning was tree distribution, LAD, Number of tree rows, tree spacing and tree shape for two tree types. Also, this order repeated for Ficus tree at night, however, using tall columnar trees made the most effective order was the number of rows, shape, LAD, tree distribution and tree spacing, as shown in Figure B 26. For increasing OTC during the daytime, the order became tree distribution, shape, number of rows, LAD and tree spacing, as shown in Figure B 27. Finally, Ficus tree proved its efficiency at night and daytime, however, Conocarpus tree gave better results at night.

### Performance of best winter scenarios under summer conditions

**Fig. 3:**
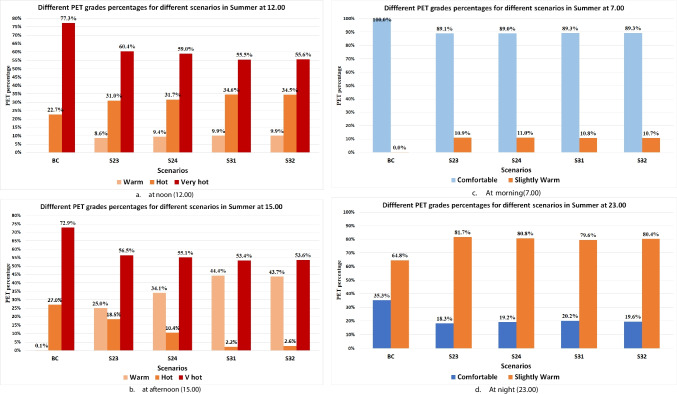
The percentage of the area inside the campus of different PET grades for best winter scenarios during summer at 12.00, 15.00, 7.00 and 23.00. **a**. at noon (12.00) **b**. at afternoon (15.00) **c**. At morning (7.00) d. At night (23.00)

Four scenarios (S23, 24, 31 and 32) achieved the best performance of mitigating cold stress at morning and night with improving thermal comfort during the afternoon. Therefore, these scenarios with BCS were simulated under summer conditions. Generally, all scenarios reduced the very hot PET area percentage in the afternoon (15.00) more than noon (12.00) due to the increase in tree shading. Also, Conocarpus scenarios (S31 and 32) reduced very hot PET area at 12.00 compared to BCS by 21.79% and 21.66%, respectively. While Ficus scenarios (S23 and 24) reduced very hot PET percentage only by 16.90% and 18.28%, respectively, as shown in Fig. 3a. In the afternoon, Conocarpus scenarios (S31 and 32) reduced very hot PET grade percentage by 19.5% and 19.27%, respectively, and reduced hot PET grade area by 24.83% and 24.38%, respectively, as shown in Fig. 3b. Therefore, Concarpous has a higher positive effect on mitigating summer conditions than Ficus, especially at noon (12.00) and provides shade for the whole campus area during the afternoon. For the comfortable grade area, all four scenarios had a negative effect during morning and night, which increased the warm PET grade area approximately by 11% and 16%, respectively, as shown in Fig. 3c, d. These results proved the importance of tree distribution and tree features especially LAD and tree height parameters in mitigating summer conditions.

### Environmental impact on GHG emissions

In our model, the number of trees was 188, 376 and 465 trees for single row, double rows and double rows with trees inside the campus scenarios, respectively. Ficus and Conocarpus trees could remove 21.7 kg and 31.6 kg of CO_2_ emissions annually, respectively (Mohamedmeki & Almumaiz 2021). In this regard, the annual cost of different methodologies to remove CO_2_ is between 94- 232 US$/ ton (0.094- 0.232 US$/ Kg) (Reardon 2018). While this study depends on the average cost which was 0.163 US$/ Kg. The annual amount of removed CO_2_ emission and CO_2_ removal cost for different scenarios were calculated, as shown in Table A 10. The results found that Conocarpus trees scenarios (S15, 31) achieved the highest amount of CO_2_ sequestration and economic saving by 14.69 Tons and 2351 US$, respectively. These scenarios were followed by the mixed scenarios between Conocarpus and Ficus trees inside and on the site borders. Also, these removal amounts and cost savings could be increased by replacing the grey concrete, which represents 56.3% of the campus area, with grass cover. This increase could be achieved due to the ability of grass cover to absorb 0.28 kg of CO_2_ emission/m^2^ (Kongsager et al. 2013).

## Limitation and future work

This study had different limitations which could be investigated in future studies such as:

- This simulation process put the wind speed as a constant equal to the average measured wind speed during all day. As a result, a full forcing simulation process with hourly wind speed inputs needs to be simulated.
- The wind direction in all different scenarios was fixed coming from the southwest like the real situation. Windbreak was applied upwind direction which is the most effective direction to protect the targeted spaces, based on previous investigations (Taleb & Kayed 2021), (Tamang et al. 2010), (Baker et al. 2021). However, different wind directions need to be investigated to evaluate the effect of WB on thermal conditions.
- The effect of the best WB configuration could be investigated inside different climate regions to evaluate the grade of efficiency of WB in mitigating cold stress in different climates.
- This study mainly focused on improving OTC using windbreak strategy under cold and hot conditions, but also other green solutions could be integrated to enhance the OTC improvement.
- The positive impact of windbreak on microclimate conditions needs to be studied to improve indoor thermal comfort and energy efficiency.

## Conclusion

This study presents a numerical investigation to improve microclimate conditions during cold weather by investigating different windbreak factors’ effect in hot climate on various OTC parameters such as PET, (AT) and wind speed. The main findings of this study are:

- Small tree spacing less than tree diameter had a massive positive effect on reducing cold stress and wind speed which improve OTC in cold mornings and nights. However, high tree spacing more than tree diameter produces gabs in windbreak which increase the windspeed, especially for columnar trees.
- Increasing number of rows mitigates cold stress, especially in early mornings more than at night. Also, this increase had a slightly positive impact on increasing comfortable during the daytime, especially for round trees.
- High LAD trees achieved better results in all different scenarios in mitigated cold microclimate conditions compared to medium LAD.
- The order of the most effective windbreak parameters to mitigate cold stress are tree distribution, LAD, number of tree rows, tree spacing and finally the tree shape.
- The optimum windbreak design guidelines were using very dense trees with medium height formed in double rows with the smallest possible tree spacing inside and on the site’s borders.
- The effect of windbreak on OTC parameters increased in the spaces with low H/W ratio, behind the windbreak and on the edge of the buildings, however, the lowest affected areas were spaces behind the building according to wind direction and outdoor spaces-oriented East–West.
- Windbreak increased the shading percentage inside the campus which improved OTC during the summer season, especially in the afternoon time, however, windbreak increased the outdoor (AT) and PET over comfortable limits during the night.
- The high LAD for Conocarpus trees achieved the highest carbon sequestration amount and economic saving compared to Ficus trees.

### Supplementary Information

Below is the link to the electronic supplementary material.

Supplementary file1 (DOCX 9303 KB)

## Data Availability

The authors confirm that the data supporting the findings of this study are available within the article and its supplementary materials.
