# Supplementary material for: Impact of windbreak design on microclimate in hot regions during cold waves: Numerical investigation
Source: Int J Biometeorol. 2024 May 6;68(7):1315–26. doi: 10.1007/s00484-024-02668-8 (PMC11272679; doi:10.1007/s00484-024-02668-8)
Supplement: Supplementary file 1 — Supplementary file1 (DOCX 9303 KB) [file 484_2024_2668_MOESM1_ESM.docx]

**Appendix A**

Table A 1. Previous WB studies for different investigated points

| Ref | Climate | Aim | | | | | Method | | | | WB factors | | | | | | | | | | Time | | Parameters | | | | | | | | |
| --- | --- | --- | --- | --- | --- | --- | --- | --- | --- | --- | --- | --- | --- | --- | --- | --- | --- | --- | --- | --- | --- | --- | --- | --- | --- | --- | --- | --- | --- | --- | --- |
|  |  | Wind protection | Animal thermal comfort | Human Thermal comfort | Air quality & soil | Crops yield & soil | Real case | Experiment | Mathematical | Software | WB type | Rows No. | Tree type | Tree shape | LAD | Tree height | Tree spacing | Trees number | Tree distribution | WB orientation | Summer | Winter | Wind speed | Air temperature | Humidity | PET | Solar radiation | Land temperature | PM | Snow depth |  |
| (Wu et al., 2022) | Artificial | √ |  |  |  |  |  |  |  | √ |  | √ | √ |  |  |  |  |  |  |  | --- | --- | √ |  |  |  |  |  |  |  |  |
| (Chang et al., 2019) | Cold climate | √ |  |  | √ |  | √ |  |  |  |  | √ |  |  | √ | √ |  | √ |  |  | √ |  | √ |  |  |  |  |  | √ |  |  |
| (Chang et al., 2021) | Cold climate | √ |  |  | √ |  | √ |  |  |  | √ |  |  |  |  |  |  |  | √ |  | √ |  | √ |  |  |  |  |  | √ |  |  |
| (He et al., 2017) | Warm climate |  | √ |  |  |  | √ |  | √ |  |  |  |  |  |  |  |  |  |  |  | √ |  | √ | √ |  |  |  |  |  |  |  |
| (Iwasaki et al., 2021) | Cool humid climate |  |  |  |  | √ |  |  |  | √ |  |  |  |  |  | √ |  |  |  | √ | √ |  | √ |  |  |  | √ | √ |  |  |  |
| (Taleb & Kayed, 2021) | Hot arid climate |  |  |  | √ |  |  |  |  | √ |  |  |  |  |  |  | √ |  |  |  | √ |  |  |  |  |  |  |  | √ |  |  |
| (Ma et al., 2019) | Laboratory experiment | √ |  |  |  |  |  | √ |  |  |  |  |  |  |  |  |  |  |  |  | --- | --- | √ |  |  |  |  |  |  |  |  |
| (Jian et al., 2018) | Artificial | √ |  |  |  |  |  |  |  | √ |  |  |  | √ |  |  | √ |  |  |  | --- | --- | √ |  |  |  |  |  |  |  |  |
| (J. Li & Dong, 2021) | Laboratory experiment | √ |  |  |  | √ |  | √ |  |  |  |  | √ |  | √ |  |  |  |  |  | --- | --- | √ |  |  |  |  |  |  |  |  |
| (Moskovaya et al., 2017) | Cold climate |  |  |  | √ |  |  |  |  |  |  |  |  |  |  |  |  |  | √ |  |  | √ | √ |  |  |  |  |  | √ |  |  |
| (Yukhnovskyi et al., 2021) | Cold climate | √ |  |  |  |  | √ |  |  |  |  |  |  | √ | √ |  |  |  |  |  | √ | √ | √ |  |  |  |  |  |  |  |  |
| (Wang et al., 2019) | Hot and cold climates |  |  | √ |  |  | √ |  |  |  |  |  |  |  | √ |  |  |  |  |  | √ | √ |  |  |  |  |  | √ |  |  |  |
| (Bitog et al., 2012) | Warm climate | √ |  |  |  |  |  | √ |  | √ |  | √ |  |  |  |  | √ |  | √ |  | --- | --- | √ |  |  |  |  |  |  |  |  |
| (X. Li et al., 2022) | humid subtropical climate |  |  | √ |  |  |  |  |  | √ |  |  |  |  | √ |  |  |  |  |  |  | √ | √ | √ |  |  |  |  |  |  |  |
| (Girona et al., 2019) | Cold climate |  |  |  |  | √ | √ |  |  |  |  |  |  |  |  |  |  |  |  |  |  | √ |  |  |  |  |  |  |  | √ |  |
| (Tamang et al., 2010) | Equatorial climate | √ |  |  |  | √ | √ |  |  |  |  |  | √ |  |  |  |  |  |  |  | √ | √ | √ | √ | √ |  |  |  |  |  |  |
| (Baker et al., 2021) | Temperate climate | √ |  |  |  |  | √ |  |  |  |  |  |  |  |  |  |  |  |  |  | √ | √ | √ | √ | √ |  |  |  |  |  |  |
| (Id et al., 2020) | Laboratory experiment | √ |  |  |  |  |  | √ |  |  |  |  |  | √ |  |  |  |  |  |  | --- | --- | √ |  |  |  |  |  |  |  |  |

Table A 2. The urban characteristics for the northern E-JUST residential campus in NBAC.

| 1. Urban characteristics |  |
| --- | --- |
| Building dimensions | 18.6/21.8× 18.5 m |
| Building height | 16.5 m (Five floors) |
| Single building Area | 303.8 m^2^ (Fourteen buildings) |
| Number of apartments/ buildings | 20 apartments/ building |
| Total building area ratio | 4253/17000 = 25% |
| Playground area ratio | 2458/17000= 14.5% |
| Grey concrete pavement ratio | 9569/17000= 56.3% |
| Green area ratio | 720/17000= 4.2% |
| 1. Material characteristics |  |
| Street | Asphalt |
| Green area type | Small bushes with sandy and gravel soil |
| Soil type | Sandy soil |
| Pavement | Light gray concrete pavement |
| Façade | Beige paint |
| Roof | White ceramic tiles |

Table A 3. The description of measurement points in the residential campus.

| Point | Ground | H/W ratio | Orientation |
| --- | --- | --- | --- |
| P1 | Gray concrete | 16.5/13 (1.25) | Southwest-Northeast (240^o^) |
| P2 | Gray concrete | 16.5/11 (1.5) | Northwest- Southeast (330^o^) |
| P3 | Gray concrete | 16.5/16.5 (1) | Southwest-Northeast (330^o^) |

Table A 4. Different measurement tools were used in this study.

| No | Sensor | Variables | Range | Accuracy | Measuring height | Time intervals |
| --- | --- | --- | --- | --- | --- | --- |
| 1 | Testo 174h logger | Out T_a_ &RH | -20- 70 °C | ± 0.5 °C | 1.5 m | 15 min |
| 2 | Testo 440 CO_2_ Kit with Bluetooth | Outdoor T_a_ | -40 to +150 °C | ±0.3 °C (-25 to +74.9 °C) | 1.5 m | Every hour |
|  |  | Outdoor RH | 5- 95% | ±0.6% RH |  |  |
|  |  | CO_2_ level | 0- 10000 ppm | ±(50 ppm + 3 % of mv) (0 to 5000 ppm) |  |  |
| 3 | Globe thermometer for radiant heat | MRT | 0- 120 °C | --- | 1.5 m | hourly |
| 4 | Digital Anemometer | Windspeed | 0-150 Km/h | --- | 1.5 m | hourly |

Table A 5. The Microclimate parameters inputs on ENVI-met simulation.

| Parameter | Value |
| --- | --- |
| Location | New Borg El-Arab City, Alexandria, Egypt |
| Position | Latitude 30°85', Longitude 29°59' |
| Date | 11/1/2022 – 12/1/2022 |
| Start time | 00.00 on 11^th^ January 2022 |
| End time | 6.00 on 12^th^ January 2022 |
| Simulation period | 30 hours |
| Wind speed at 1.4 m | 3.6 m/s |
| Wind speed at 10 m (calculated) | 3.67 m/s |
| Wind direction | 160° |
| Air temperature | 8.8 °C (minimum) and 23.3 °C (maximum) |
| Relative humidity | 16.9% (minimum) and 59.9% (maximum) |
| Model size | 140× 110× 30 grids |
| Grid cell size | 2 × 2× 2 m |

Table A 6. The used material characteristics inside the model.

| Construction & Plant | Material | Input parameter | values |
| --- | --- | --- | --- |
| Building facades (5 floors height) | Red brick wall with mortar and paints | Thickness | 0.28 |
|  |  | albedo | 0.80 |
|  |  | Emissivity | 0.85 |
| Building roofs | Concrete roof with grey roofing tiles | Thickness | 0.27 |
|  |  | Albedo | 0.50 |
|  |  | Emissivity | 0.90 |
| Street | Asphalt (Default) | albedo | 0.20 |
|  |  | emissivity | 0.90 |
| Green area type | Green area | Plant height | 0.05 |
|  |  | albedo | 0.15 |
|  |  | Transmittance | 0.30 |
|  |  | Leaf type | Grass |
|  |  | LAI | 1.5 |
| Soil type | Sandy soil (Default) | albedo | 0.10 |
|  |  | emissivity | 0.90 |
| Street Pavement | Concrete Pavement Grey | Thickness | 0.10 |
|  |  | albedo | 0.50 |
|  |  | emissivity | 0.90 |
| Campus Pavement | Interlock grey | Thickness | 0.10 |
|  |  | albedo | 0.850 |
|  |  | emissivity | 0.90 |

Table A 7. different validation metrics (RMSE, MAPE, R^2^)

|  | AT | | | RH | | | MRT | | | Acceptable range (Aboelata, 2021) |
| --- | --- | --- | --- | --- | --- | --- | --- | --- | --- | --- |
|  | P1 | P2 | P3 | P1 | P2 | P3 | P1 | P2 | P3 |  |
| Root Mean Square Error: RMSE | 0.88 | 0.96 | 0.97 | 1.42 | 1.21 | 1.87 | 0.86 | 0.70 | 0.99 | (RMSE) < 30% |
| Mean Absolute Percentage Error: MAPE | 4.85 | 5.48 | 5.33 | 3.47 | 2.46 | 5.49 | 4.38 | 3.98 | 3.76 | MABE < 10% |
| coefficient of determination (R2) | 0.9453 | 0.9484 | 0.9249 | 0.9774 | 0.9744 | 0.9760 | 0.9453 | 0.9484 | 0.9249 | R2> 0.7 |

Table A 8. Different modeling information of used trees

| Parameter | Ficus Benjamina | | Conocarpus | |
| --- | --- | --- | --- | --- |
| Leaf type | Deciduous | | Deciduous | |
| Shape | Rounded shape | | Columnar shape | |
| Tree height | 12m | | 20m | |
| Tree width | 5m | | 2m | |
| Root depth | 1m | | 0.5m | |
| Foliage albedo | 0.18 | | 0.18 | |
| LAD | 2.5, 0.5 | | 2.5, 0.5 | |
| Model | 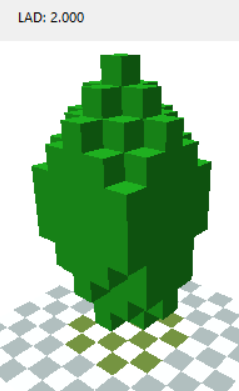 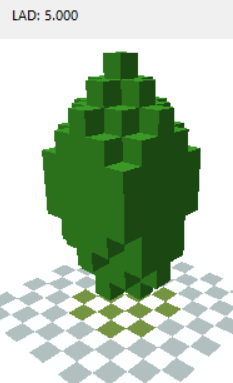 | | 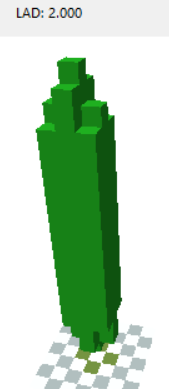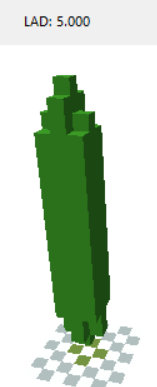 | |
| Real shape | 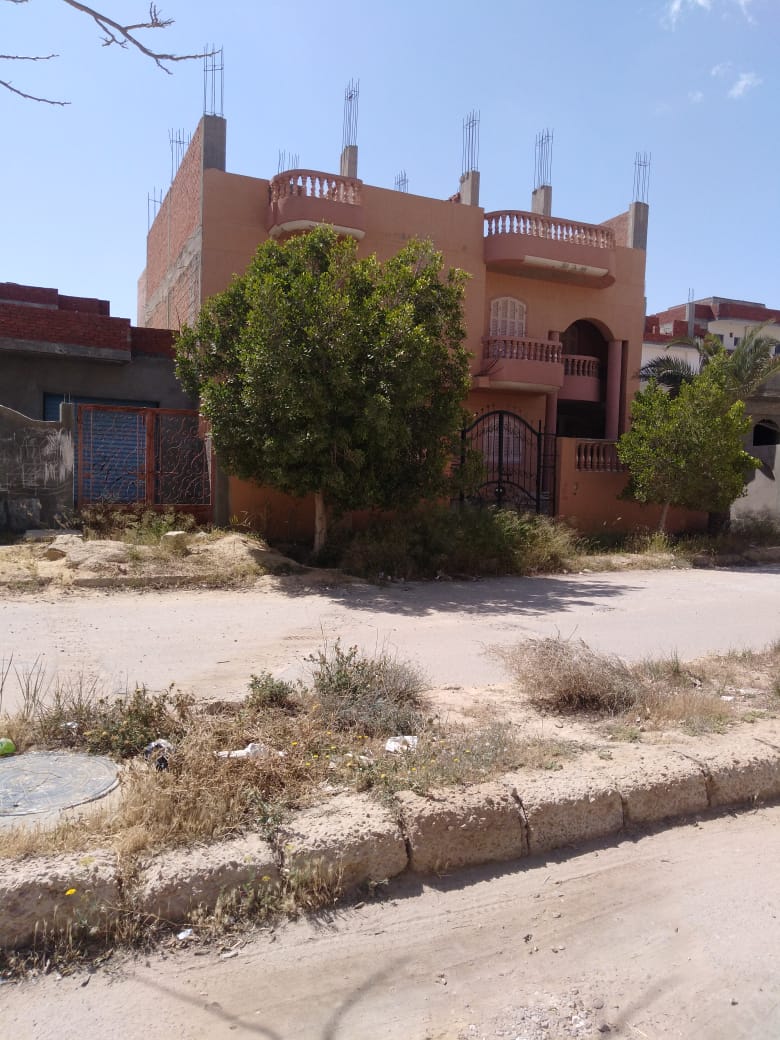 | 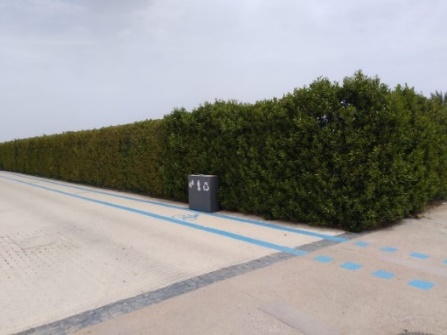 | |  |

Table A 9. Thermal perception and stress classification of Physiological Equivalent Temperature (PET) (Peter Höppe, 1999).

| **PET (°C)** | **Grade** |
| --- | --- |
| ˂ 4 °C | Extreme Cold Stress (Very cold) |
| 4-8 °C | Strong Cold Stress (Cold) |
| 8-13 °C | Moderate Cold Stress (Cool) |
| 13-18 °C | Slight Cold Stress (Slightly cool) |
| 18-23 °C | No Thermal Stress (Comfortable) |
| 23-29 °C | Slight Heat Stress (Slightly warm) |
| 29-35 °C | Moderate Heat Stress (Warm) |
| 35-41 °C | Strong Heat Stress (Hot) |
| ˃ 41°C | Extreme Heat Stress (Very Hot) |

Table A 10. Yearly total carbon removal and economic saving for the highest tree number scenarios.

| Sc. | No Ficus | No Conocarpus | Total no of trees | CO_2_ removal/ tree | | Total CO_2_ removal | Economic Saving  (0.16 US$/Kg) |
| --- | --- | --- | --- | --- | --- | --- | --- |
|  |  |  |  | 21.7Kg (Ficus) | 31.6Kg (Conocarpus) |  |  |
| S1, 17 | 188 | 0 | 188 | 4.08Ton | --- | 4.08Ton | 652.7 US$ |
| S4, 20 | 376 | 0 | 376 | 8.16Ton | --- | 8.16Ton | 1305.5 US$ |
| S9, 25 | 0 | 188 | 188 | --- | 5.94Ton | 5.94Ton | 950.5 US$ |
| S12, 28 | 0 | 376 | 376 | --- | 11.88Ton | 11.88Ton | 1901.1 US$ |
| S7, 23 | 465 | 0 | 465 | 10.09Ton | --- | 10.09Ton | 1614.5 US$ |
| S8, 24 | 277 | 188 | 465 | 6.01Ton | 5.94Ton | 11.95Ton | 1912.1 US$ |
| S15, 31 | 0 | 465 | 465 | --- | 14.69Ton | 14.69Ton | 2351 US$ |
| S16, 32 | 188 | 277 | 465 | 4.08Ton | 8.75Ton | 12.83Ton | 2053.2 US$ |

Table A 11. The area of different PET grades during cold winter day at different times (7.00, 15.00 and 23.00).

| No | PET grade | | | 7.00 | | 15.00 | | 23.00 | | No | PET grade | | 7.00 | 15.00 | 23.00 |
| --- | --- | --- | --- | --- | --- | --- | --- | --- | --- | --- | --- | --- | --- | --- | --- |
| Base case | very cold | below 4 | 0.01% | | 0 | | 0 | |  | | | | | | |
|  | Cold | 4-8 | 97.50% | | 44.44% | | 44.44% | |  |  |  |  |  |  |  |
|  | Cool | 8-13 | 2.49% | | 55.56% | | 55.56% | |  |  |  |  |  |  |  |
|  | Slightly cool | 13-18 | 0 | | 0 | | 0 | |  |  |  |  |  |  |  |
| S1 | very cold | below 4 | 0 | | 0 | | 0 | | S17 | | very cold | below 4 | 0 | 0 | 0 |
|  | Cold | 4-8 | 93.53% | | 33.44% | | 83.51% | |  |  | Cold | 4-8 | 91.48% | 32.81% | 82.45% |
|  | Cool | 8-13 | 6.47% | | 66.55% | | 16.49% | |  |  | Cool | 8-13 | 8.51% | 67.17% | 17.55% |
|  | Slightly cool | 13-18 | 0 | | 0.01% | | 0 | |  |  | Slightly cool | 13-18 | 0 | 0.02% | 0 |
| S2 | very cold | below 4 | 0 | | 0 | | 0 | | S18 | | very cold | below 4 | 0 | 0 | 0 |
|  | Cold | 4-8 | 94.21% | | 33.80% | | 84.51% | |  |  | Cold | 4-8 | 92.86% | 33.03% | 83.33% |
|  | Cool | 8-13 | 5.78% | | 66.19% | | 15.49% | |  |  | Cool | 8-13 | 7.14% | 66.97% | 16.66% |
|  | Slightly cool | 13-18 | 0 | | 0.01% | | 0 | |  |  | Slightly cool | 13-18 | 0 | 0 | 0 |
| S3 | very cold | below 4 | 0 | | 0 | | 0 | | S19 | | very cold | below 4 | 0 | 0 | 0 |
|  | Cold | 4-8 | 94.52% | | 34.01% | | 85.04% | |  |  | Cold | 4-8 | 93.56% | 33.33% | 83.82% |
|  | Cool | 8-13 | 5.49% | | 65.97% | | 14.96% | |  |  | Cool | 8-13 | 6.44% | 66.67% | 16.18% |
|  | Slightly cool | 13-18 | 0 | | 0.02% | | 0 | |  |  | Slightly cool | 13-18 | 0 | 0 | 0 |
| S4 | very cold | below 4 | 0 | | 0 | | 0 | | S20 | | very cold | below 4 | 0 | 0 | 0 |
|  | Cold | 4-8 | 90.12% | | 30.93% | | 79.92% | |  |  | Cold | 4-8 | 87.62% | 30.10% | 78.07% |
|  | Cool | 8-13 | 9.88% | | 69.07% | | 20.08% | |  |  | Cool | 8-13 | 12.38% | 69.89% | 21.93% |
|  | Slightly cool | 13-18 | 0 | | 0 | | 0 | |  |  | Slightly cool | 13-18 | 0 | 0.01% | 0 |
| S5 | very cold | below 4 | 0 | | 0 | | 0 | | S21 | | very cold | below 4 | 0.01% | 0 | 0 |
|  | Cold | 4-8 | 91.30% | | 30.95% | | 80.75% | |  |  | Cold | 4-8 | 88.87% | 30.47% | 79.23% |
|  | Cool | 8-13 | 8.70% | | 69.05% | | 19.25% | |  |  | Cool | 8-13 | 11.12% | 69.51% | 20.77% |
|  | Slightly cool | 13-18 | 0 | | 0 | | 0 | |  |  | Slightly cool | 13-18 | 0 | 0.02% | 0 |
| S6 | very cold | below 4 | 0 | | 0 | | 0 | | S22 | | very cold | below 4 | 0 | 0 | 0 |
|  | Cold | 4-8 | 91.88% | | 31.50% | | 81.18% | |  |  | Cold | 4-8 | 89.89% | 30.55% | 79.90% |
|  | Cool | 8-13 | 8.12% | | 68.50% | | 18.82% | |  |  | Cool | 8-13 | 10.11% | 64.46% | 20.10% |
|  | Slightly cool | 13-18 | 0 | | 0 | | 0 | |  |  | Slightly cool | 13-18 | 0 | 0 | 0 |
| S7 | very cold | below 4 | 0 | | 0 | | 0 | | S23 | | very cold | below 4 | 0 | 0 | 0 |
|  | Cold | 4-8 | 83.72% | | 29.80% | | 70.18% | |  |  | Cold | 4-8 | 78.60% | 28.90% | 66.36% |
|  | Cool | 8-13 | 16.28% | | 70.20% | | 29.82% | |  |  | Cool | 8-13 | 21.40% | 71.03% | 33.64% |
|  | Slightly cool | 13-18 | 0 | | 0 | | 0 | |  |  | Slightly cool | 13-18 | 0 | 0.07% | 0 |
| S8 | very cold | below 4 | 0 | | 0 | | 0 | | S24 | | very cold | below 4 | 0 | 0 | 0 |
|  | Cold | 4-8 | 85.66% | | 28.36% | | 70.85% | |  |  | Cold | 4-8 | 78.19% | 26.90% | 65.33% |
|  | Cool | 8-13 | 14.34% | | 71.63% | | 29.15% | |  |  | Cool | 8-13 | 21.81% | 73.03% | 34.67% |
|  | Slightly cool | 13-18 | 0 | | 0.01% | | 0 | |  |  | Slightly cool | 13-18 | 0 | 0.07% | 0 |
| S9 | very cold | below 4 | 0 | | 0 | | 0 | | S25 | | very cold | below 4 | 0.01% | 0 | 0 |
|  | Cold | 4-8 | 94.05% | | 31.70% | | 84.17% | |  |  | Cold | 4-8 | 91.51% | 29.70% | 80.00% |
|  | Cool | 8-13 | 5.95% | | 68.28% | | 15.83% | |  |  | Cool | 8-13 | 8.48% | 70.30% | 20.00% |
|  | Slightly cool | 13-18 | 0 | | 0.02% | | 0 | |  |  | Slightly cool | 13-18 | 0 | 0 | 0 |
| S10 | very cold | below 4 | 0 | | 0 | | 0 | | S26 | | very cold | below 4 | 0.01% | 0 | 0 |
|  | Cold | 4-8 | 94.57% | | 31.97% | | 85.20% | |  |  | Cold | 4-8 | 92.67% | 29.91% | 81.35% |
|  | Cool | 8-13 | 5.43% | | 68.02% | | 14.80% | |  |  | Cool | 8-13 | 7.32% | 70.09% | 18.66% |
|  | Slightly cool | 13-18 | 0 | | 0.01% | | 0 | |  |  | Slightly cool | 13-18 | 0 | 0 | 0 |
| S11 | very cold | below 4 | 0 | | 0 | | 0 | | S27 | | very cold | below 4 | 0.01% | 0 | 0 |
|  | Cold | 4-8 | 95.03% | | 32.27% | | 85.76% | |  |  | Cold | 4-8 | 93.25% | 30.18% | 82.05% |
|  | Cool | 8-13 | 4.97% | | 67.73% | | 14.24% | |  |  | Cool | 8-13 | 6.74% | 69.82% | 17.95% |
|  | Slightly cool | 13-18 | 0 | | 0 | | 0 | |  |  | Slightly cool | 13-18 | 0 | 0 | 0 |
| S12 | very cold | below 4 | 0 | | 0 | | 0 | | S28 | | very cold | below 4 | 0 | 0 | 0 |
|  | Cold | 4-8 | 91.77% | | 29.39% | | 80.31% | |  |  | Cold | 4-8 | 87.35% | 27.29% | 75.22% |
|  | Cool | 8-13 | 8.23% | | 70.61% | | 19.69% | |  |  | Cool | 8-13 | 12.65% | 72.71% | 24.78% |
|  | Slightly cool | 13-18 | 0 | | 0 | | 0 | |  |  | Slightly cool | 13-18 | 0 | 0 | 0 |
| S13 | very cold | below 4 | 0 | | 0 | | 0 | | S29 | | very cold | below 4 | 0 | 0 | 0 |
|  | Cold | 4-8 | 92.57% | | 29.60% | | 81.39% | |  |  | Cold | 4-8 | 89.23% | 27.52% | 76.61% |
|  | Cool | 8-13 | 7.43% | | 70.40% | | 18.61% | |  |  | Cool | 8-13 | 10.77% | 72.48% | 23.39% |
|  | Slightly cool | 13-18 | 0 | | 0 | | 0 | |  |  | Slightly cool | 13-18 | 0 | 0 | 0 |
| S14 | very cold | below 4 | 0 | | 0 | | 0 | | S30 | | very cold | below 4 | 0.01% | 0 | 0 |
|  | Cold | 4-8 | 93.39% | | 29.70% | | 82.08% | |  |  | Cold | 4-8 | 89.88% | 27.03% | 77.21% |
|  | Cool | 8-13 | 6.61% | | 70.30% | | 17.92% | |  |  | Cool | 8-13 | 10.11% | 72.97% | 22.79% |
|  | Slightly cool | 13-18 | 0 | | 0 | | 0 | |  |  | Slightly cool | 13-18 | 0 | 0 | 0 |
| S15 | very cold | below 4 | 0.01% | | 0 | | 0 | | S31 | | very cold | below 4 | 0.01% | 0 | 0 |
|  | Cold | 4-8 | 86.03% | | 27.87% | | 71.28% | |  |  | Cold | 4-8 | 78.55% | 26.38% | 63.00% |
|  | Cool | 8-13 | 13.96% | | 72.13% | | 28.72% | |  |  | Cool | 8-13 | 21.44% | 73.61% | 37.00% |
|  | Slightly cool | 13-18 | 0 | | 0 | | 0 | |  |  | Slightly cool | 13-18 | 0 | 0.01% | 0 |
| S16 | very cold | below 4 | 0 | | 0 | | 0 | | S.32 | | very cold | below 4 | 0 | 0 | 0 |
|  | Cold | 4-8 | 84.75% | | 28.27% | | 71.18% | |  |  | Cold | 4-8 | 78.46% | 27.09% | 63.69% |
|  | Cool | 8-13 | 15.25% | | 71.73% | | 28.84% | |  |  | Cool | 8-13 | 21.54% | 72.90% | 36.31% |
|  | Slightly cool | 13-18 | 0 | | 0 | | 0 | |  |  | Slightly cool | 13-18 | 0 | 0.01% | 0 |

Table A 12. The area of different PET grades during cold winter at noon time (12.00).

| No. | Slightly cool | Comfortable | Slightly warm | Warm | Hot | No. | Slightly cool | Comfortable | Slightly warm | Warm | Hot |
| --- | --- | --- | --- | --- | --- | --- | --- | --- | --- | --- | --- |
| BC | 8.80% | 18.04% | 63.40% | 9.05% | 0.71% |  |  |  |  |  |  |
| S1 | 15.58% | 31.68% | 46.62% | 5.96% | 0.16% | S17 | 14.05% | 33.21% | 46.15% | 6.44% | 0.15% |
| S2 | 15.36% | 31.11% | 47.34% | 6.02% | 0.17% | S18 | 14.08% | 32.23% | 46.94% | 6.55% | 0.20% |
| S3 | 15.43% | 30.37% | 47.83% | 6.18% | 0.19% | S19 | 14.02% | 31.38% | 47.78% | 6.62% | 0.20% |
| S4 | 17.72% | 34.79% | 41.97% | 5.36% | 0.16% | S20 | 16.47% | 36.08% | 41.69% | 5.63% | 0.13% |
| S5 | 17.25% | 34.53% | 42.61% | 5.45% | 0.16% | S21 | 15.94% | 35.67% | 42.50% | 5.74% | 0.15% |
| S6 | 17.04% | 34.17% | 43.08% | 5.54% | 0.17% | S22 | 15.88% | 35.11% | 43.01% | 5.84% | 0.16% |
| S7 | 19.12% | 40.55% | 35.50% | 4.80% | 0.03% | S23 | 16.45% | 43.25% | 34.95% | 5.31% | 0.04% |
| S8 | 24.75% | 38.21% | 32.73% | 4.29% | 0.02% | S24 | 24.06% | 39.96% | 31.57% | 4.38% | 0.03% |
| S9 | 23.63% | 31.00% | 40.61% | 4.64% | 0.12% | S25 | 22.91% | 31.73% | 39.97% | 5.27% | 0.12% |
| S10 | 23.10% | 30.83% | 41.30% | 4.66% | 0.11% | S26 | 22.29% | 31.52% | 40.84% | 5.23% | 0.12% |
| S11 | 22.45% | 30.30% | 42.27% | 4.87% | 0.11% | S27 | 22.07% | 31.04% | 41.51% | 5.26% | 0.12% |
| S12 | 27.28% | 32.60% | 35.70% | 4.36% | 0.06% | S28 | 27.46% | 33.51% | 34.12% | 4.84% | 0.07% |
| S13 | 26.11% | 32.88% | 36.50% | 4.44% | 0.07% | S29 | 26.24% | 33.93% | 34.99% | 4.76% | 0.08% |
| S14 | 25.77% | 32.73% | 37.05% | 4.36% | 0.09% | S30 | 26.36% | 32.99% | 35.79% | 4.79% | 0.07% |
| S15 | 30.99% | 35.64% | 29.68% | 3.69% | 0.00% | S31 | 29.19% | 37.34% | 29.63% | 3.84% | 0.00% |
| S16 | 29.79% | 35.91% | 30.51% | 3.79% | 0.00% | S32 | 27.45% | 38.67% | 30.02% | 3.86% | 0.00% |

**Appendix B**


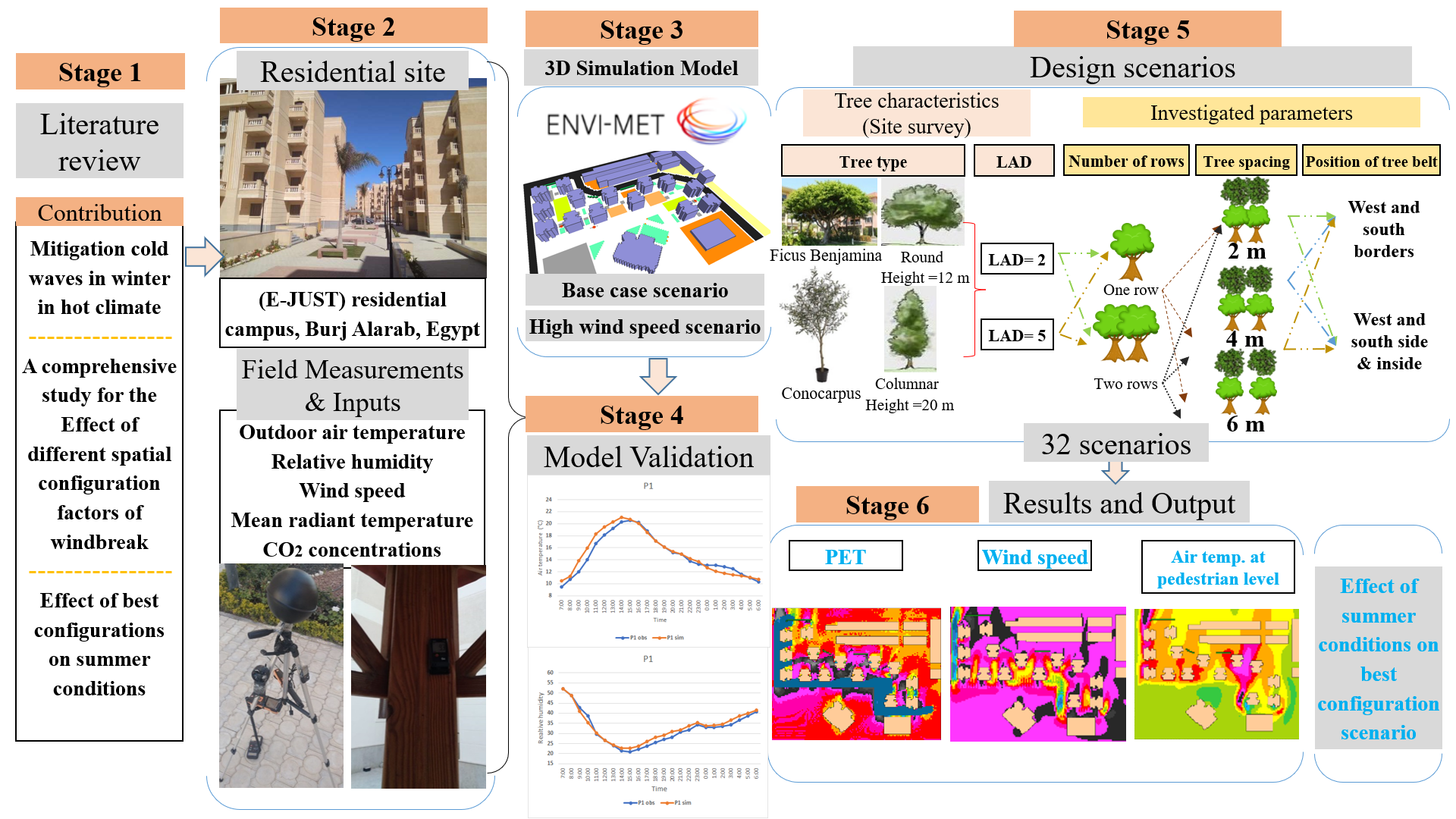


Figure B 1. Different stages of this study.

| 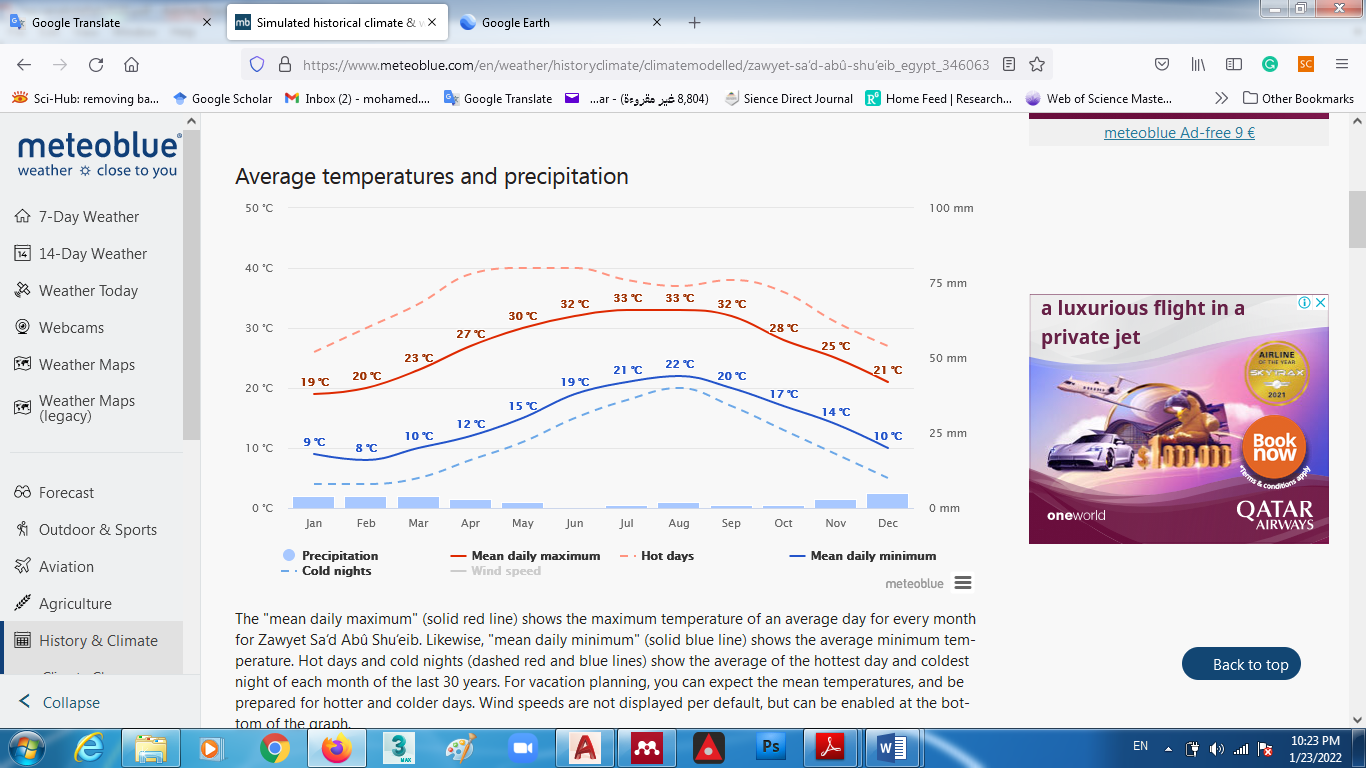 | | | | | | | 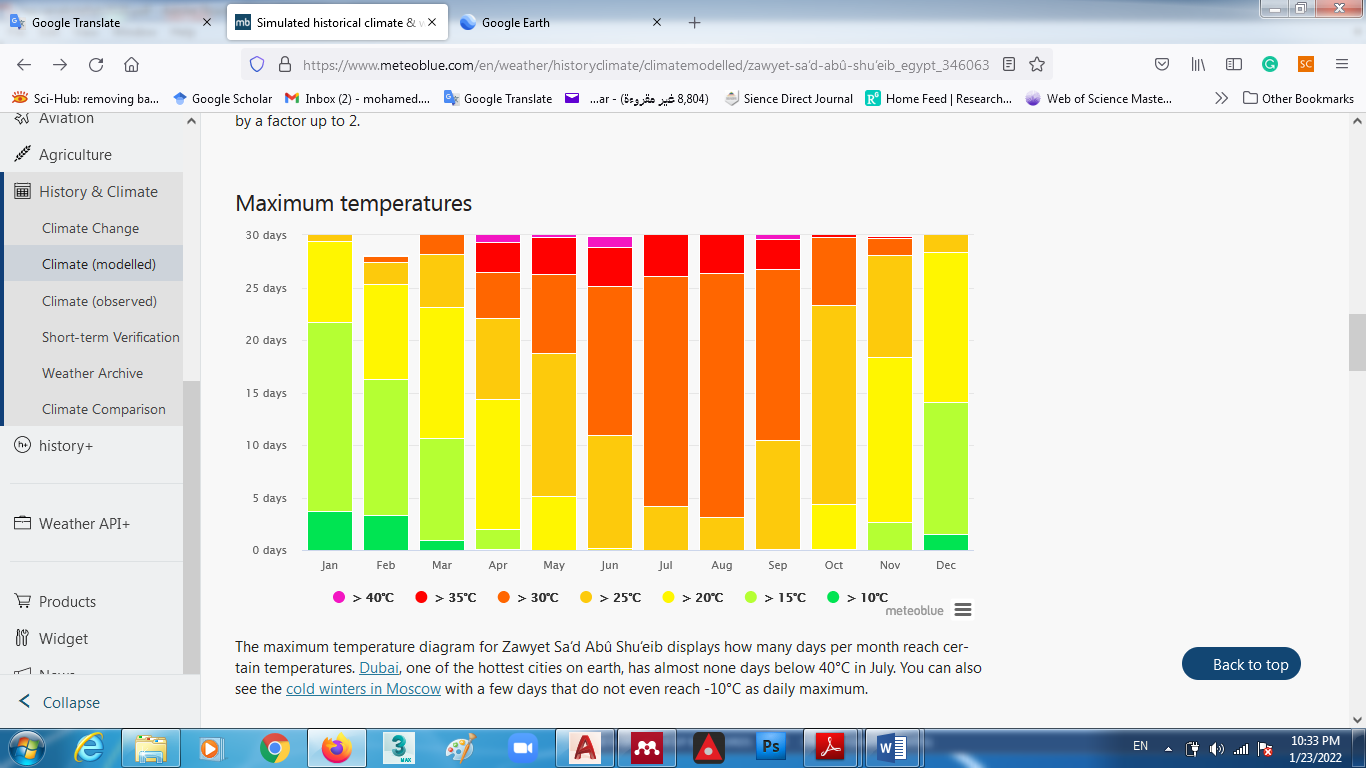 | | | | | |  |  |
| --- | --- | --- | --- | --- | --- | --- | --- | --- | --- | --- | --- | --- | --- | --- |
| 1. Average air temperatures. | | | | | | | 1. Number of cold and hot days. | | | | | |  |  |
| 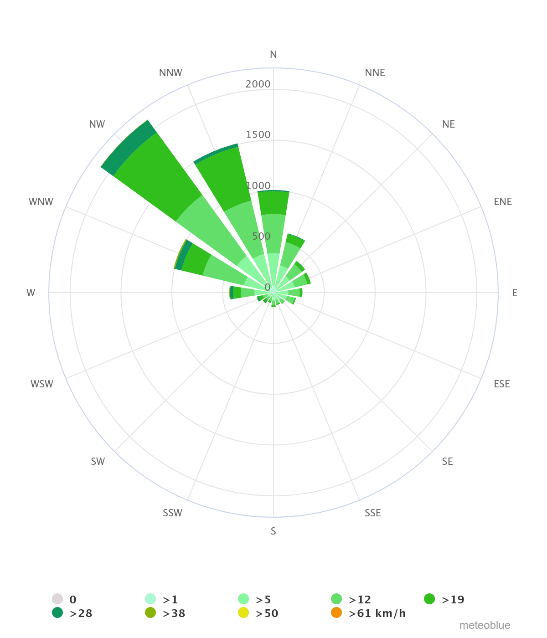 | | | | | 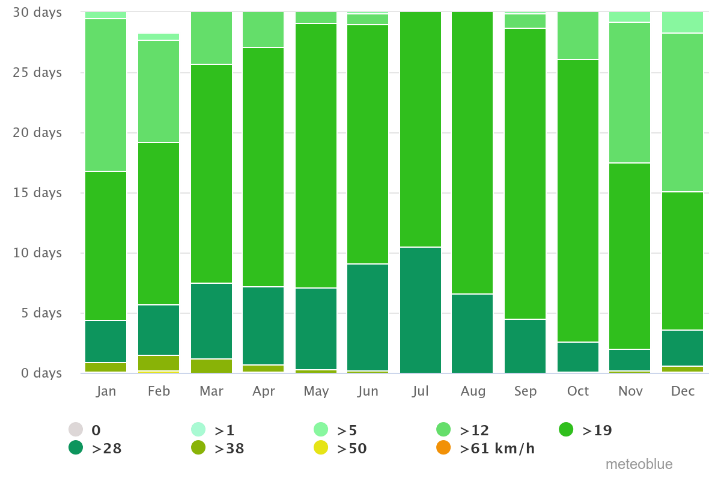 | | | | | | | | |  |
| 1. The dominant wind direction | | | | | | | 1. Number of days for every wind speed category | | | | | |  |  |
| Figure B 2. Different climate conditions during all year in NBAC (www.meteoblue.com, 2022). | | | | | | | | | | | | |  |  |
| 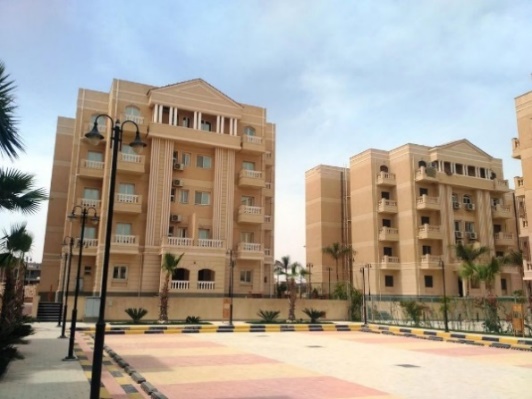 | | | | | | | 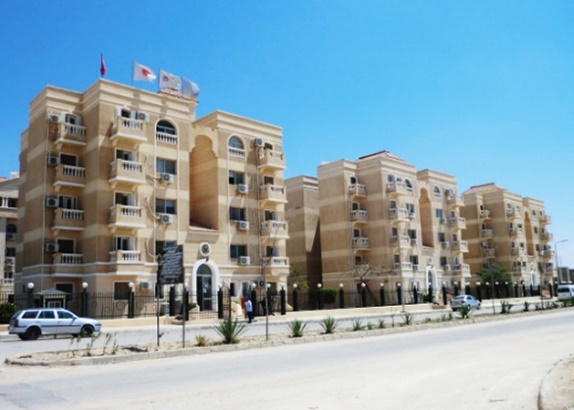 | | | | |  |  |  |
| 1. Back facades | | | | | | | 1. Entrance facades | | | | |  |  |  |
| Figure B 3. buildings’ exterior finishing of the campus. | | | | | | | | | | | |  |  |  |
| 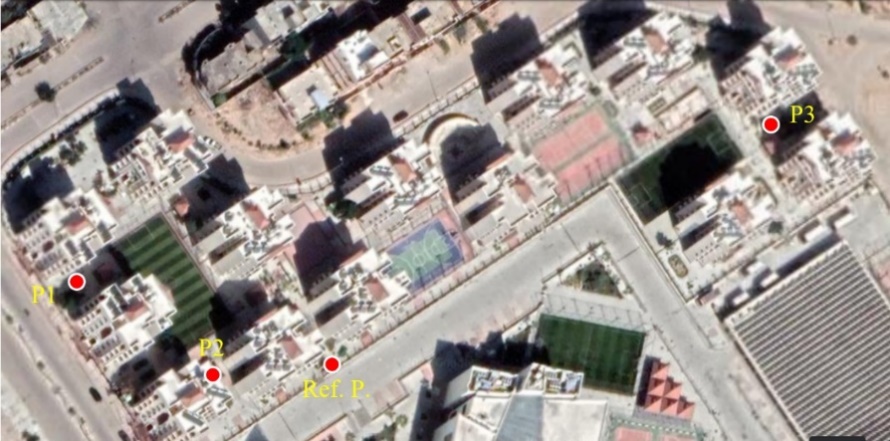  **Ref. P.**  **P3**  **P2**  **P1** | | | | | | | | | | | | |  |  |
| 1. Position of different measurement points inside the campus. | | | | | | | | | | | | |  |  |
| 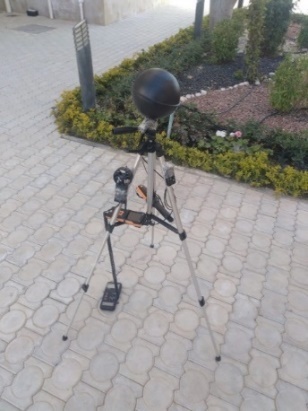 | | | | 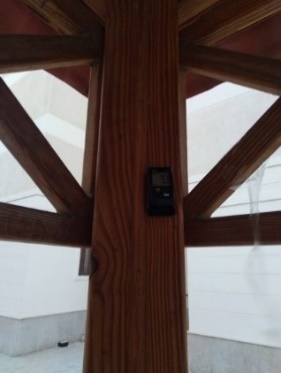 | | | | | | | | |  |  |
| 1. The portable measurements tools | | | | 1. Testo 174h data logger in measurement points | | | | | | | | |  |  |
| Figure B 4. The measurement devices and the location of measurement points. | | | | | | | | | | | | |  |  |
| 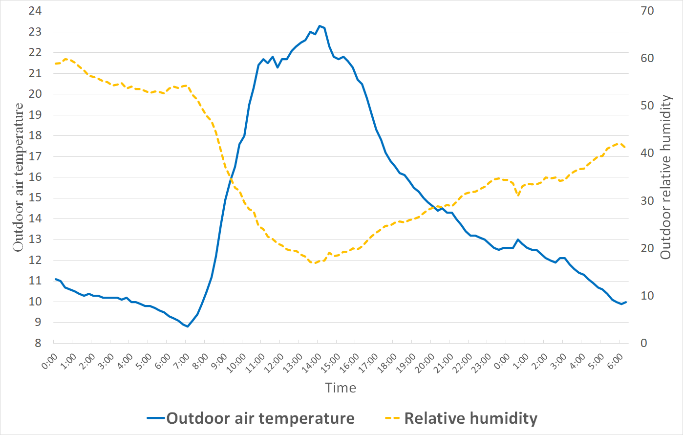 | | | | | | | 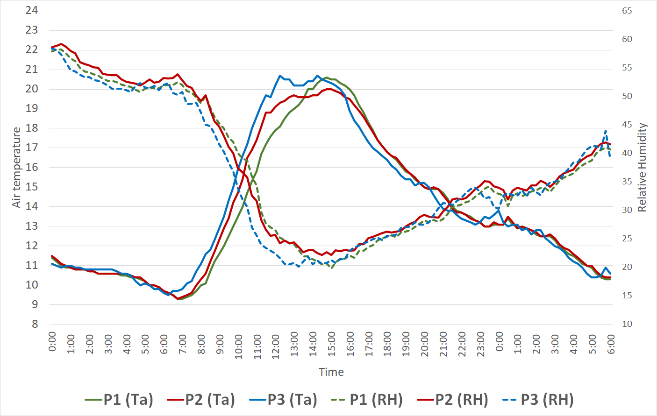 | | | | | |  |  |
| 1. Climate conditions at reference point. | | | | | | 1. AT and RH. | | | | | | |  |  |
| 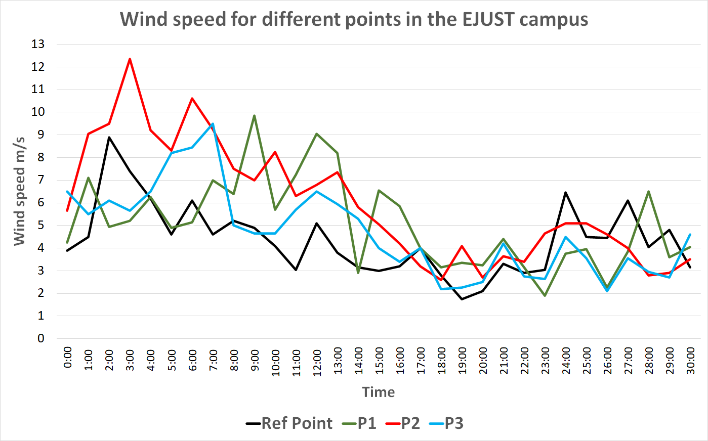 | | | | | | | 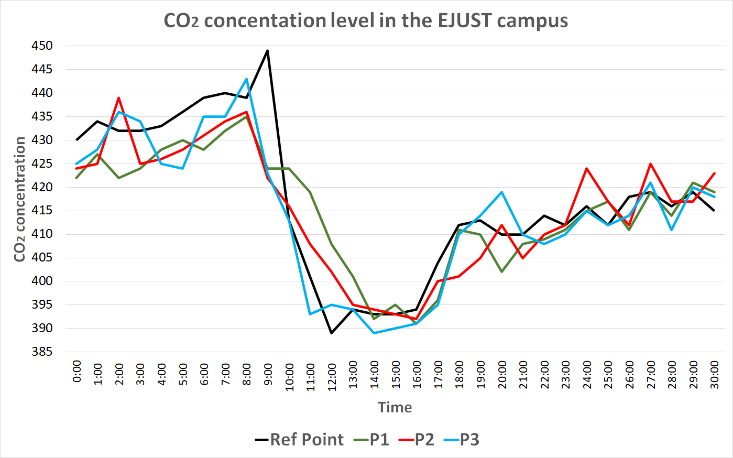 | | | | | |  |  |
| 1. Average wind speed | | | 1. Concentration of CO_2_ (ppm). | | | | | | | | | | | |
| Figure B 5. Field measurements for different microclimate parameters at different points during the cold winter day. | | | | | | | | | | | | |  |  |
| 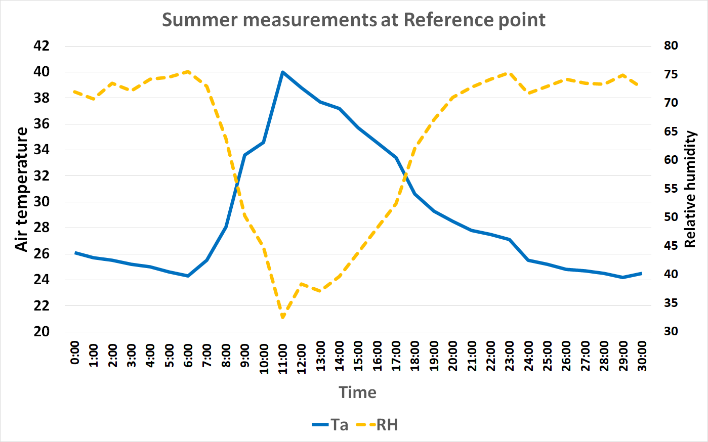 | | | | | | | | | | | | |  |  |
| Figure B 6. The summer measurements during all day at the reference point. | | | | | | | | | | | | |  |  |
| 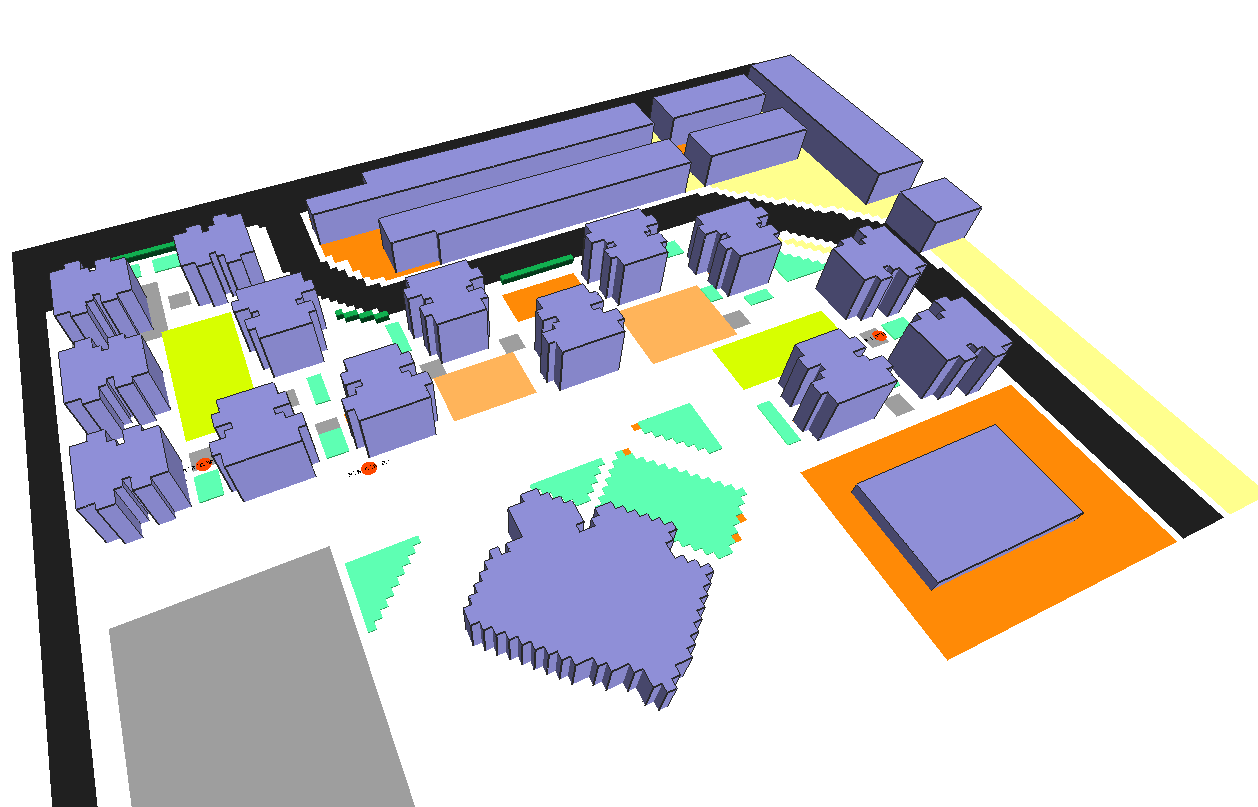 | | | | | | | | | | | | |  |  |
| Figure B 7. The ENVI-met model for E-JUST residential campus. | | | | | | | | | | | | |  |  |
| 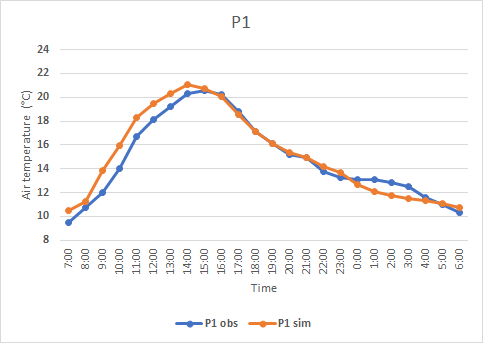 | | 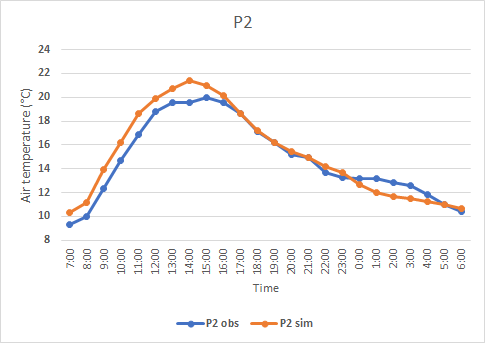 | | | | | | | 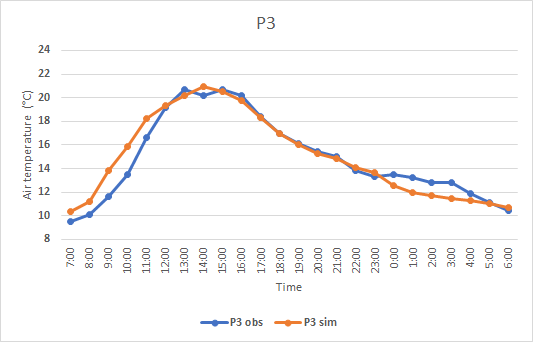 | | | | | |
| 1. AT (Point 1) | | 1. AT (Point 2) | | | | | | | 1. AT (Point 3) | | | | | |
| 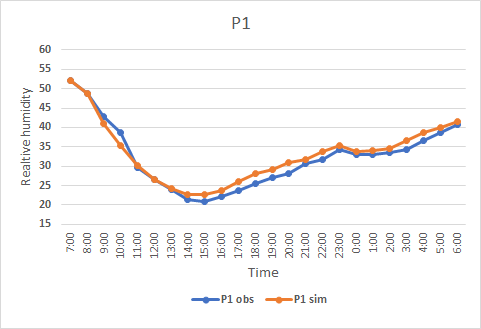 | | 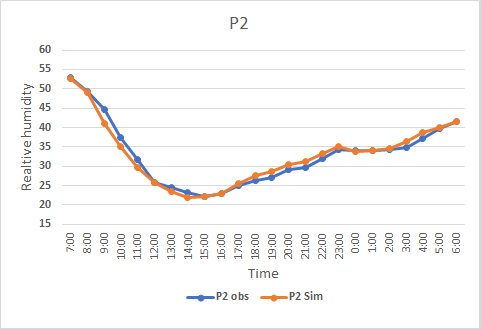 | | | | | | | 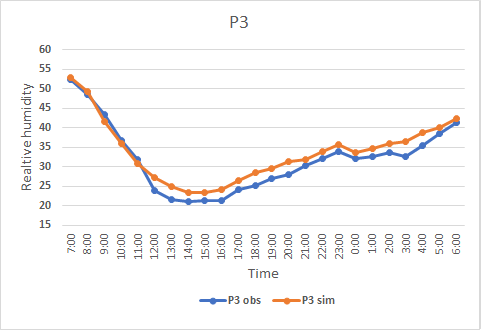 | | | | | |
| 1. RH (Point 1) | | 1. RH (Point 2) | | | | | | | 1. RH (Point 3) | | | | | |
| 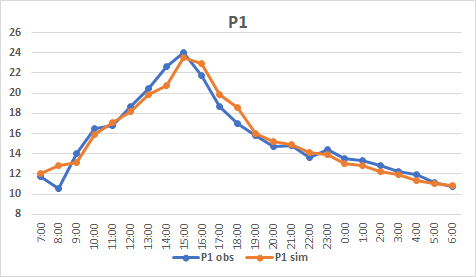 | | 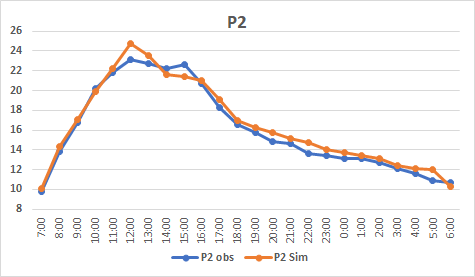 | | | | | | | 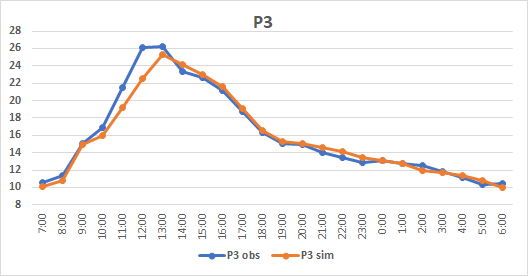 | | | | | |
| 1. MRT (Point 1) | | 1. MRT (Point 2) | | | | | | | 1. MRT (Point 3) | | | | | |
| Figure B 8. Validation for hourly AT, RH and MRT (measured vs simulated) at different points. | | | | | | | | | | | | | | |
| 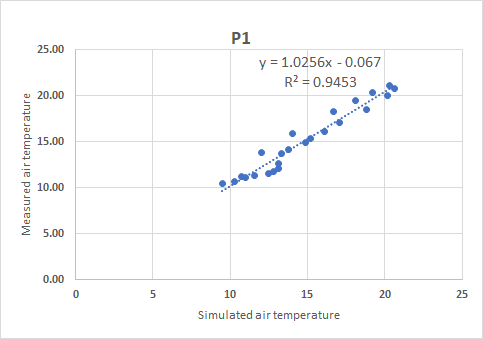 | | 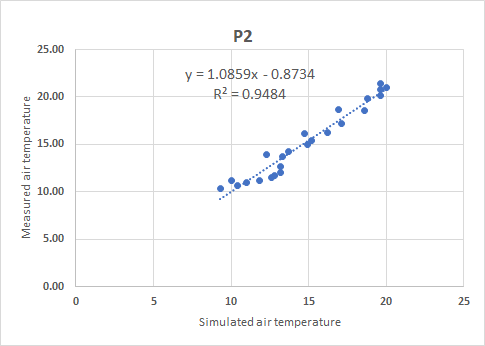 | | | | | | | 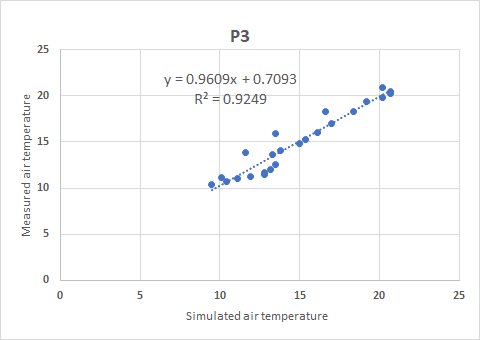 | | | | | |
| 1. AT (Point 1) | | 1. AT (Point 2) | | | | | | | 1. AT (Point 3) | | | | | |
| 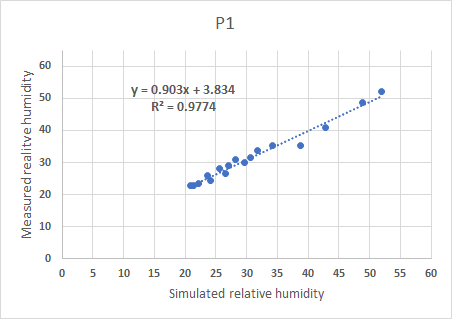 | | 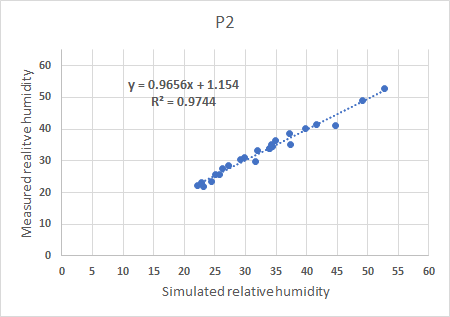 | | | | | | | 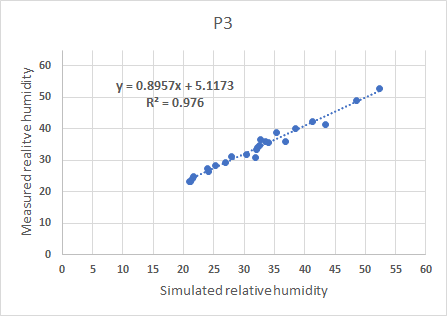 | | | | | |
| 1. RH (Point 1) | | 1. RH (Point 2) | | | | | | | 1. RH (Point 3) | | | | | |
| 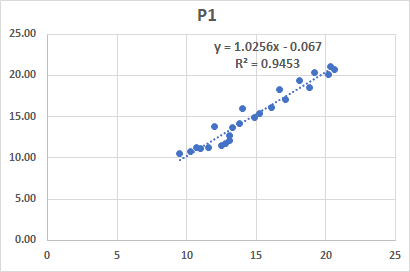 | | 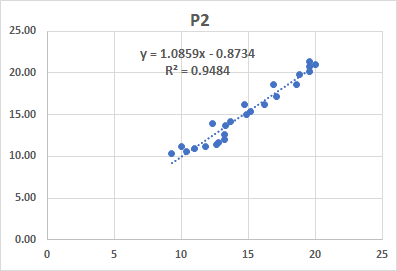 | | | | | | | 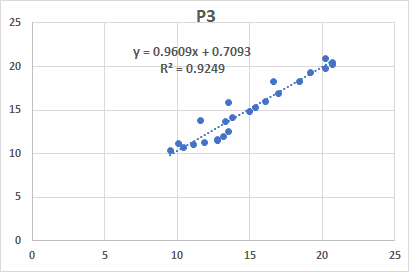 | | | | | |
| 1. MRT (Point 1) | | 1. MRT (Point 2) | | | | | | | 1. MRT (Point 3) | | | | | |
| Figure B 9. Correlations between measured and simulated AT, RH and MRT at different points. | | | | | | | | | | | | | | |
| 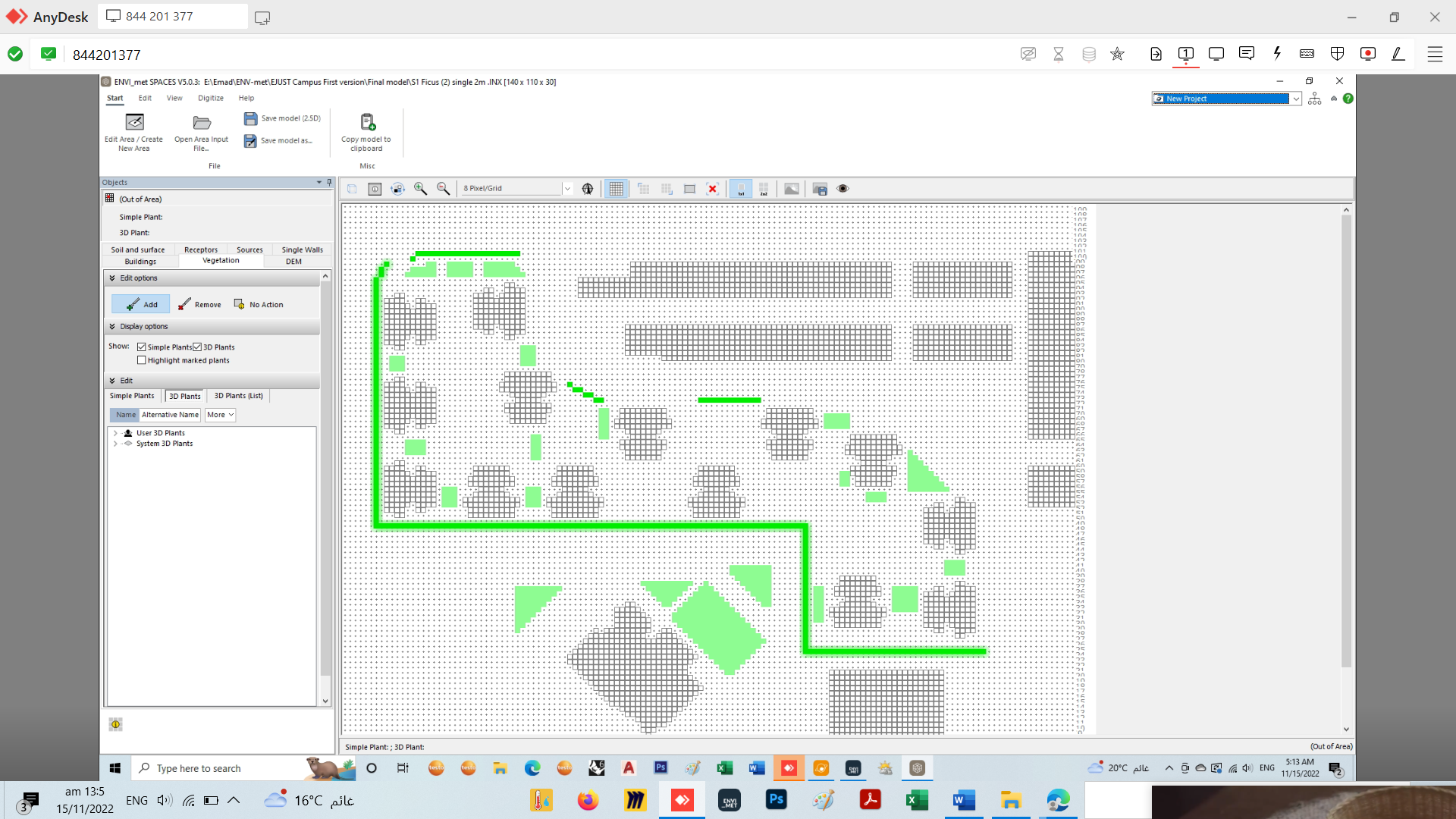 | 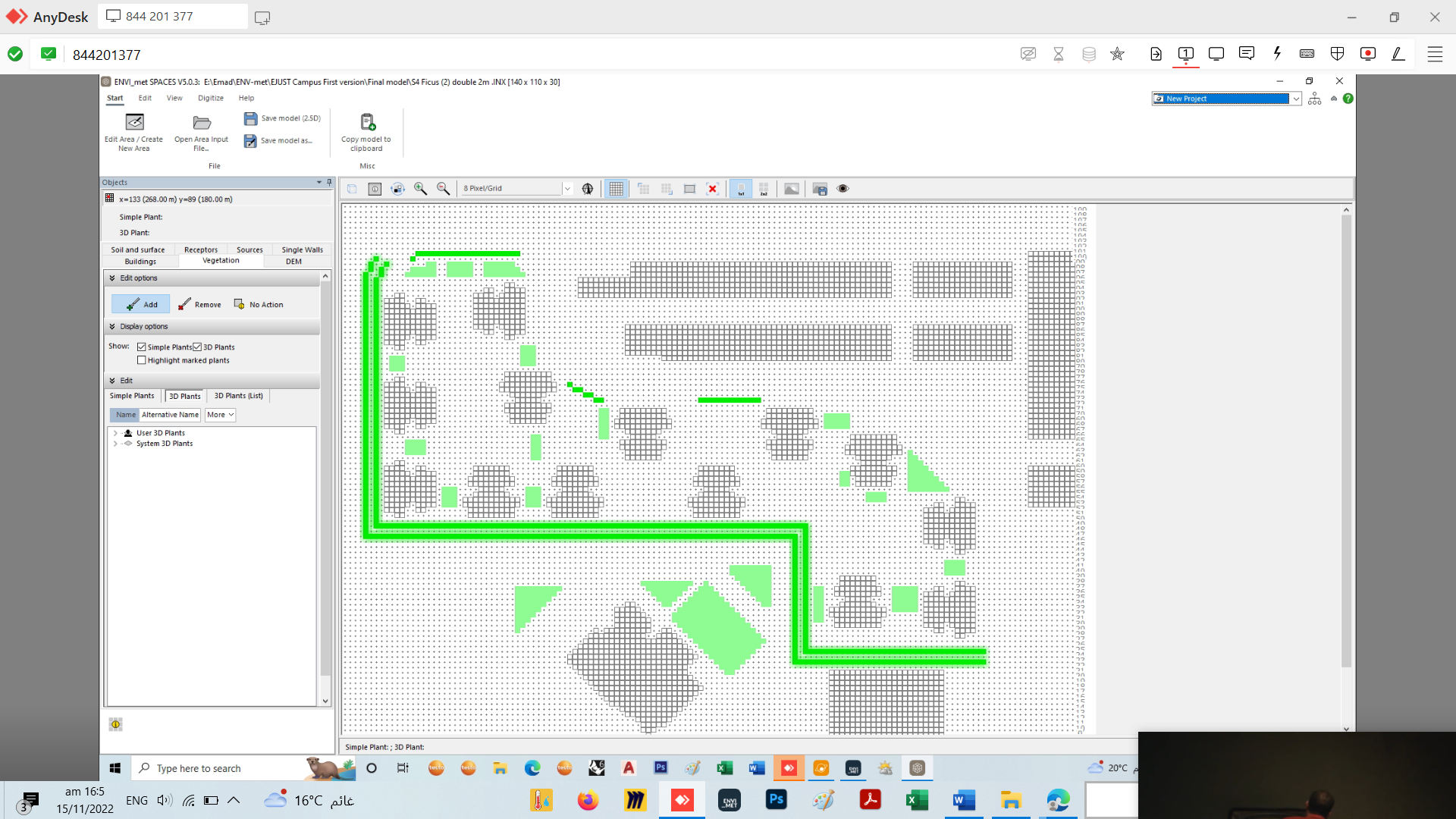 | | | | | | | 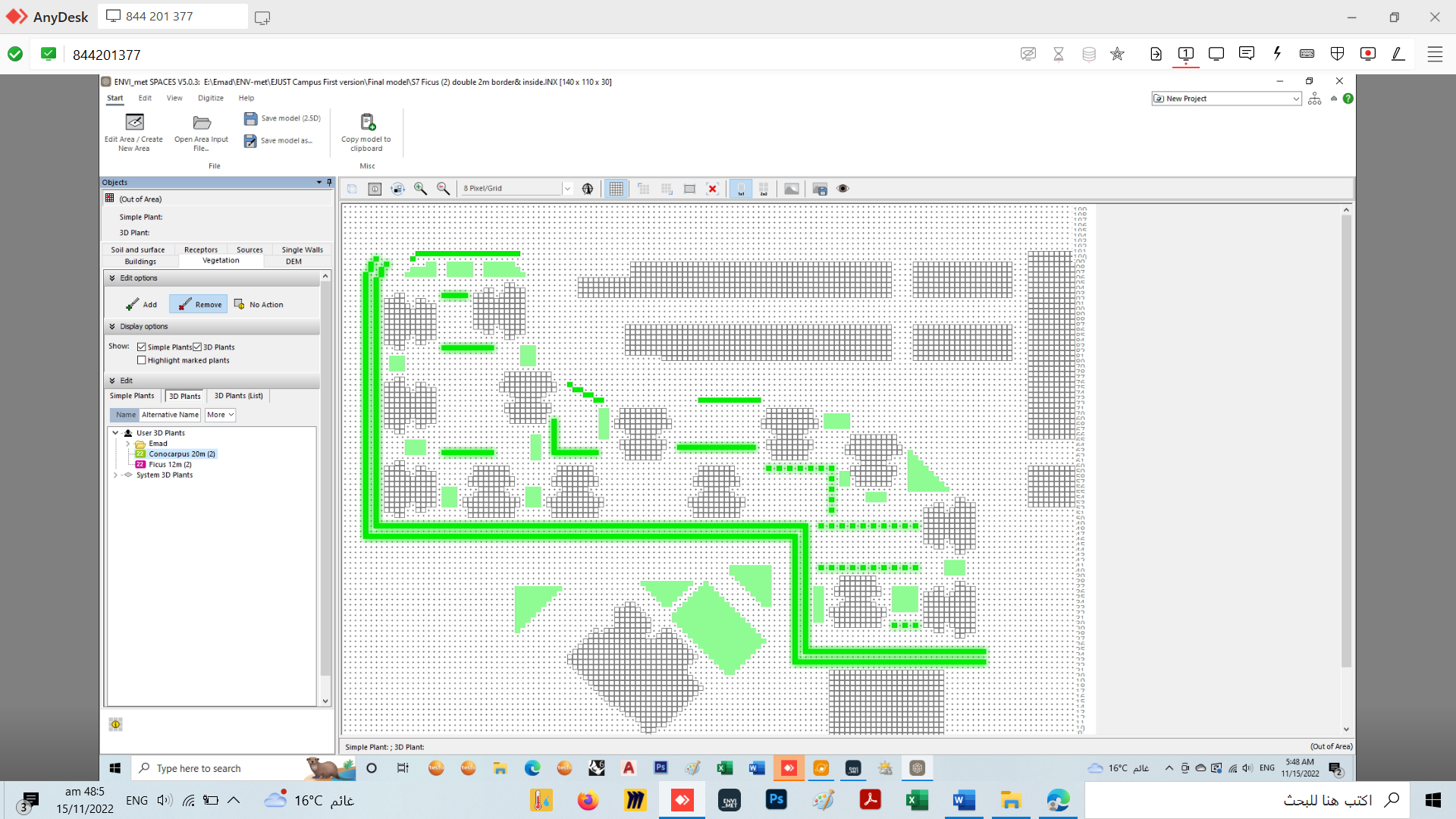 | | | | |  |  |
| 1. Single row. | 1. Double row. | | | | | | | 1. inside and site borders. | | | | |  |  |
| Figure B 10. Tree distribution in different scenarios. | | | | | | | | | | | | |  |  |
| 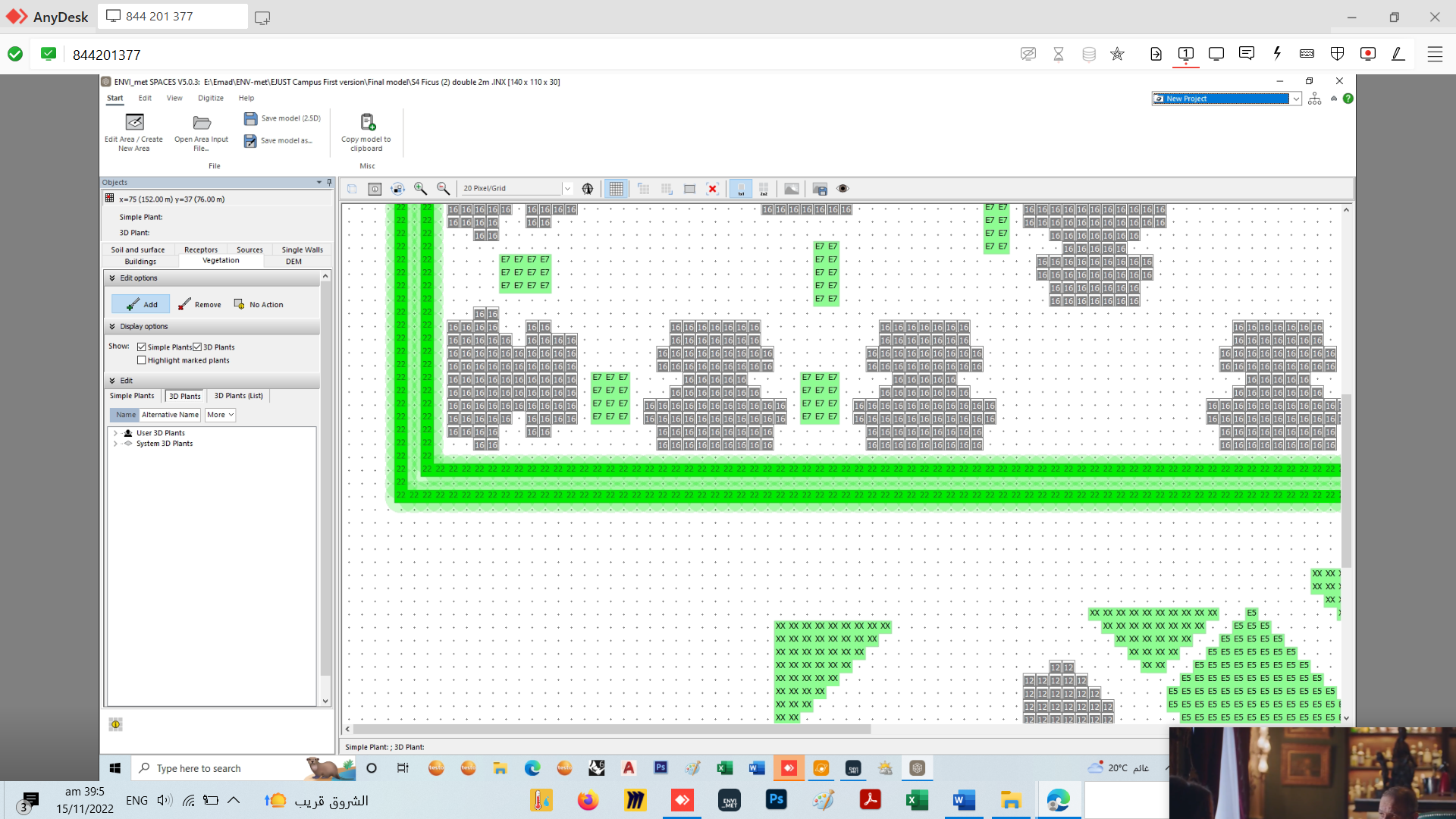 | | 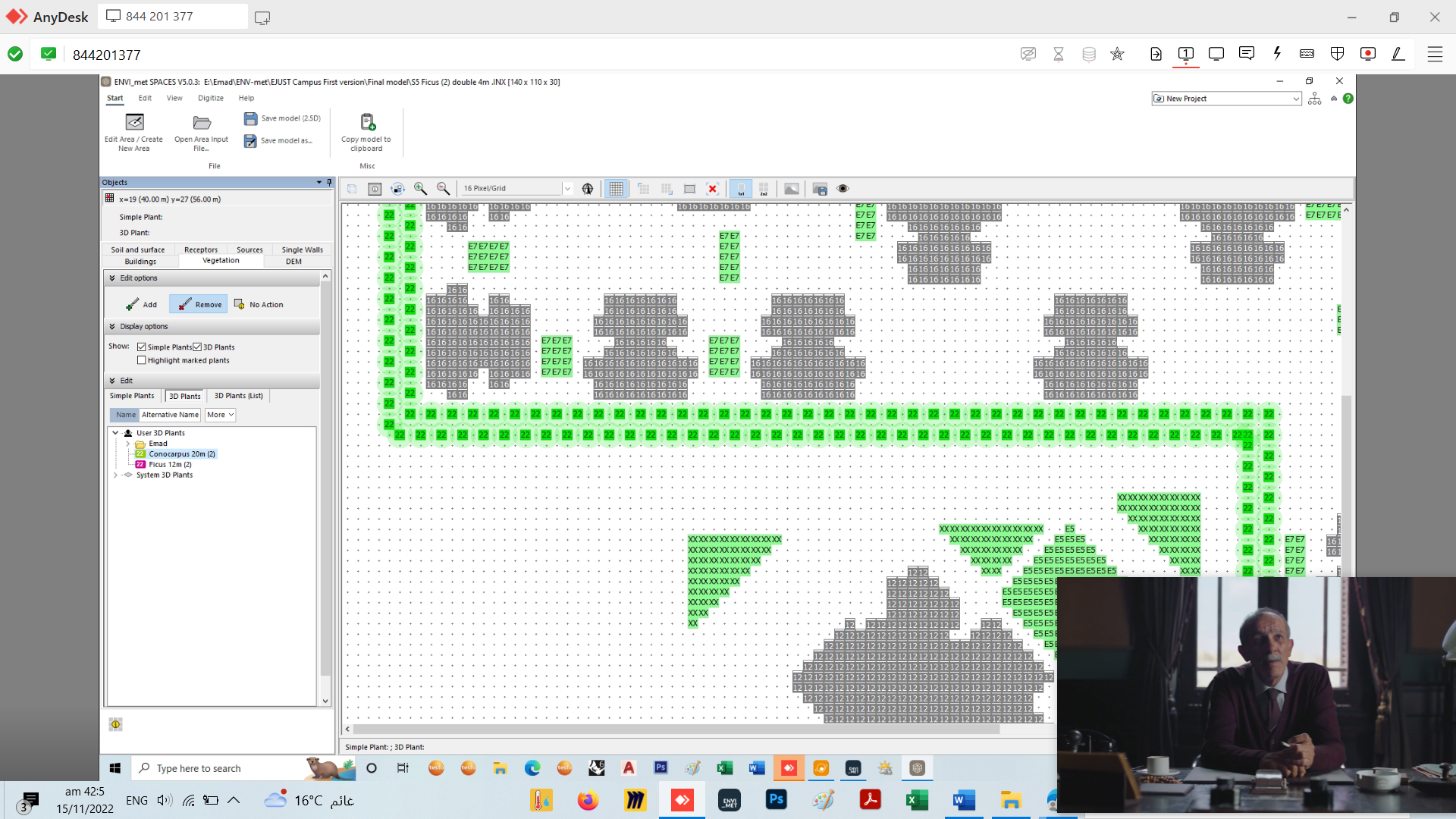 | | | | | | | 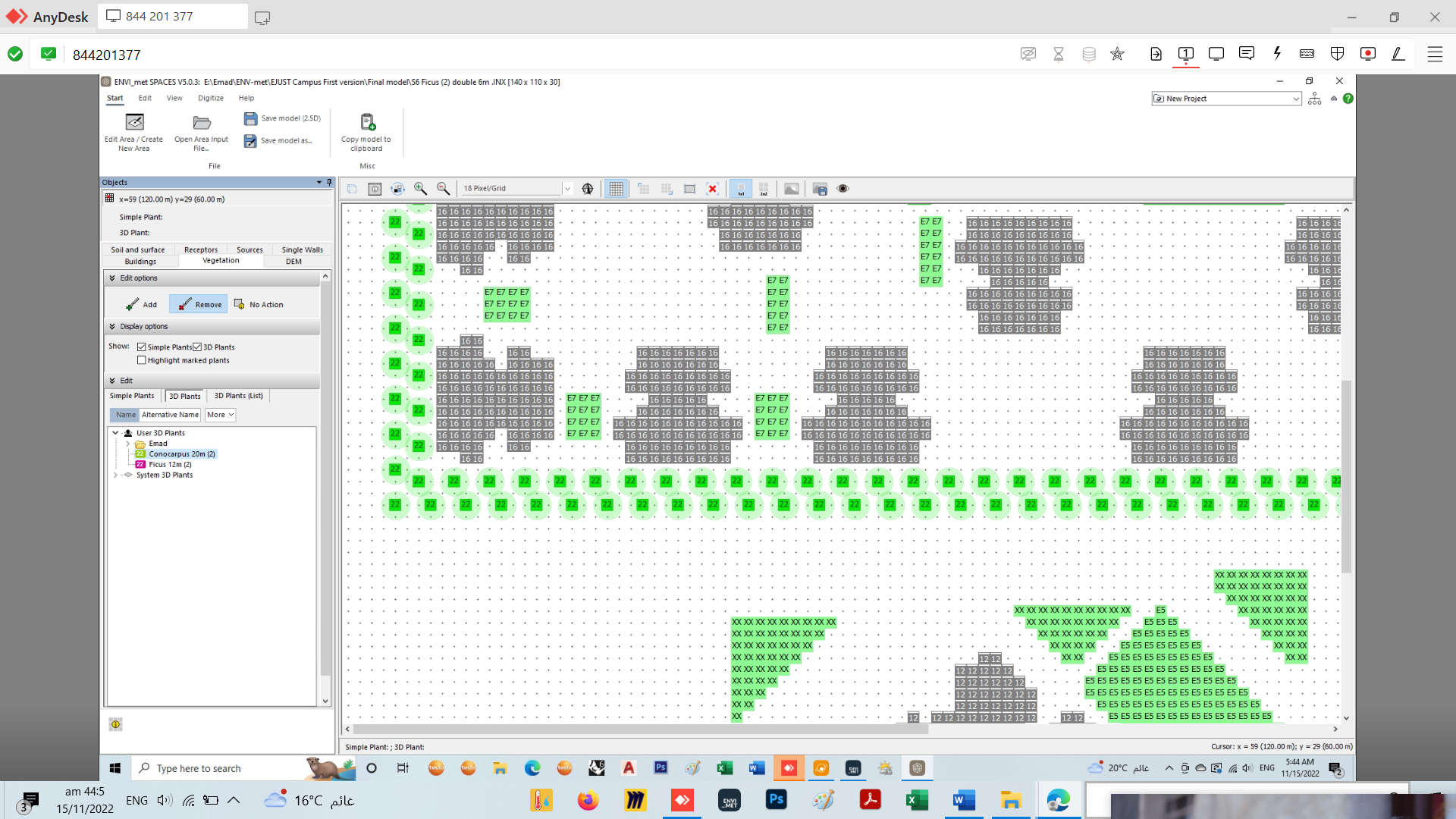 | | | |  |  |
| 1. 2m. | | 1. 4m. | | | | | | | 1. 6m. | | | |  |  |
| Figure B 11. Different tree spacing in double rows scenarios. | | | | | | | | | | | | |  |  |
| 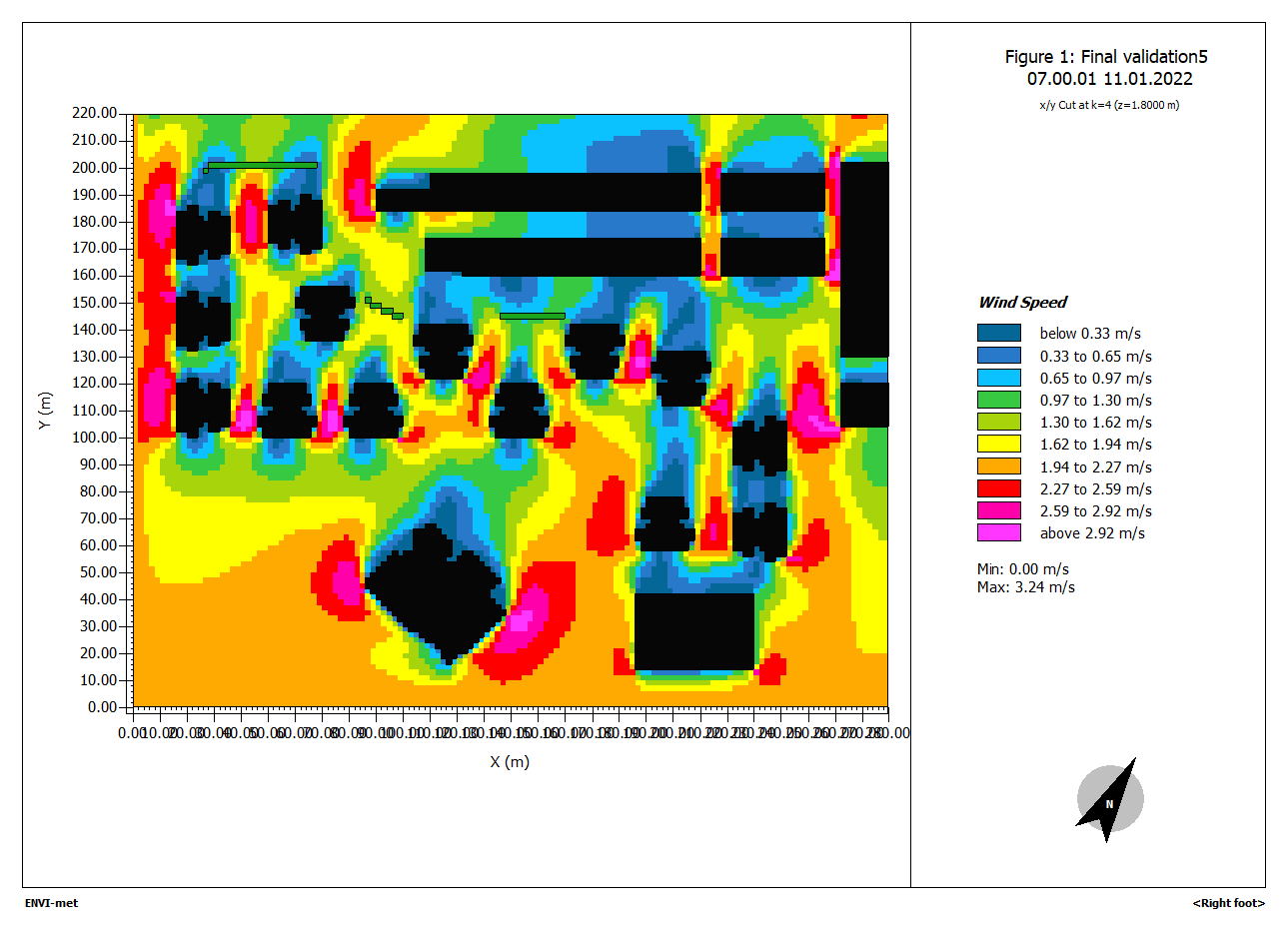  **4**  **3**  **2**  **1** | | | | | | | | | | | | |  |  |
| Figure B 12. Different positions to monitor the effect of windbreak on windspeed. | | | | | | | | | | | | |  |  |
| 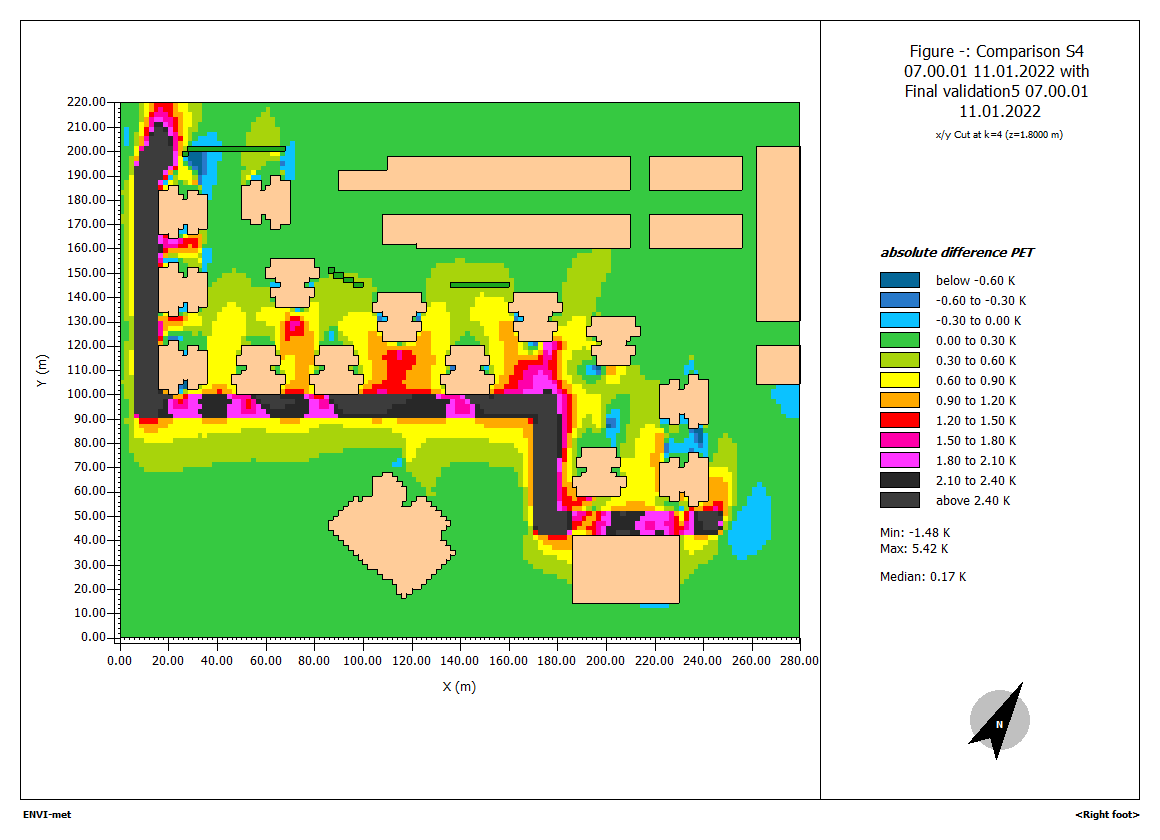 | | | 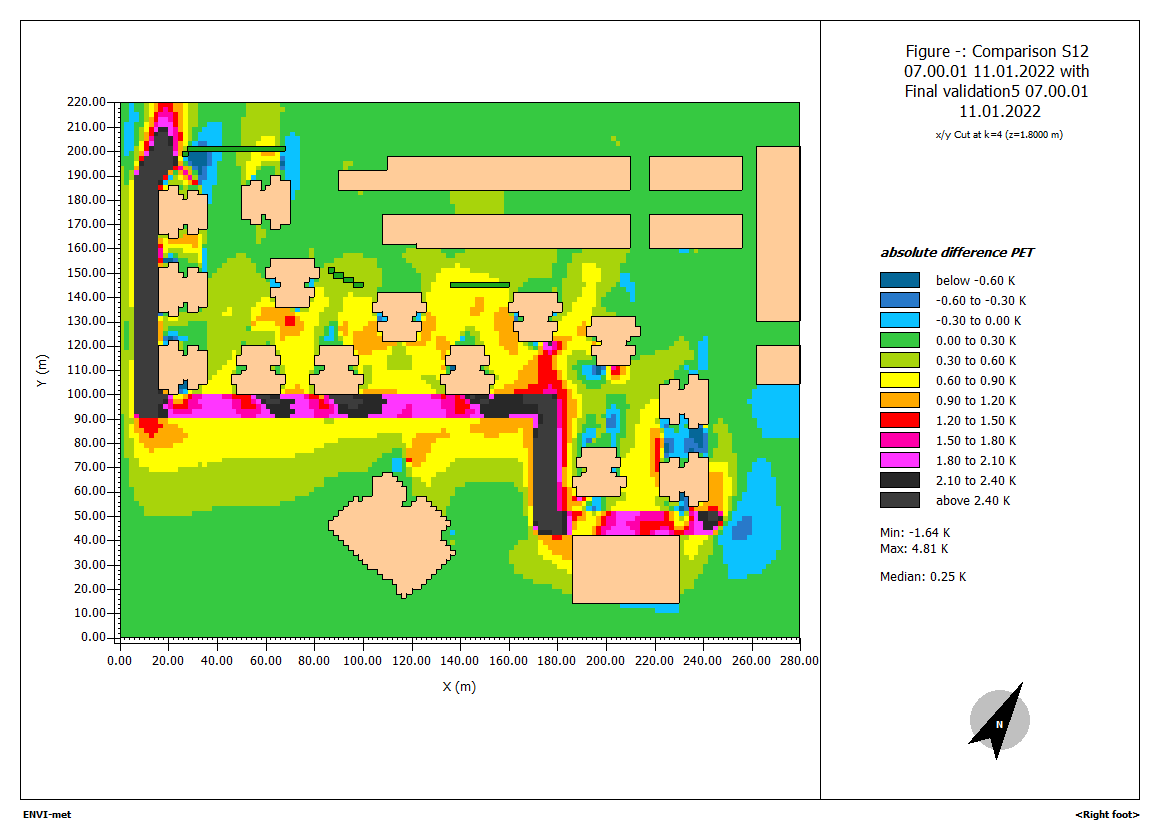 | | | | | | | 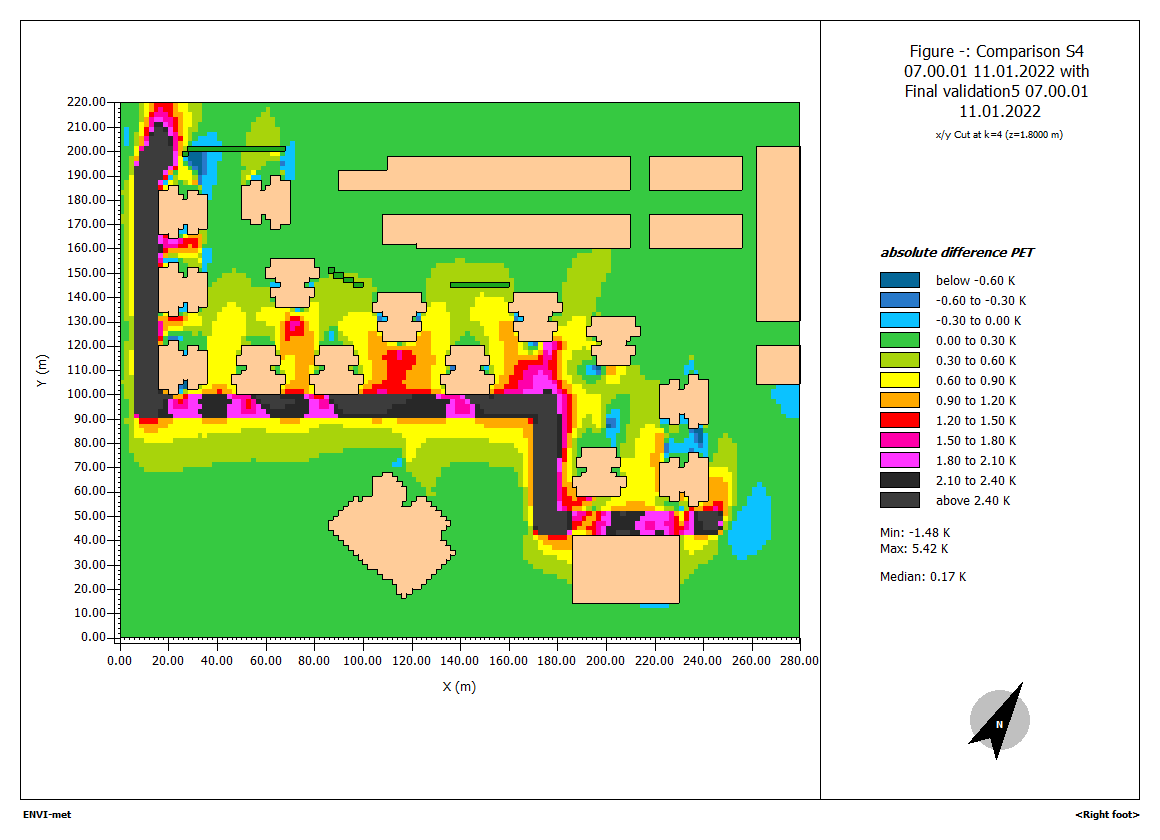 | | |  |  |
| 1. S4 (spacing 2m) (Ficus LAD=2) | | | 1. S11 (spacing 2m) (Cono. LAD=2) | | | | | | | Scale | | |  |  |
| 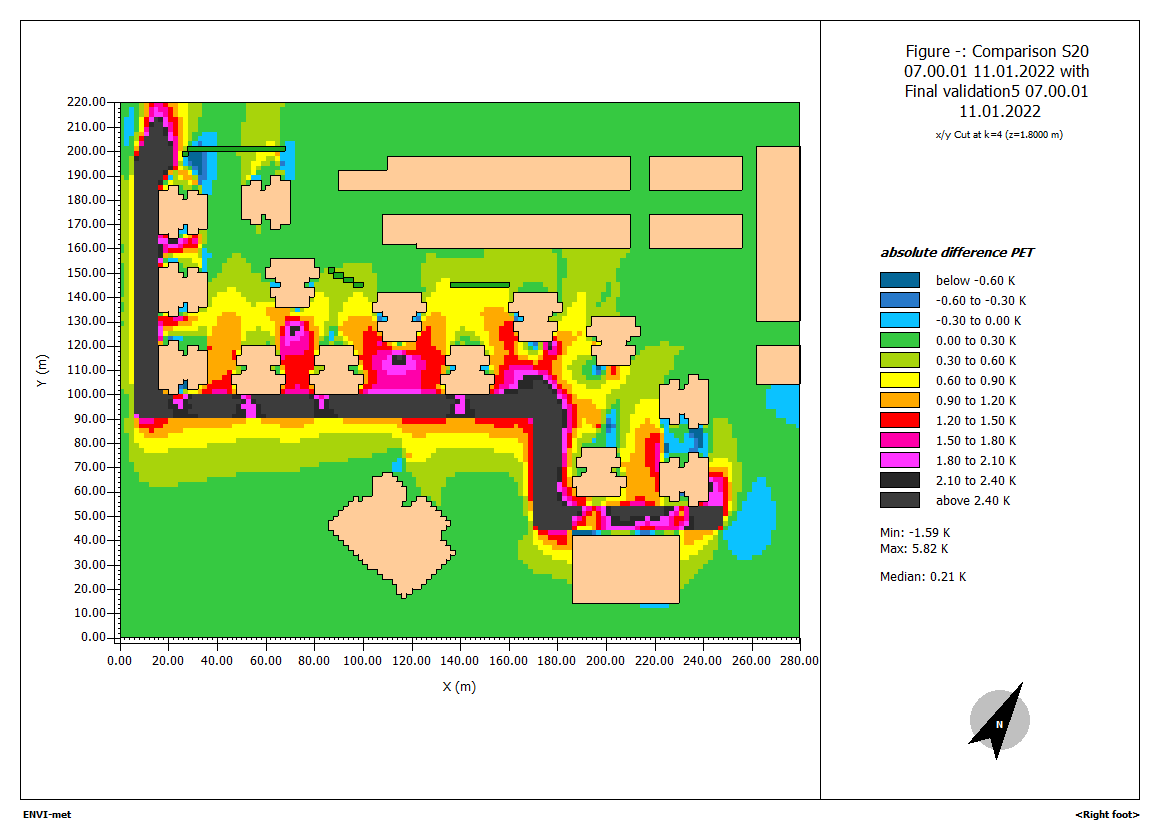 | | | 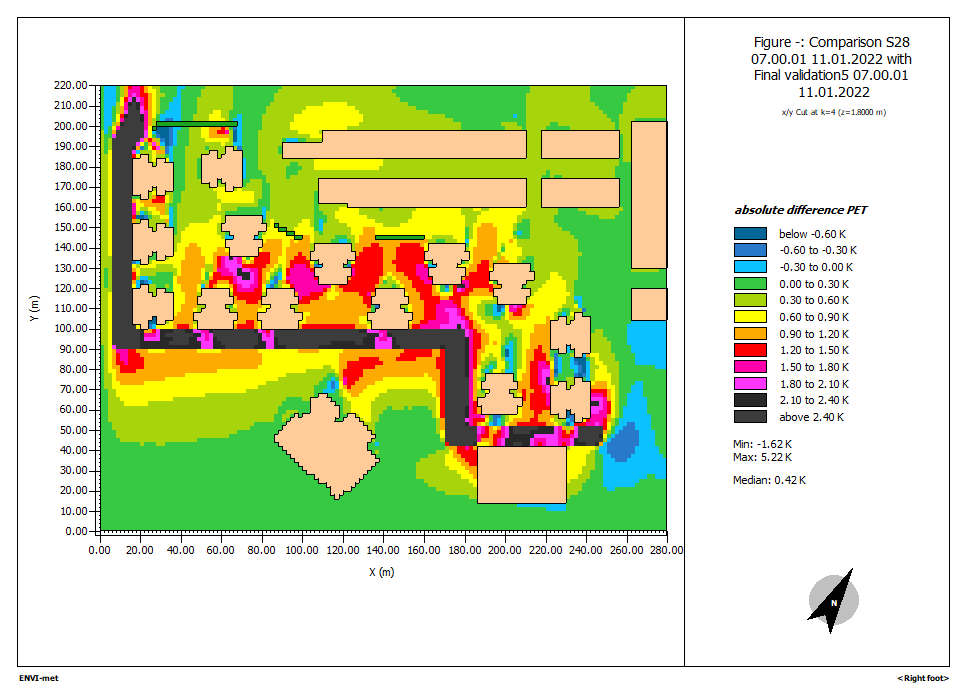 | | | | | | | 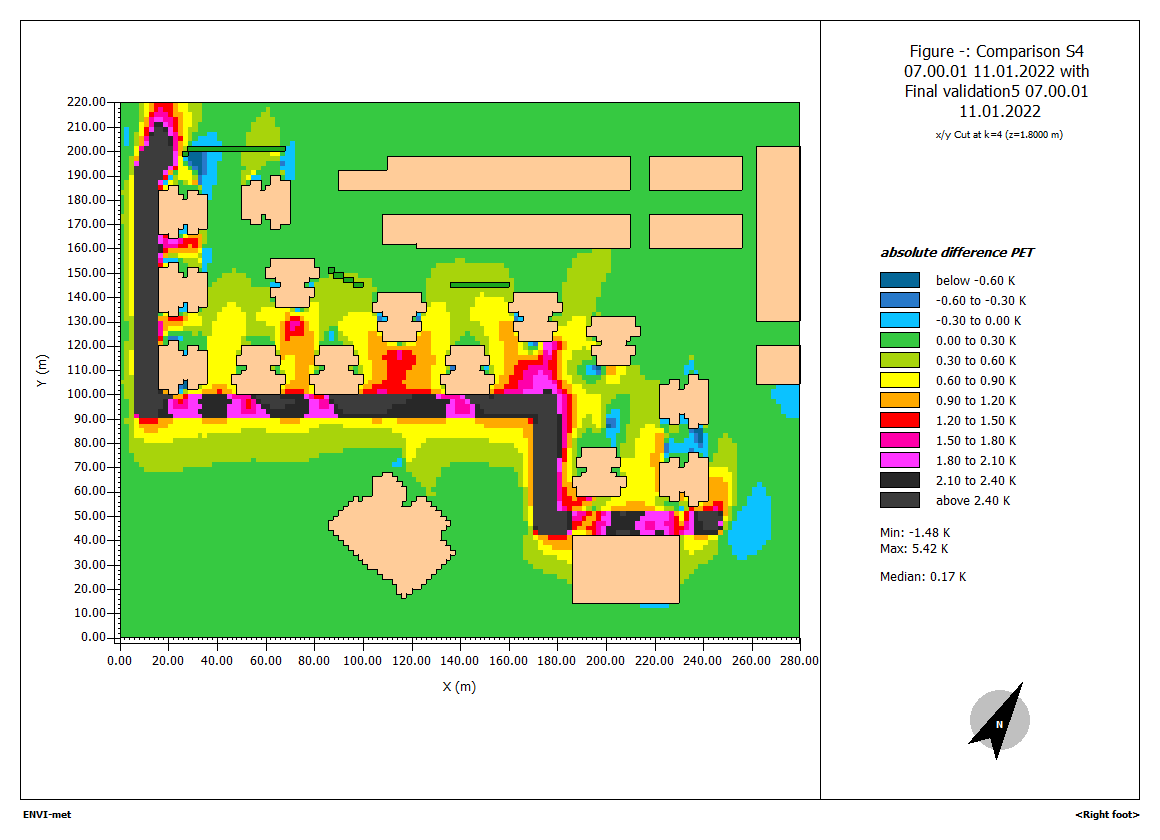 | | |  |  |
| 1. S20 (spacing 2m) (Ficus LAD=5) | | | 1. S28 (spacing 2m) (Cono. LAD=5) | | | | | | | Scale | | |  |  |
| Figure B 13. PET differences between different scenarios and BCS at 7.00. | | | | | | | | | | | | |  |  |
| 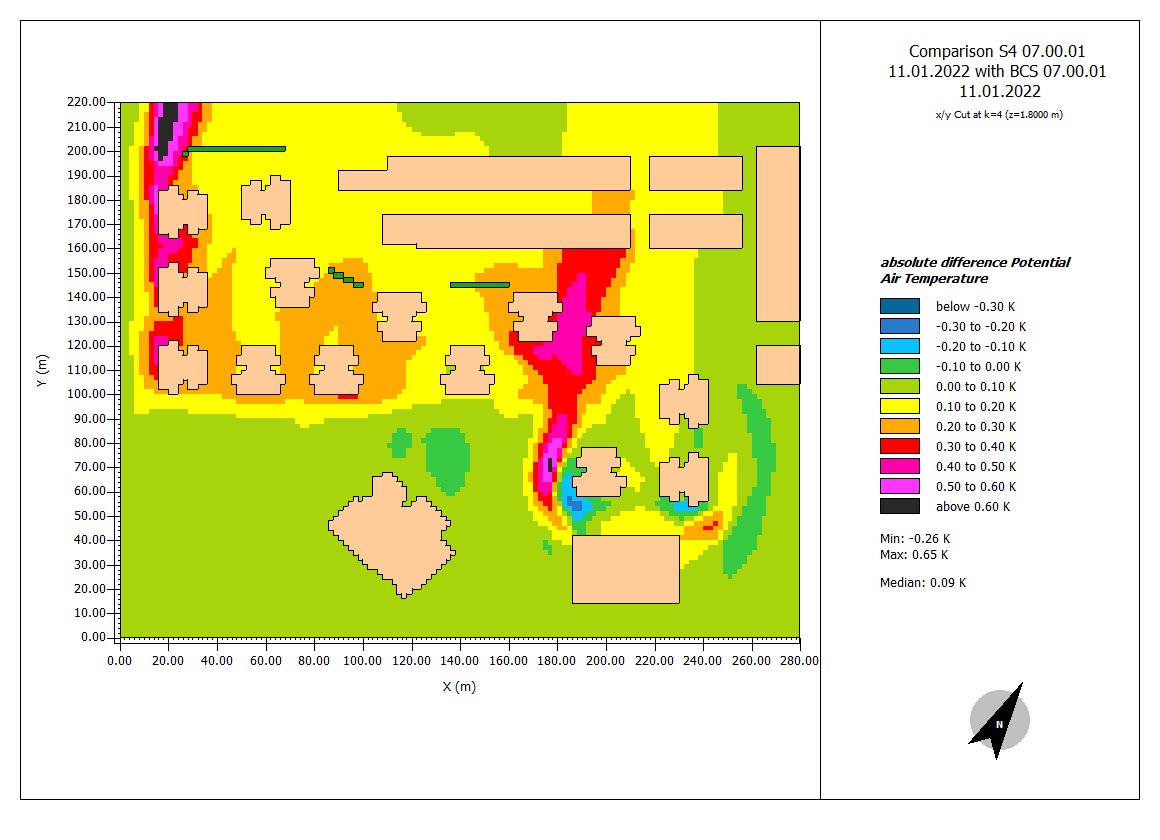 | | | 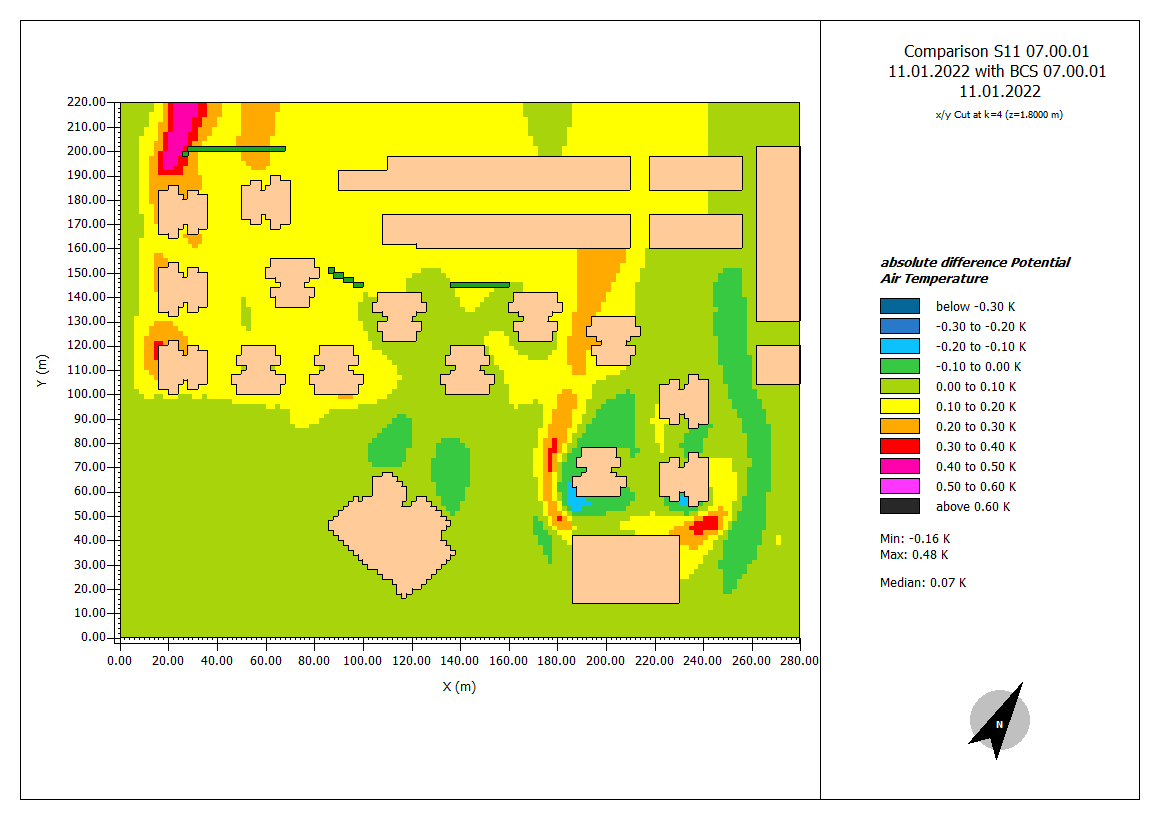 | | | | | | | | 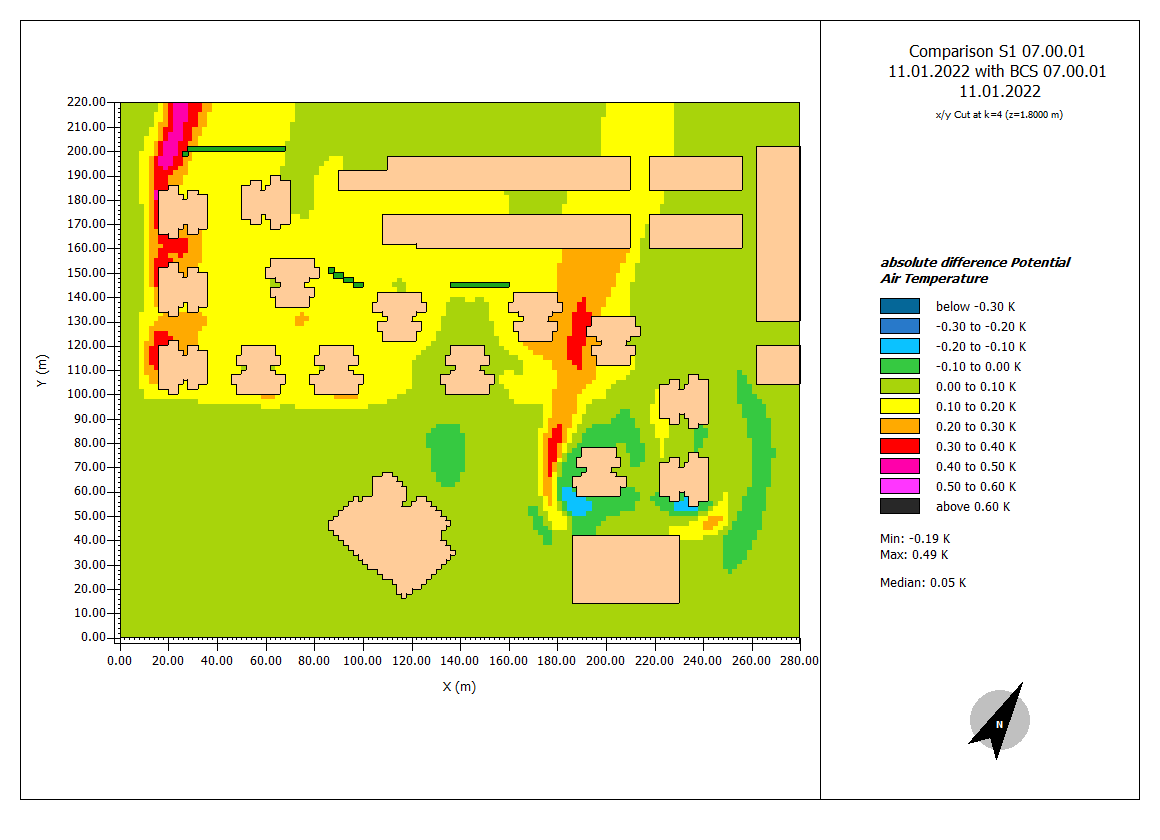 | |  |  |
| 1. S4 (spacing 2m) (Ficus LAD=2) | | | 1. S11 (spacing 2m) (Cono. LAD=2) | | | | | | | | Scale | |  |  |
| 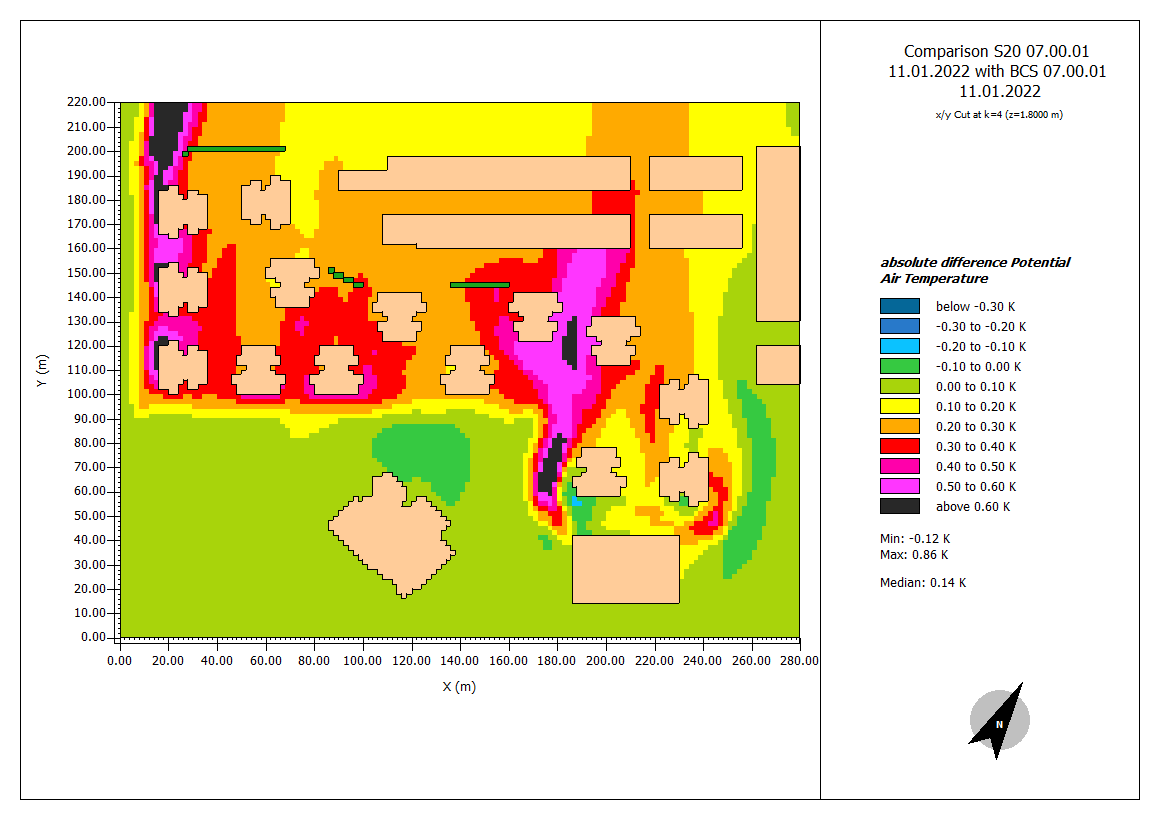 | | | 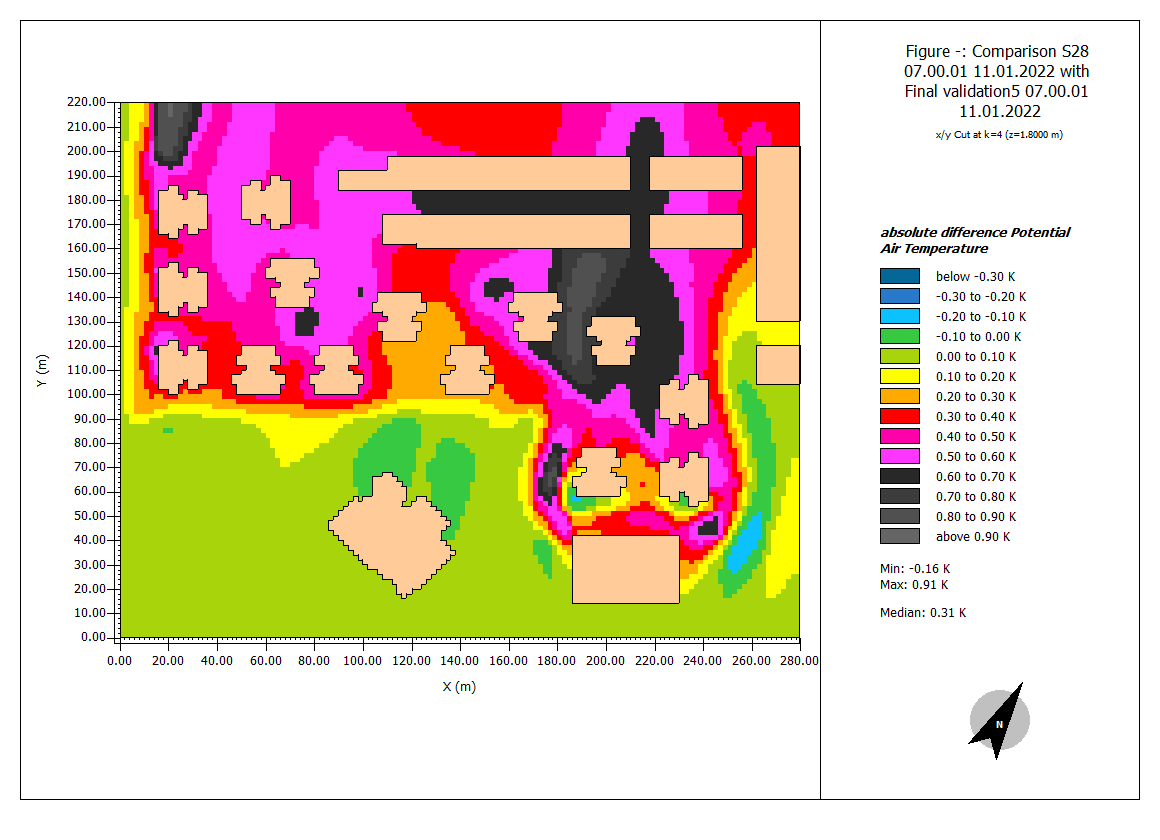 | | | | | | | | 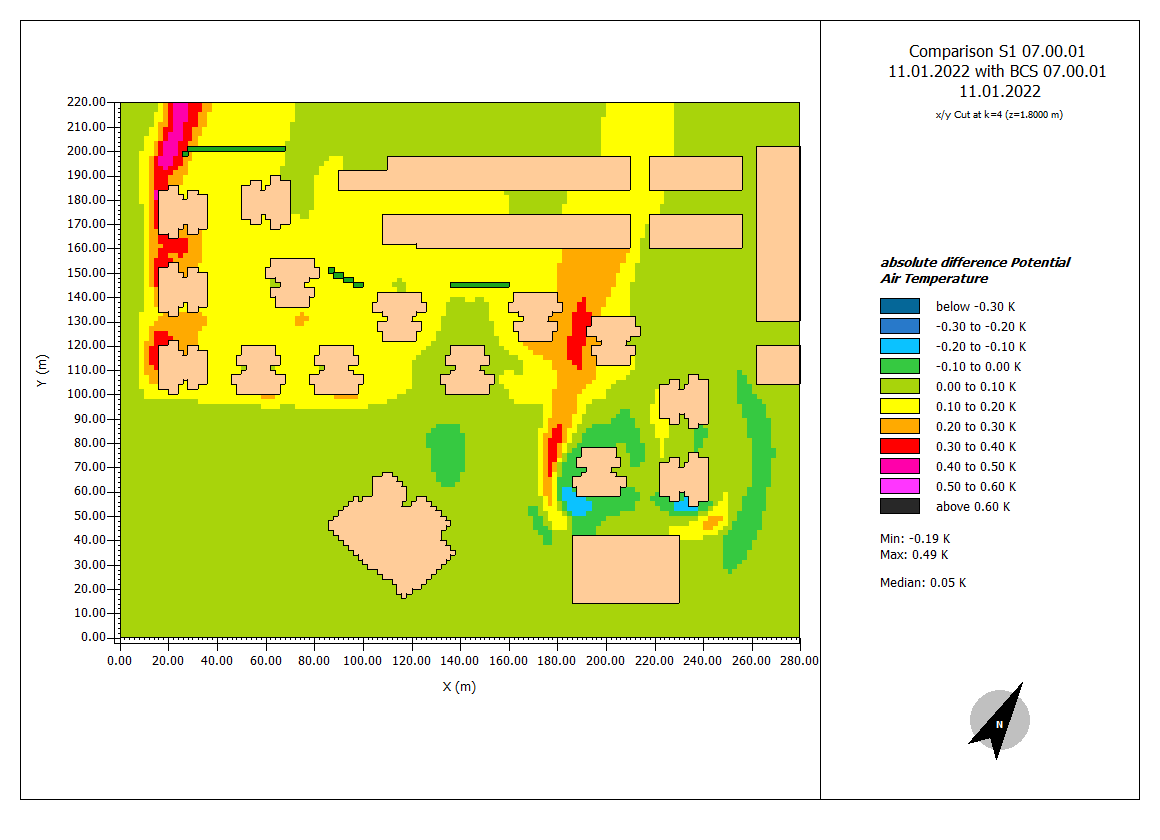 | |  |  |
| 1. S20 (spacing 2m) (Ficus LAD=5) | | | 1. S28 (spacing 2m) (Cono. LAD=5) | | | | | | | | Scale | |  |  |
| Figure B 14. AT differences between different scenarios and BCS at 7.00. | | | | | | | | | | | | |  |  |
| 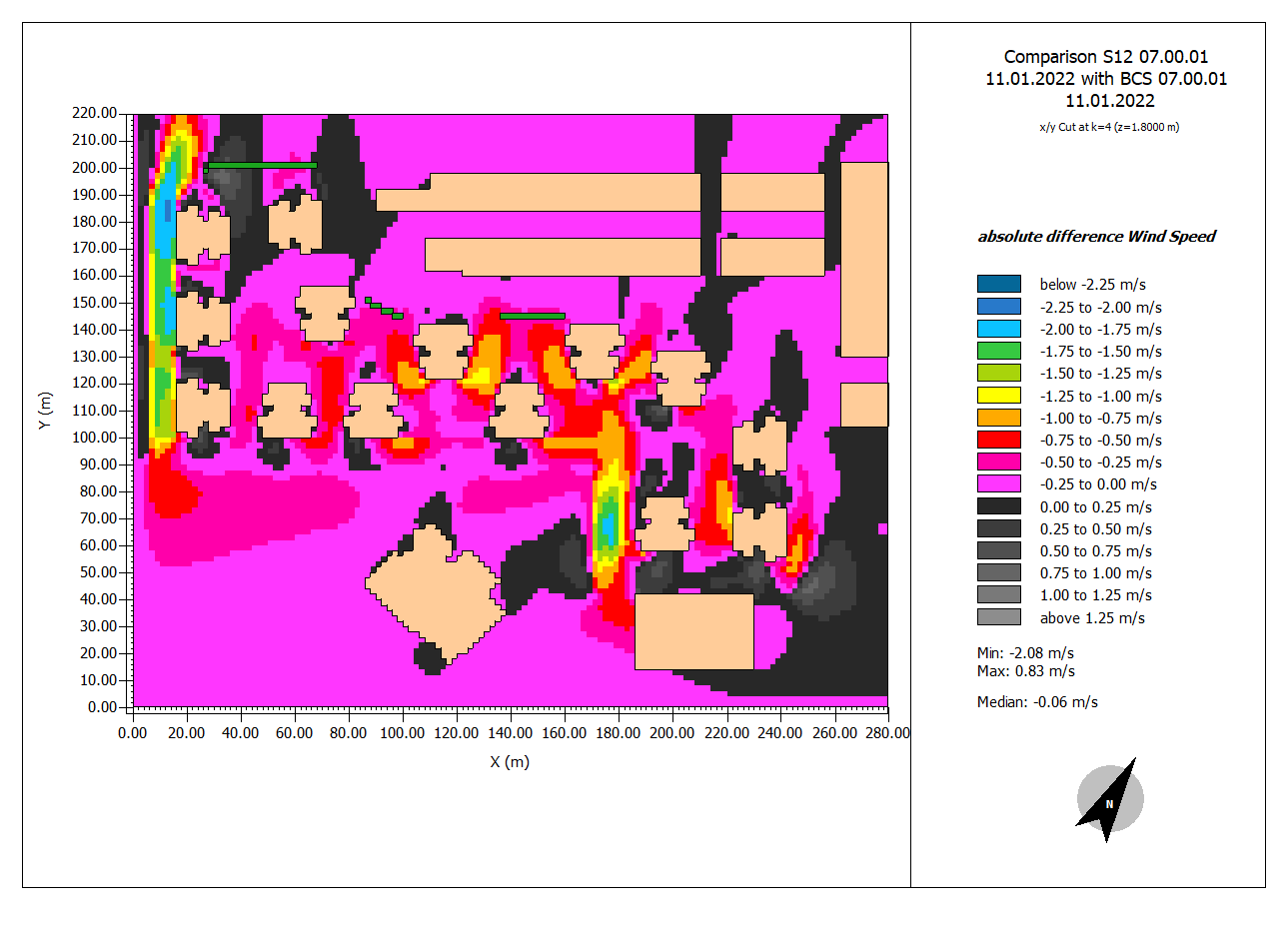 | | | 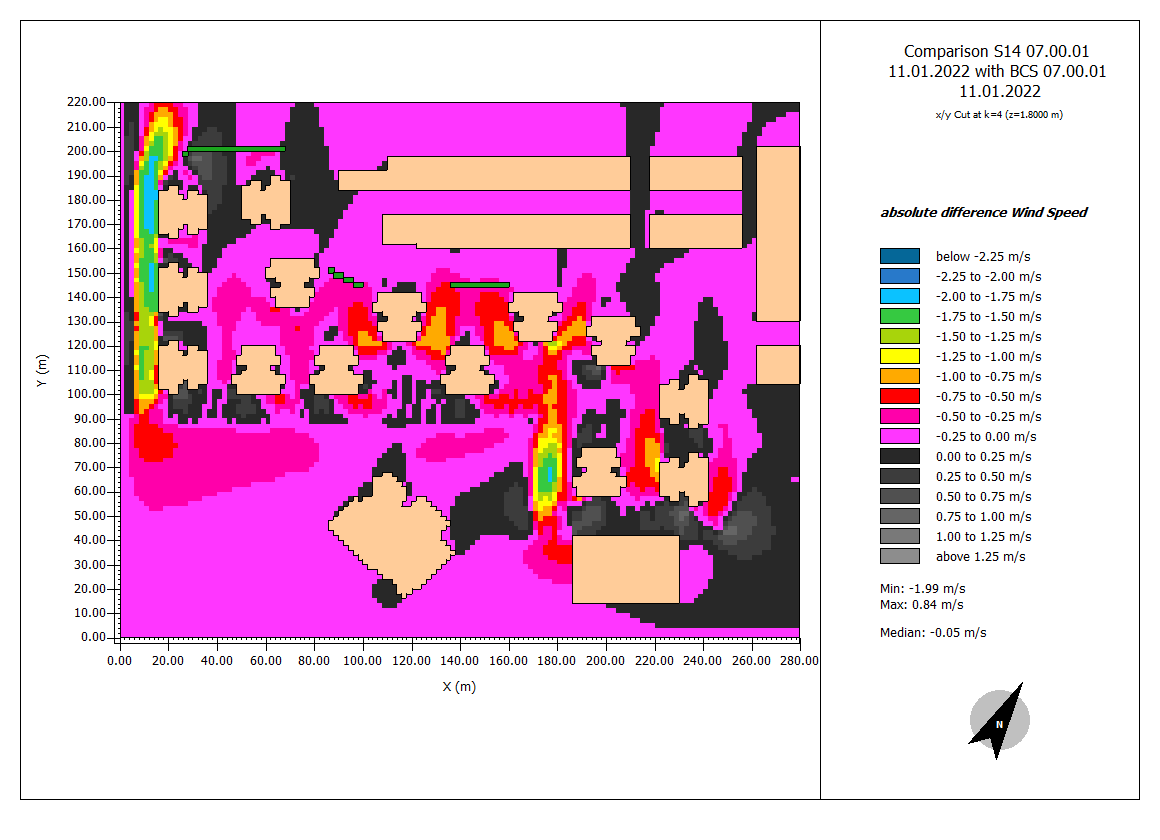 | | | | | | | | 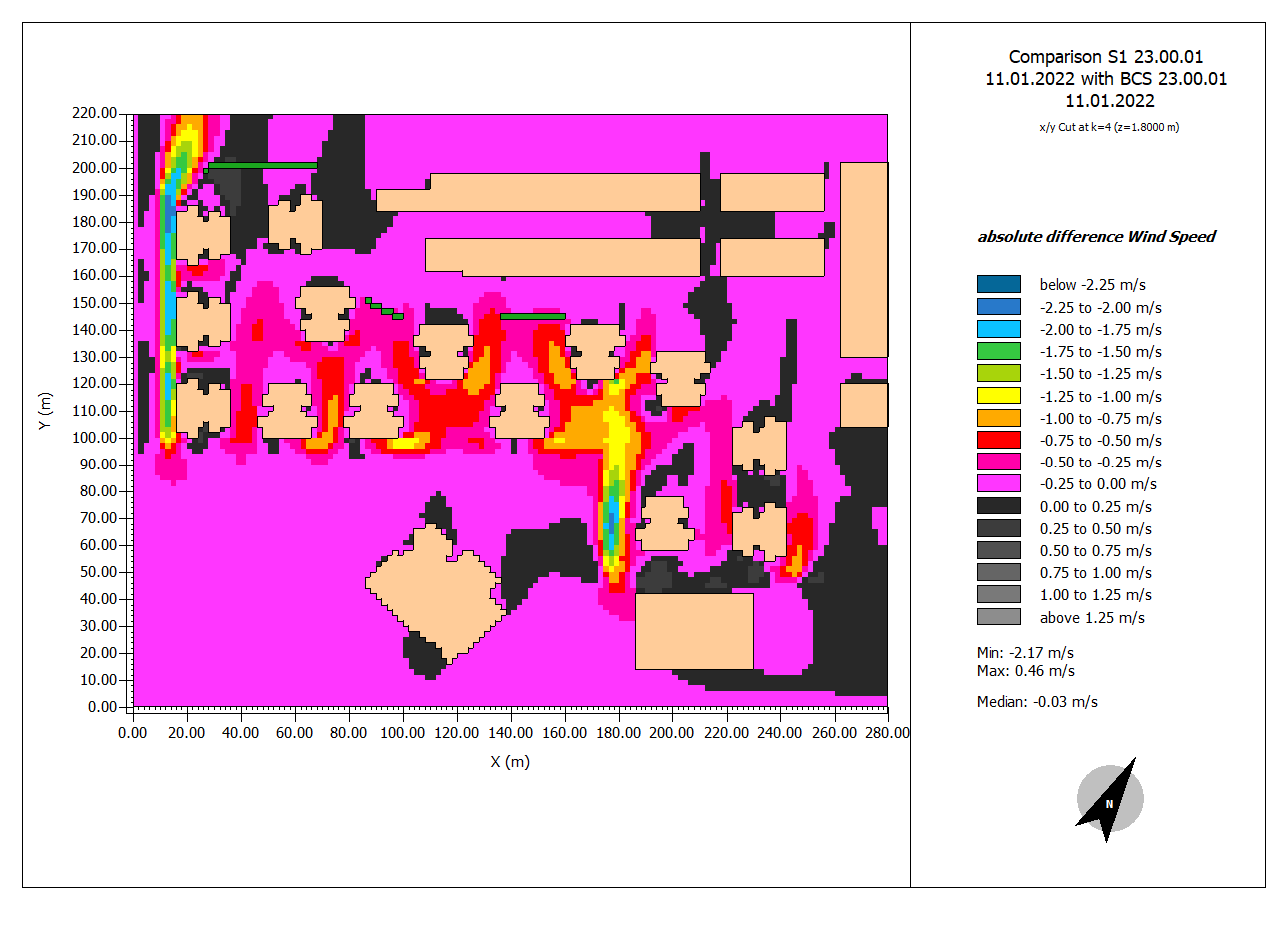 | |  |  |
| 1. S12 (spacing 2m) (Cono. LAD=2) | | | 1. S14 (spacing 6m) (Cono. LAD=2) | | | | | | | | Scale | |  |  |
| 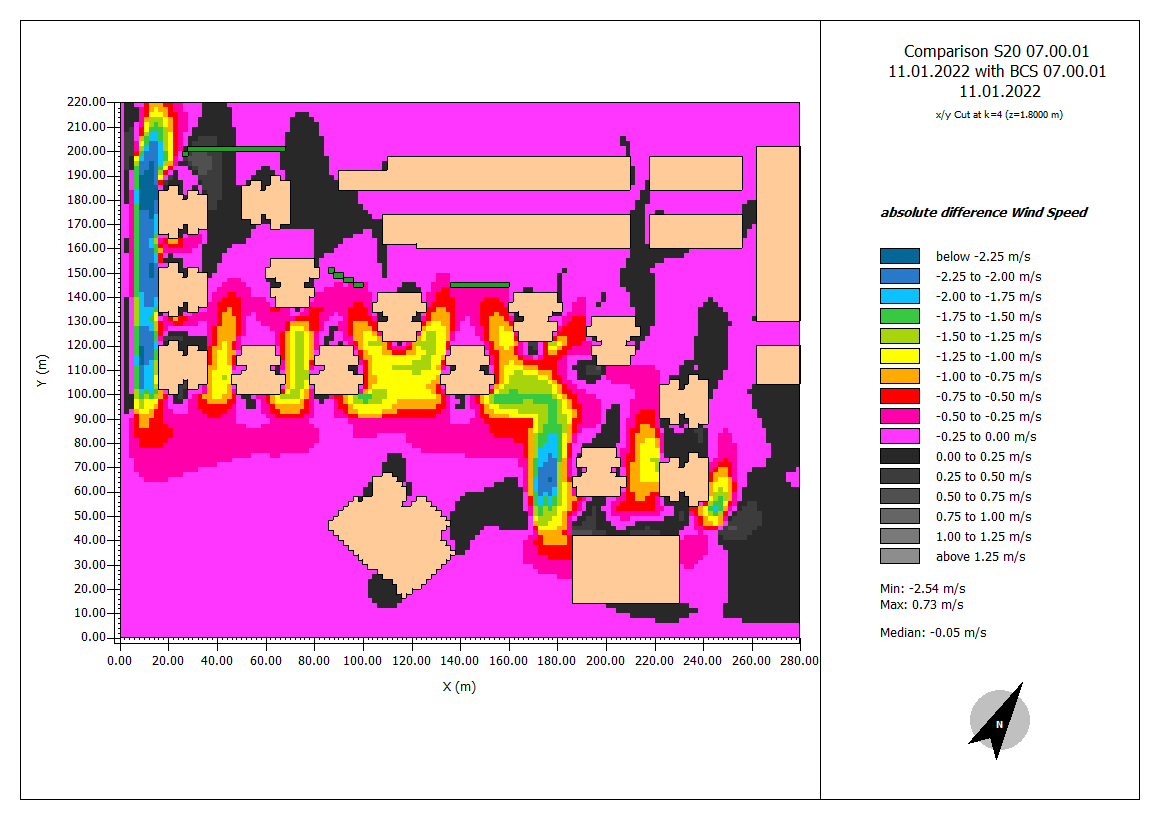 | | | 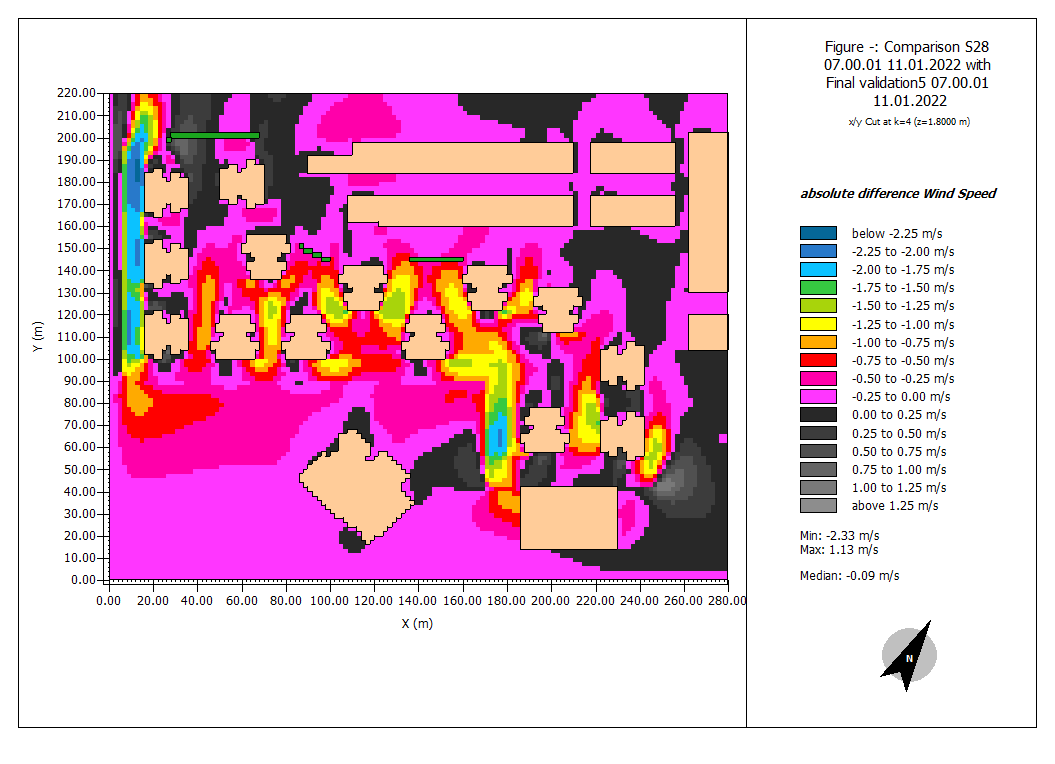 | | | | | | | | 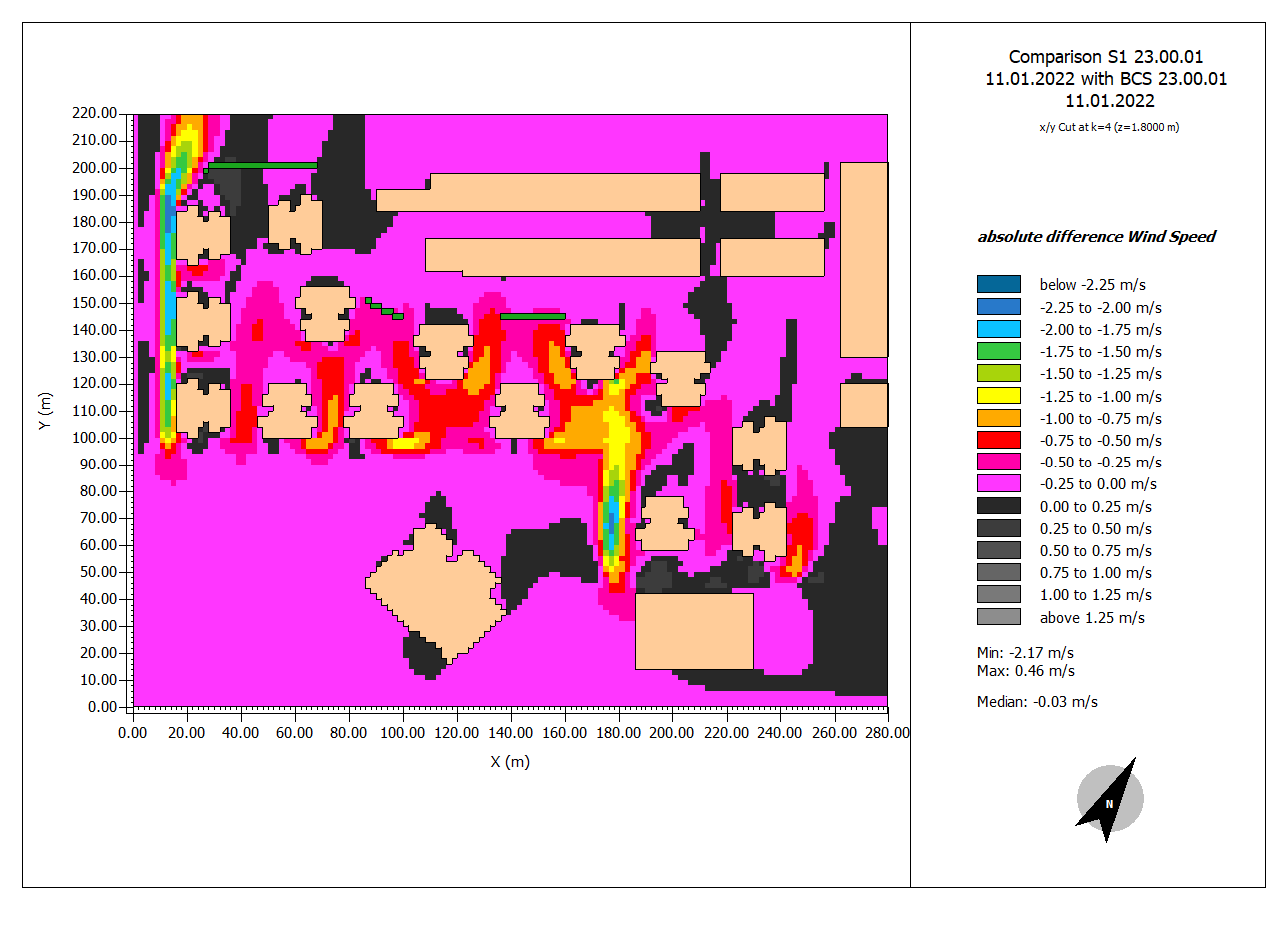 | |  |  |
| 1. S20 (spacing 2m) (Ficus LAD=5) | | | 1. S28 (spacing 2m) (Cono. LAD=5) | | | | | | | | Scale | |  |  |
| Figure B 15. Windspeed differences between different scenarios and BCS at 7.00. | | | | | | | | | | | | |  |  |

| 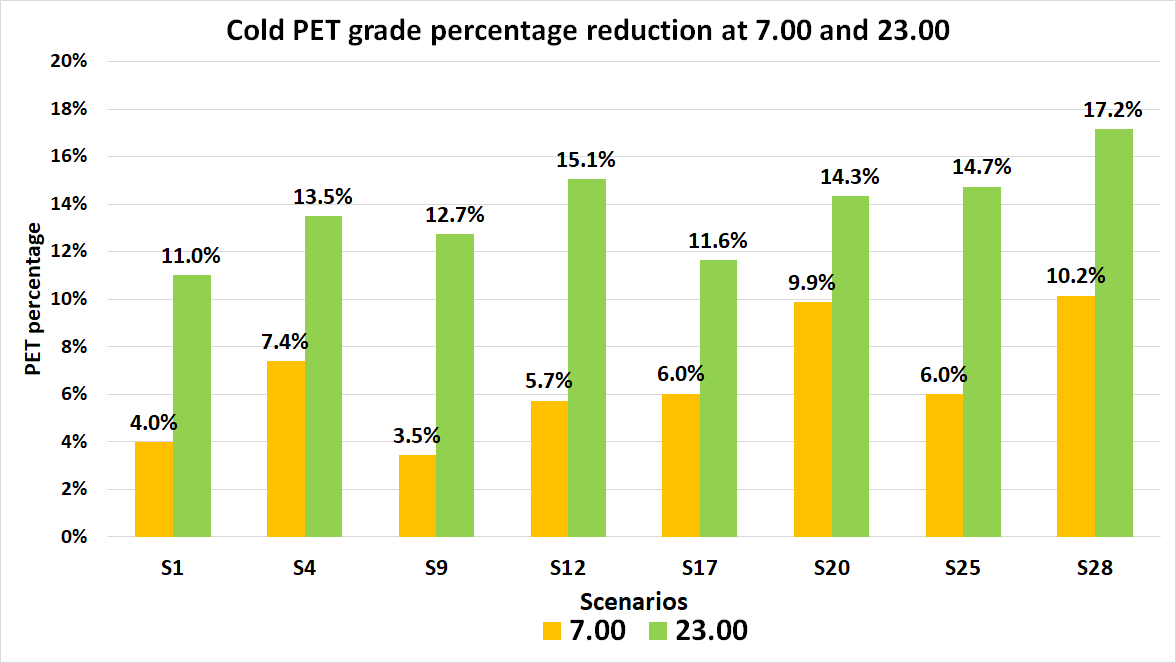 | | | | | | | |
| --- | --- | --- | --- | --- | --- | --- | --- |
| 1. Cold PET grade area reduction percentage for single and double rows scenarios compared to BCS in the early morning (7.00) and night (23.00). | | | | | | | |
| 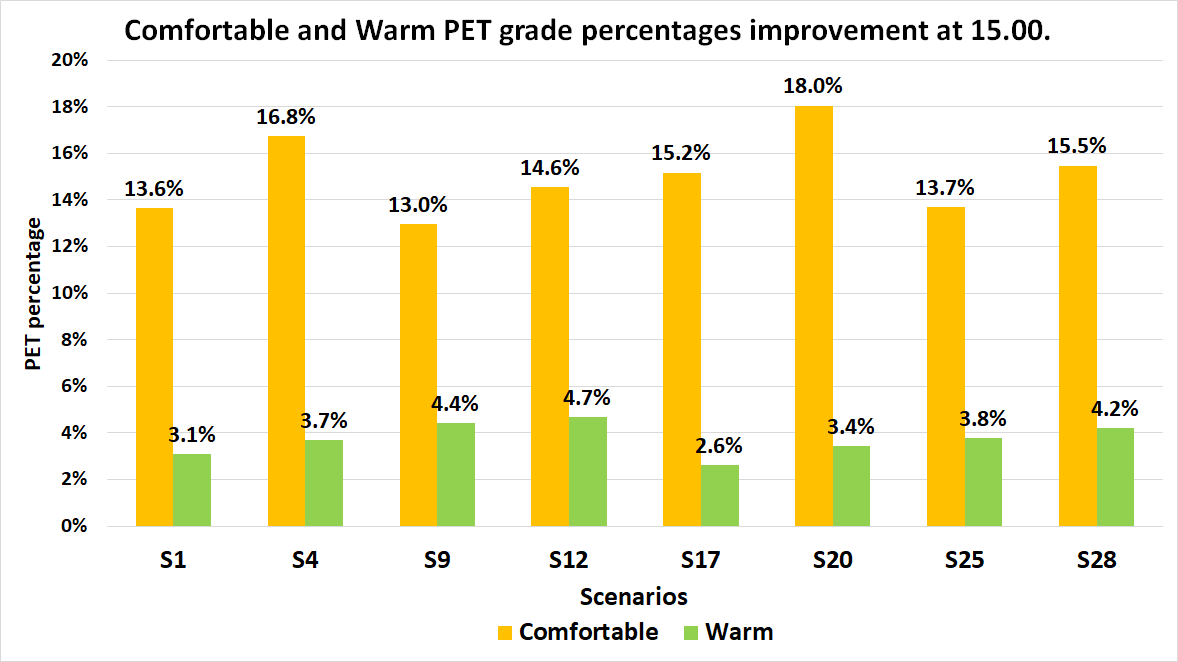 | | | | | | | |
| 1. Increasing comfortable grade area and reducing warm PET grade area percentage for single and double rows scenarios compared to BCS during daytime (15.00). | | | | | | | |
| Figure B 16. Cold, comfortable and warm PET grade area change percentage for single and double rows scenarios compared to BCS at different times. | | | | | | | |
| Double rows | | | | | | | |
| 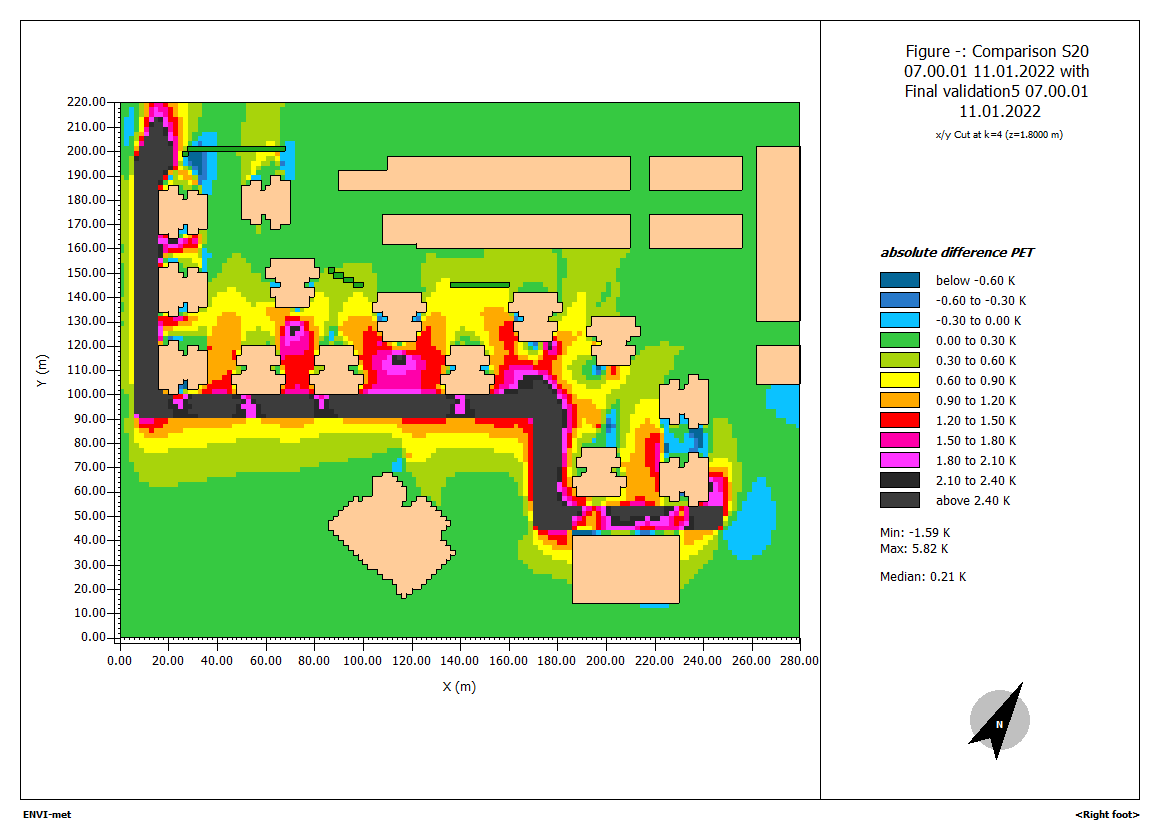 | | | 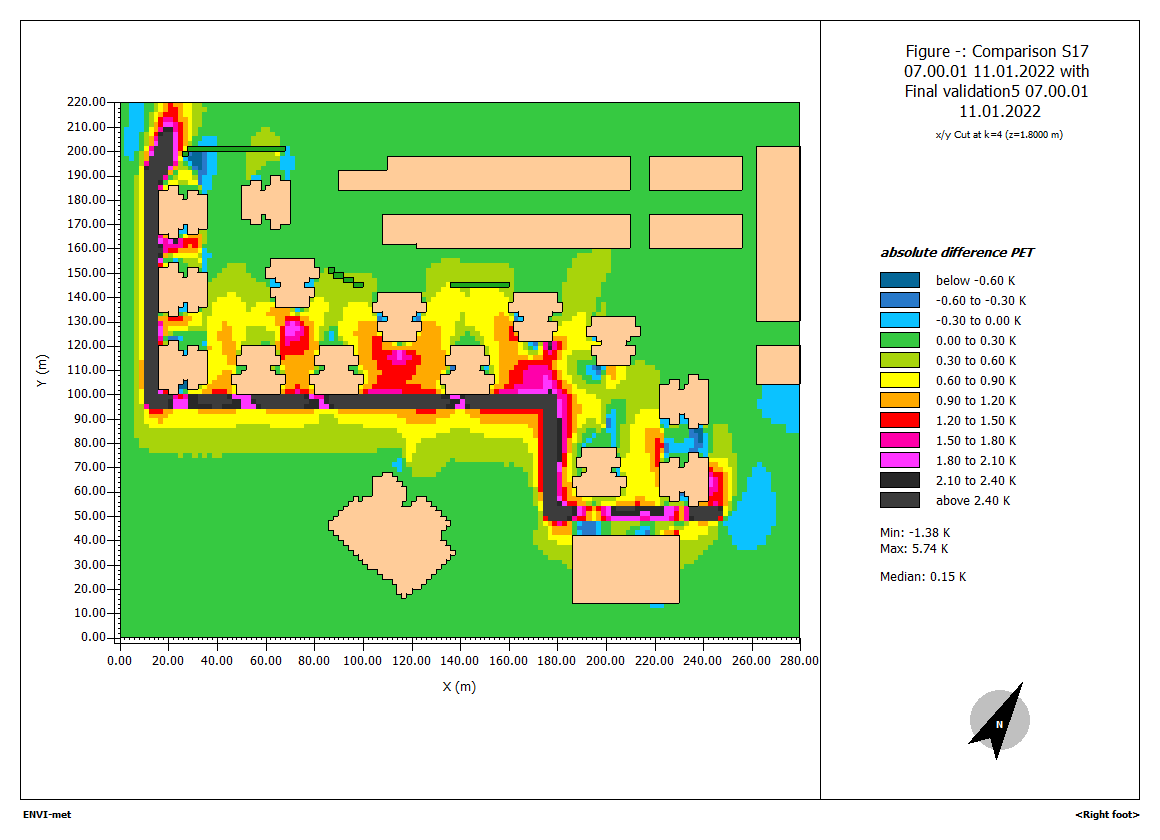 | | | | 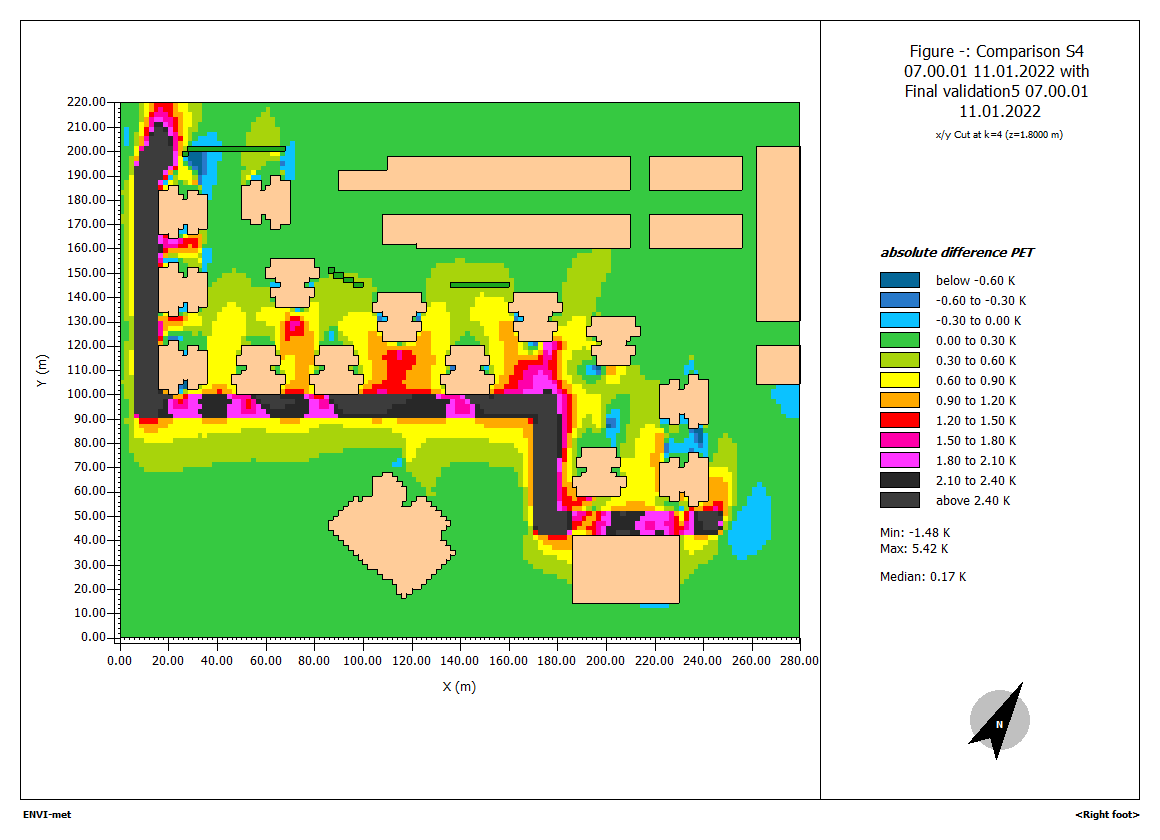 |
| 1. S20 (Double rows) (Ficus LAD=5) | | | 1. S17 (Single rows) (Ficus LAD=5) | | | | Scale |
| 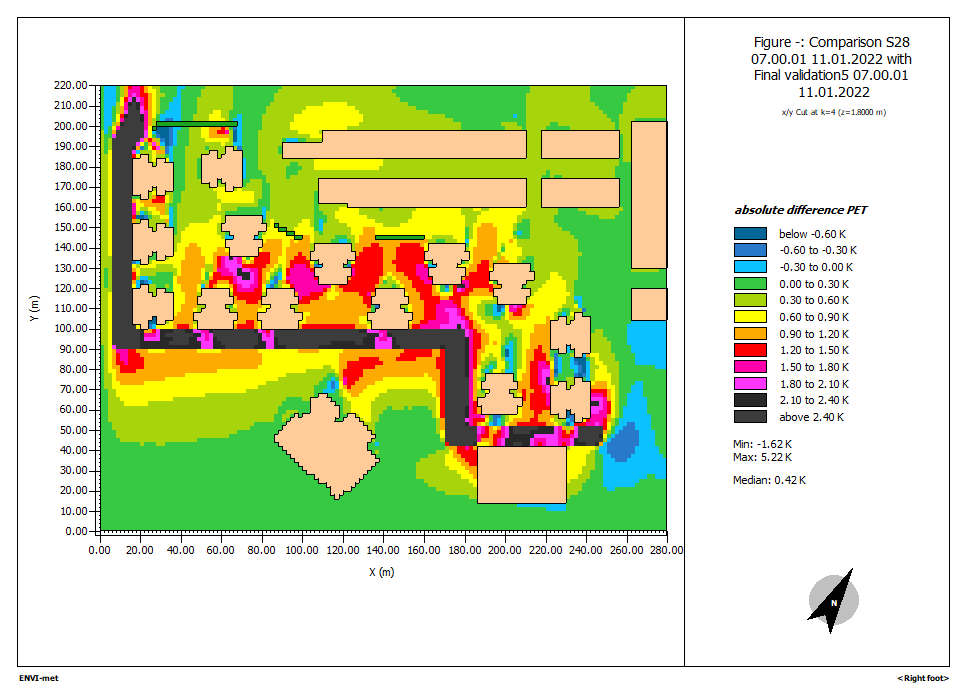 | | | 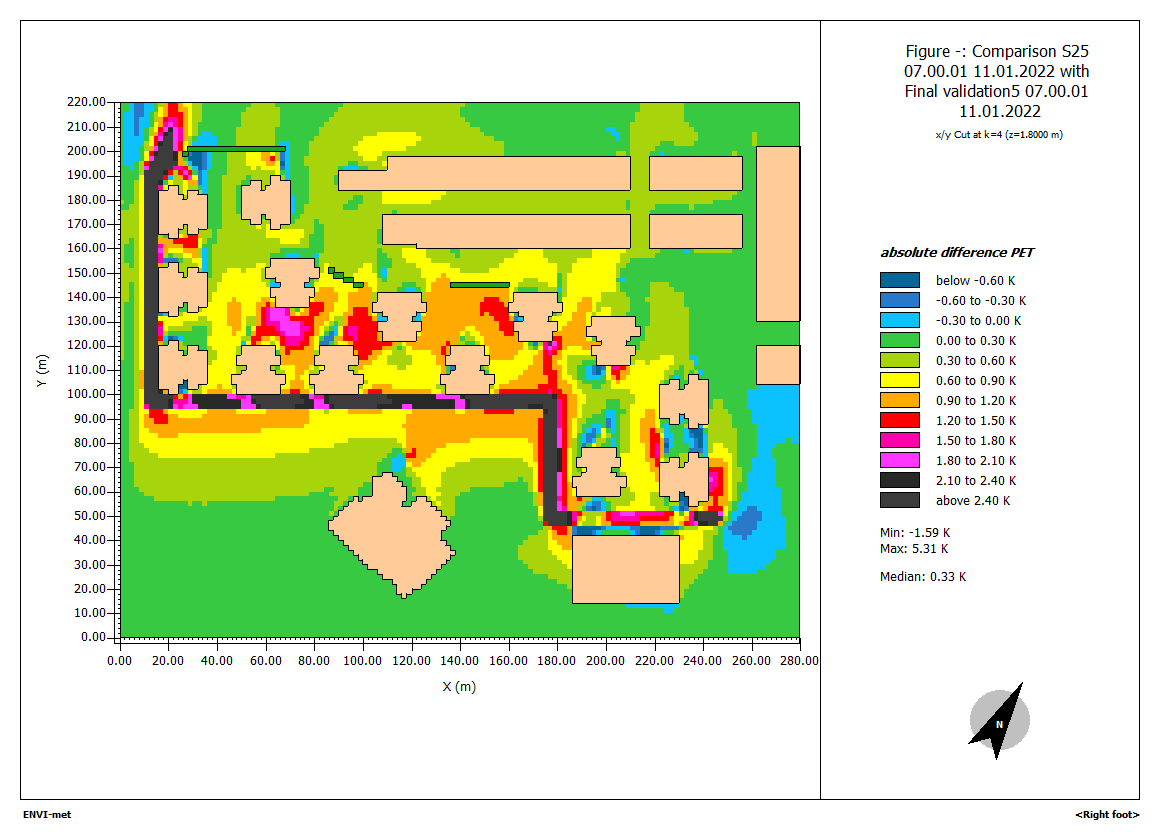 | | | | 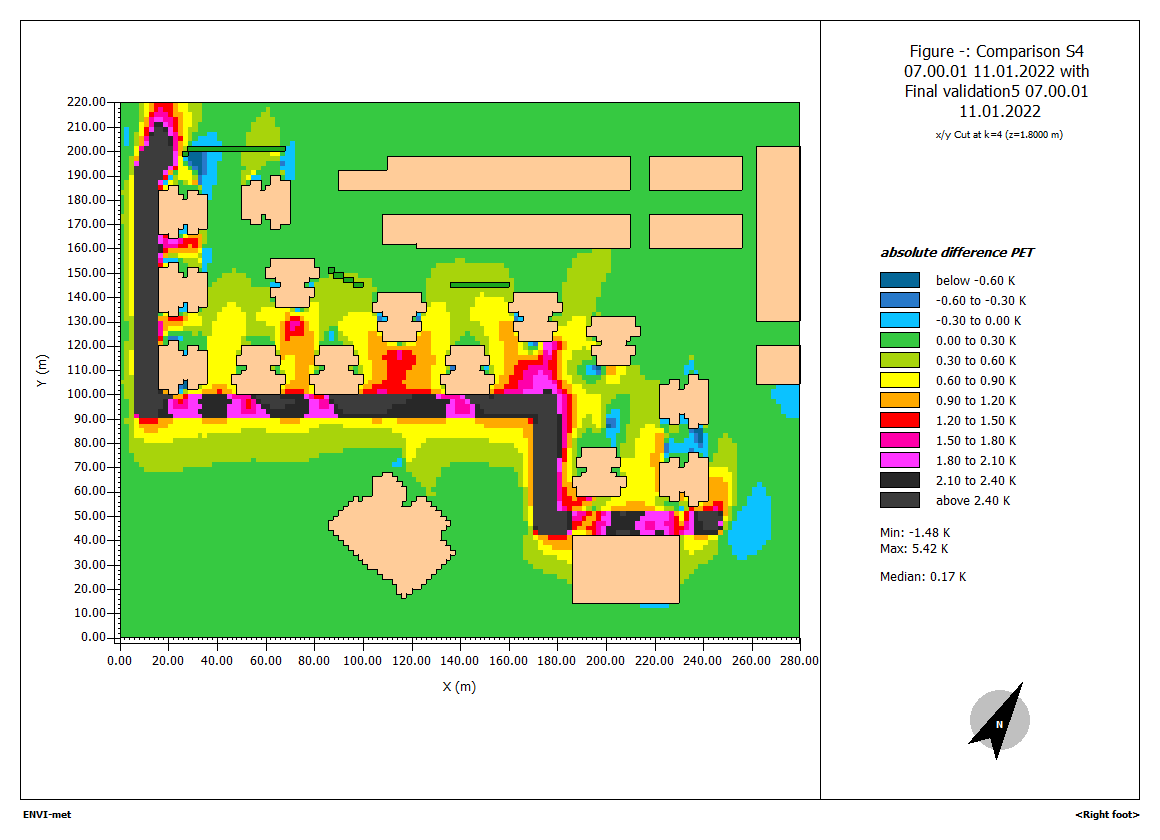 |
| 1. S28 (Double rows) (Cono. LAD=5) | | | 1. S25 (Single rows) (Cono. LAD=5) | | | | Scale |
| Figure B 17. PET differences between different scenarios and BCS at 7.00. | | | | | | | |
| 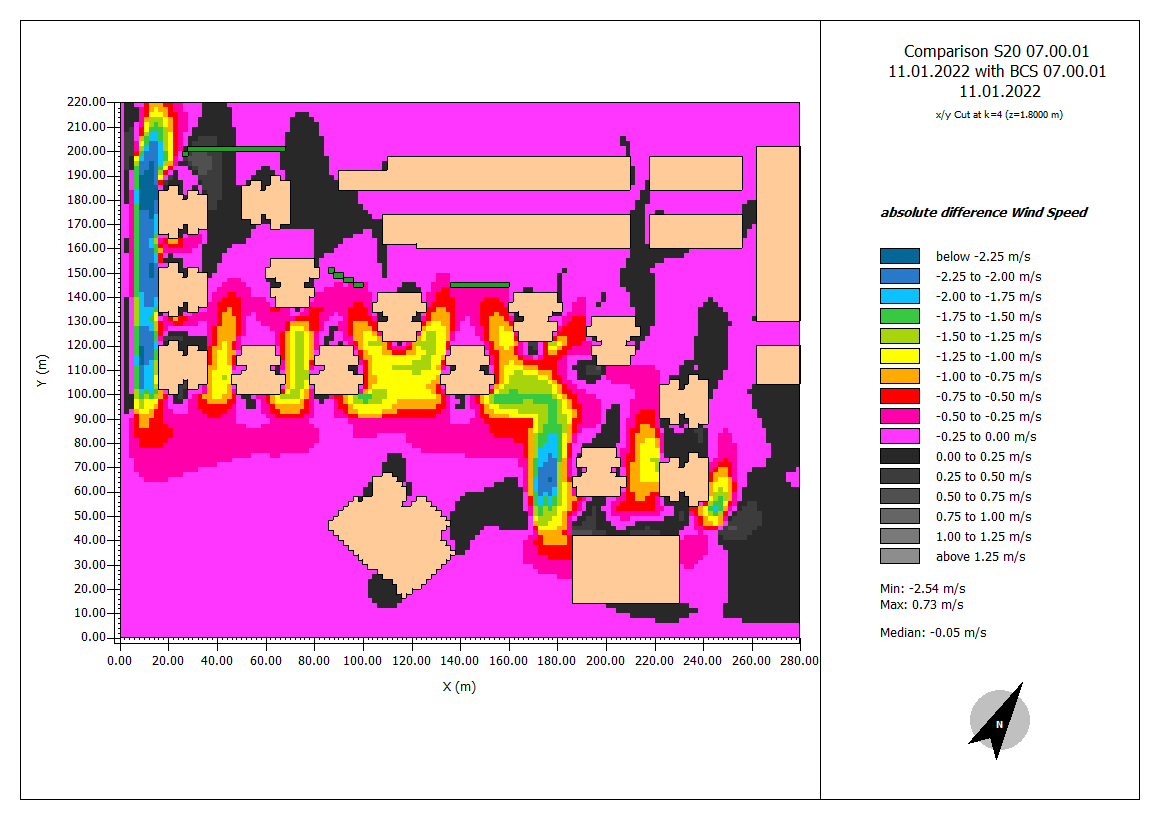 | | | 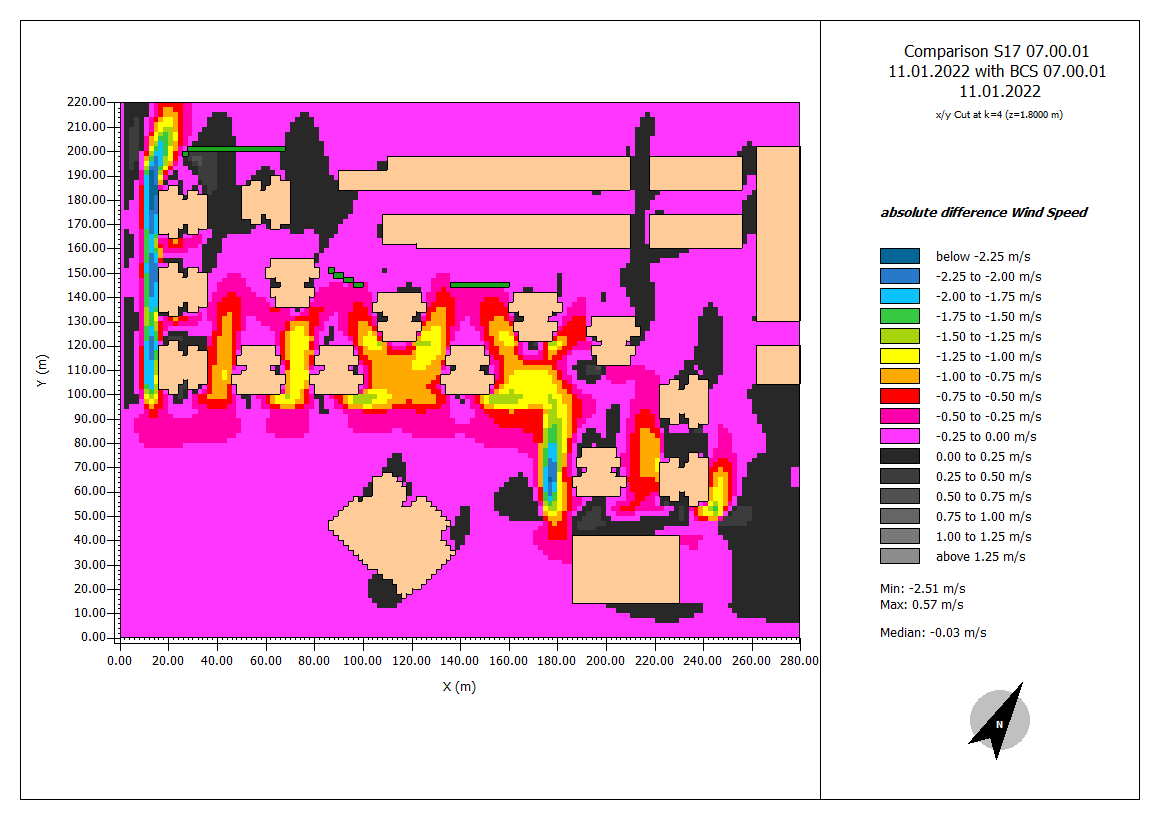 | | | | 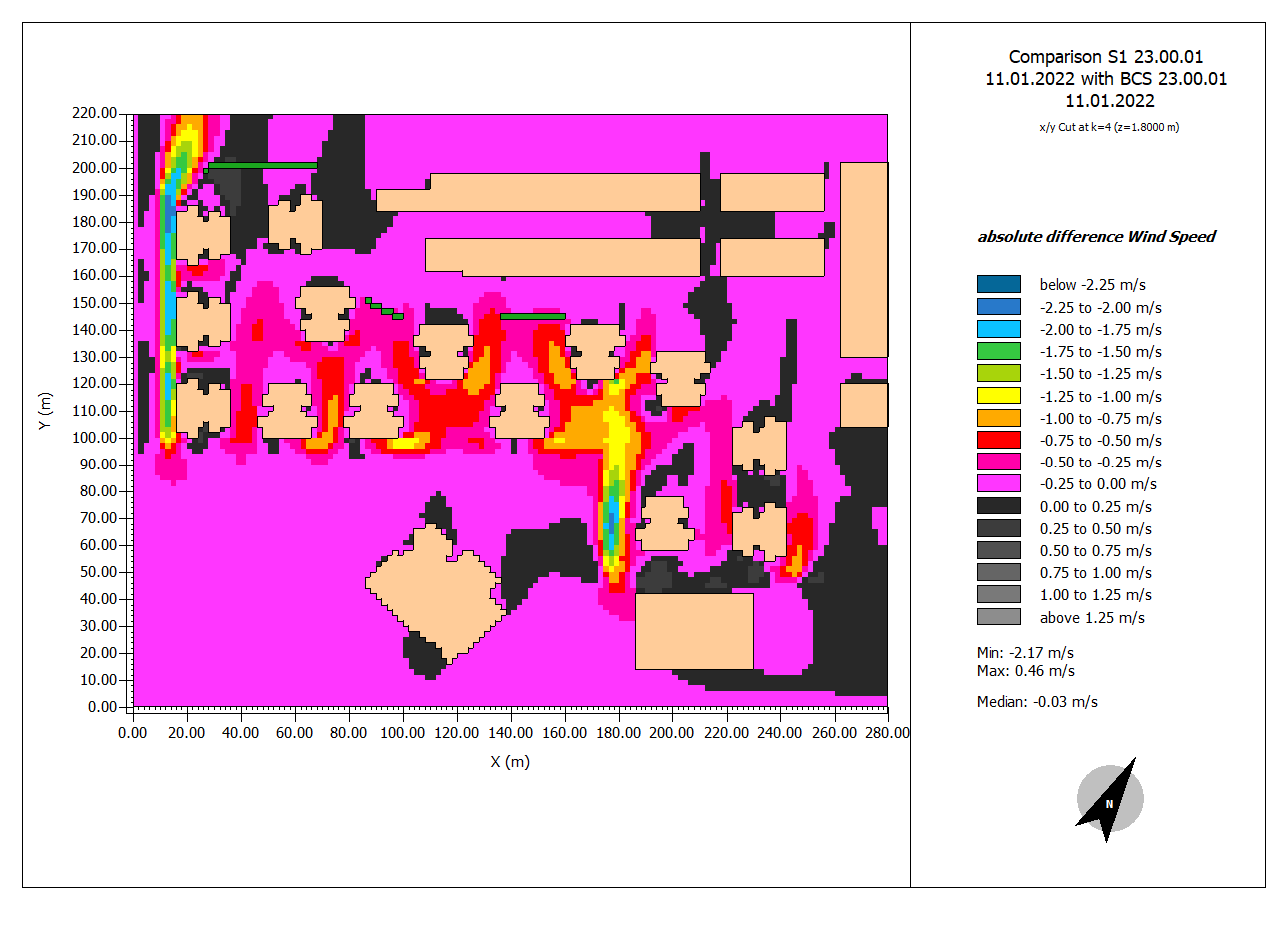 |
| 1. S20 (Double rows) (Ficus LAD=5) | | | 1. S17 (Single rows) (Ficus LAD=5) | | | | Scale |
| 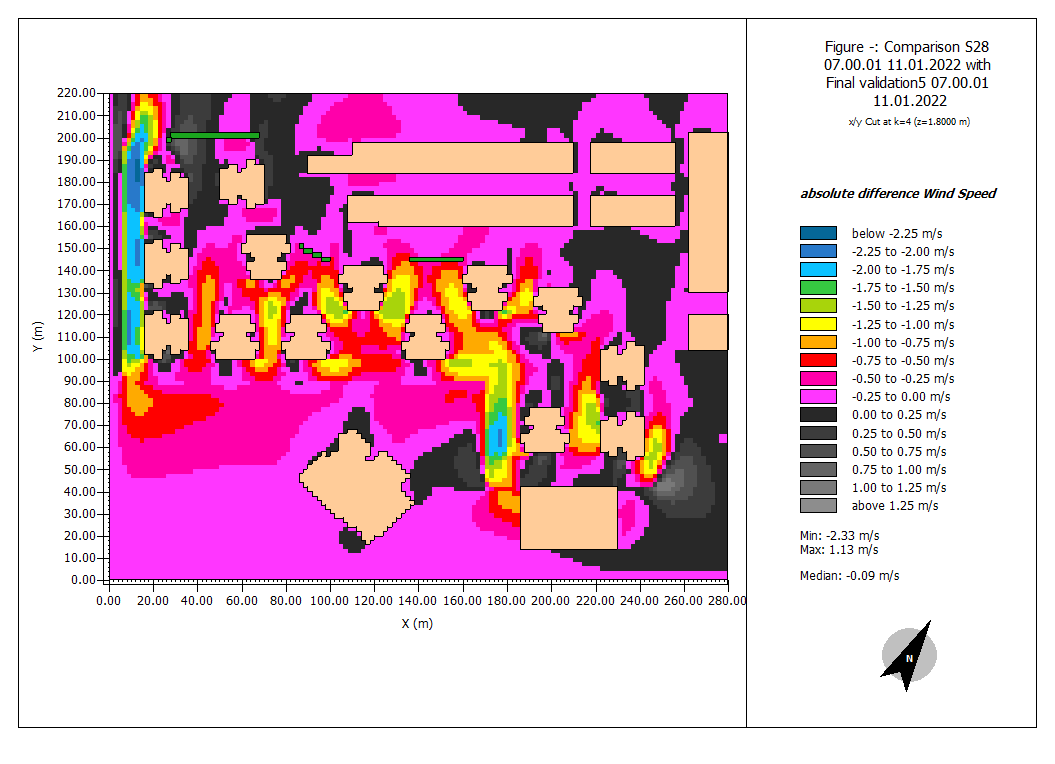 | | | 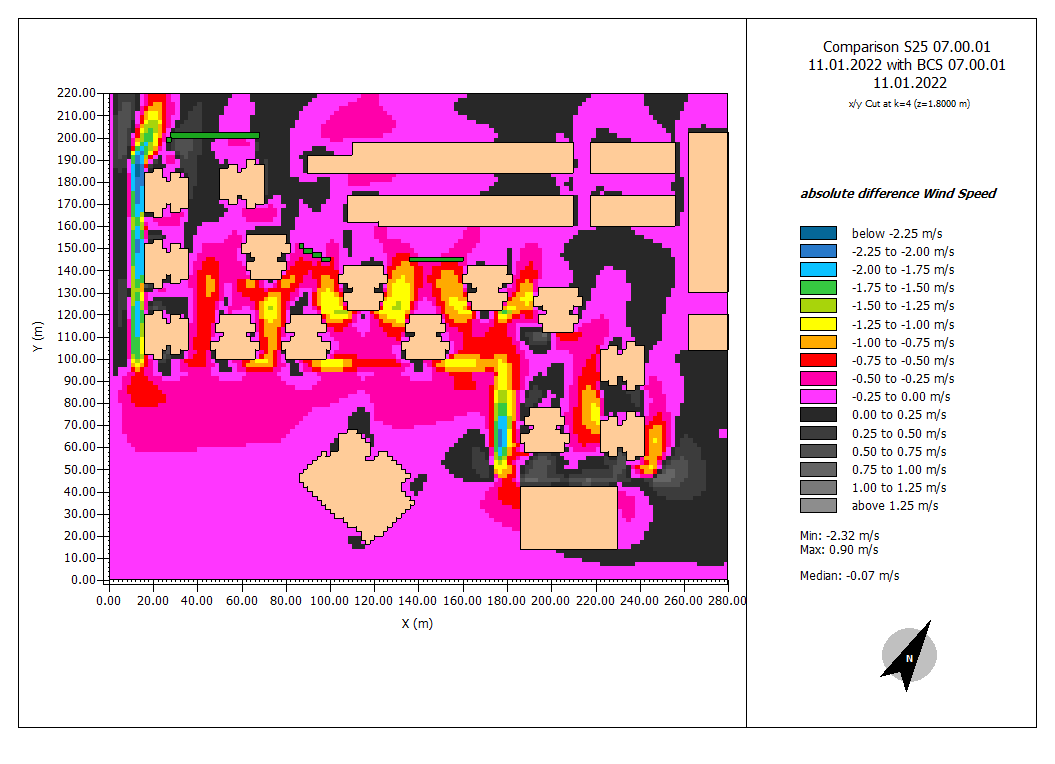 | | | | 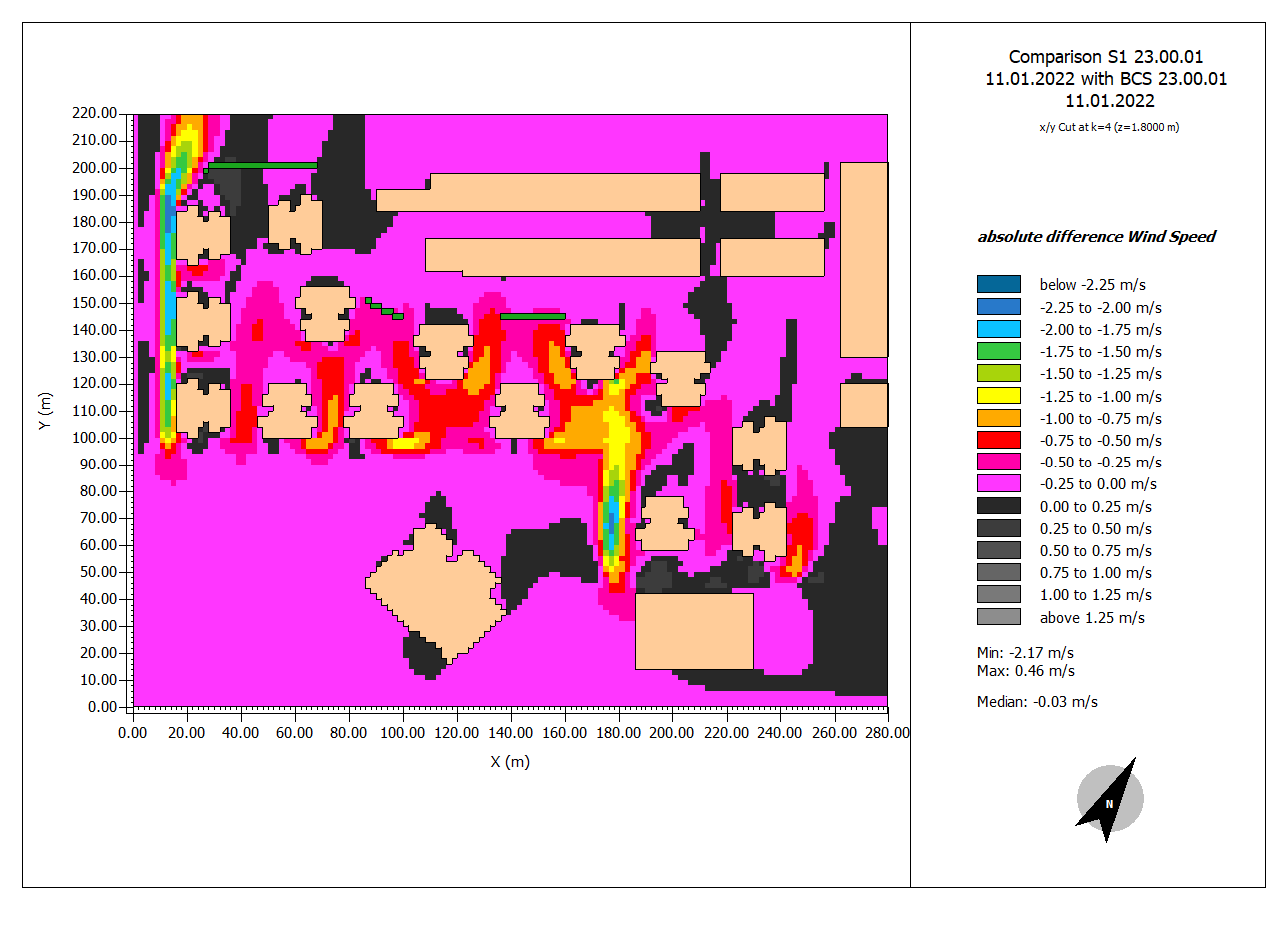 |
| 1. S28 (Double rows) (Cono. LAD=5) | | | 1. S25 (Single rows) (Cono. LAD=5) | | | | Scale |
| Figure B 18. Wind speed differences between different scenarios and BCS at 7.00. | | | | | | | |
| 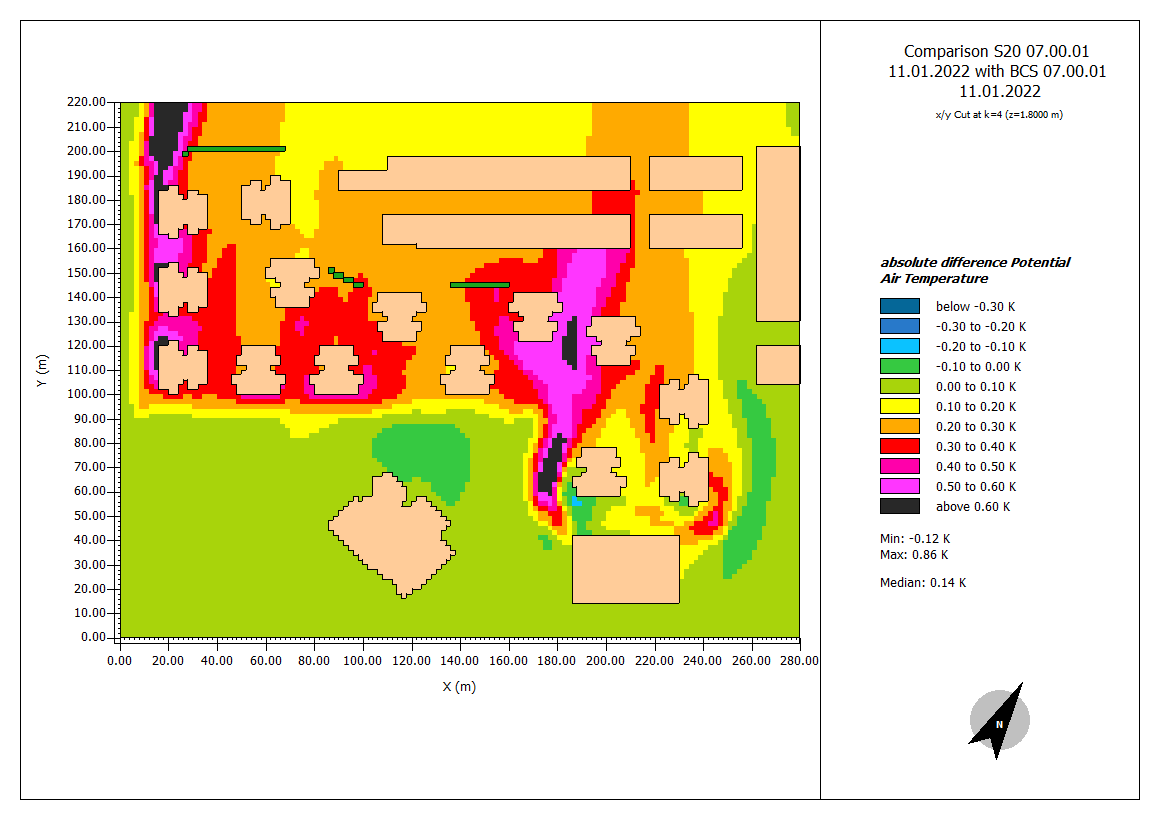 | | | 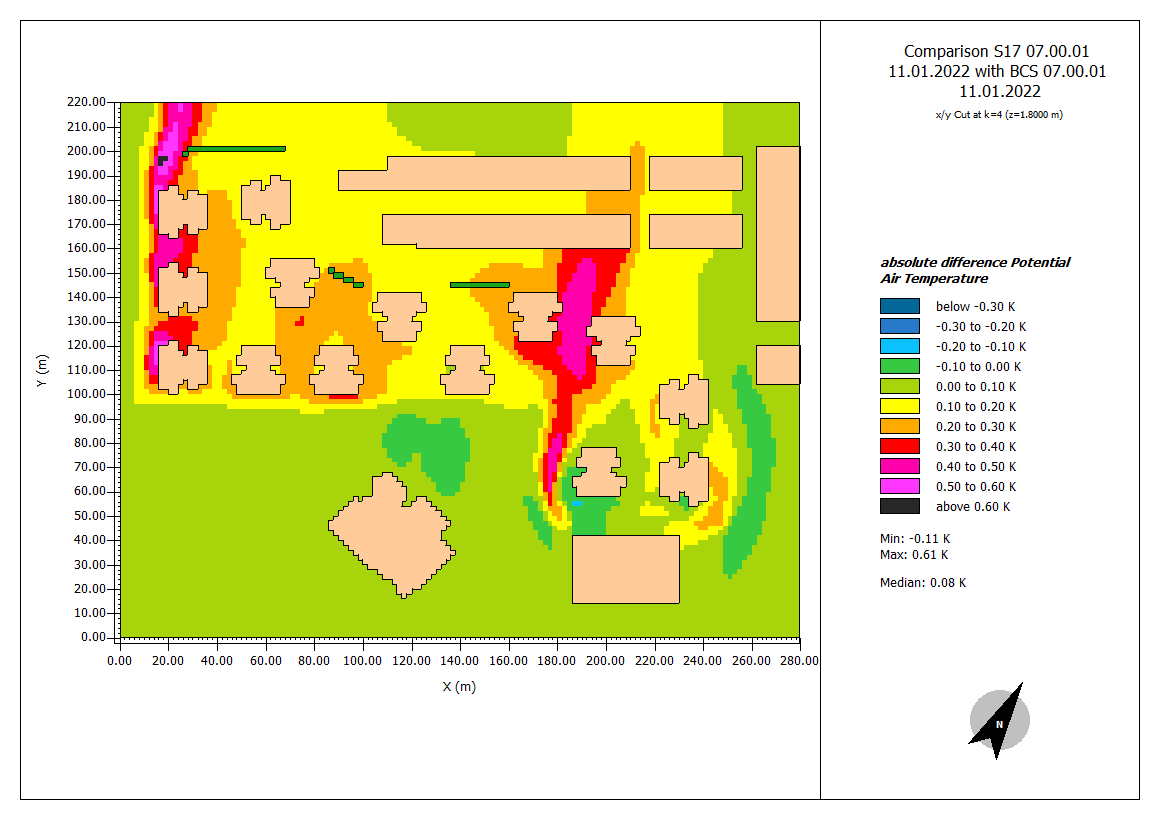 | | | | 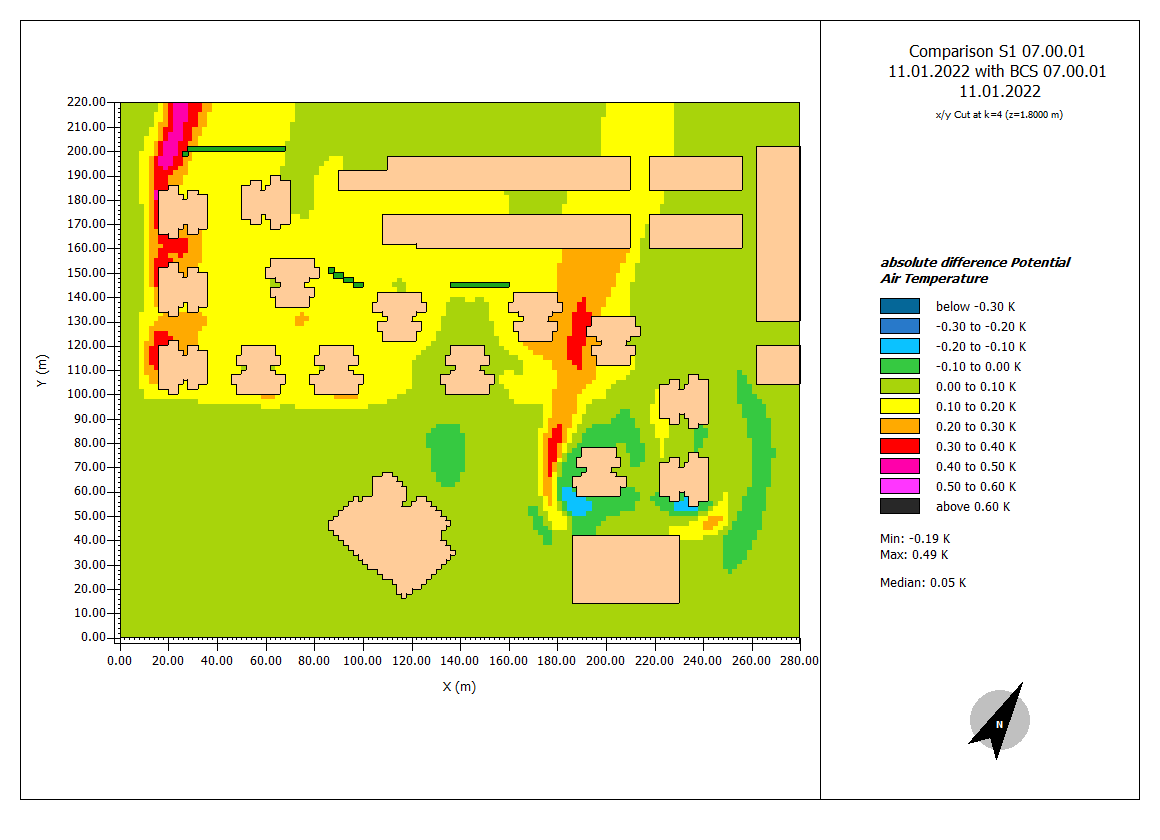 |
| 1. S20 (Double rows) (Ficus LAD=5) | | | 1. S17 (Single rows) (Ficus LAD=5) | | | | Scale |
| 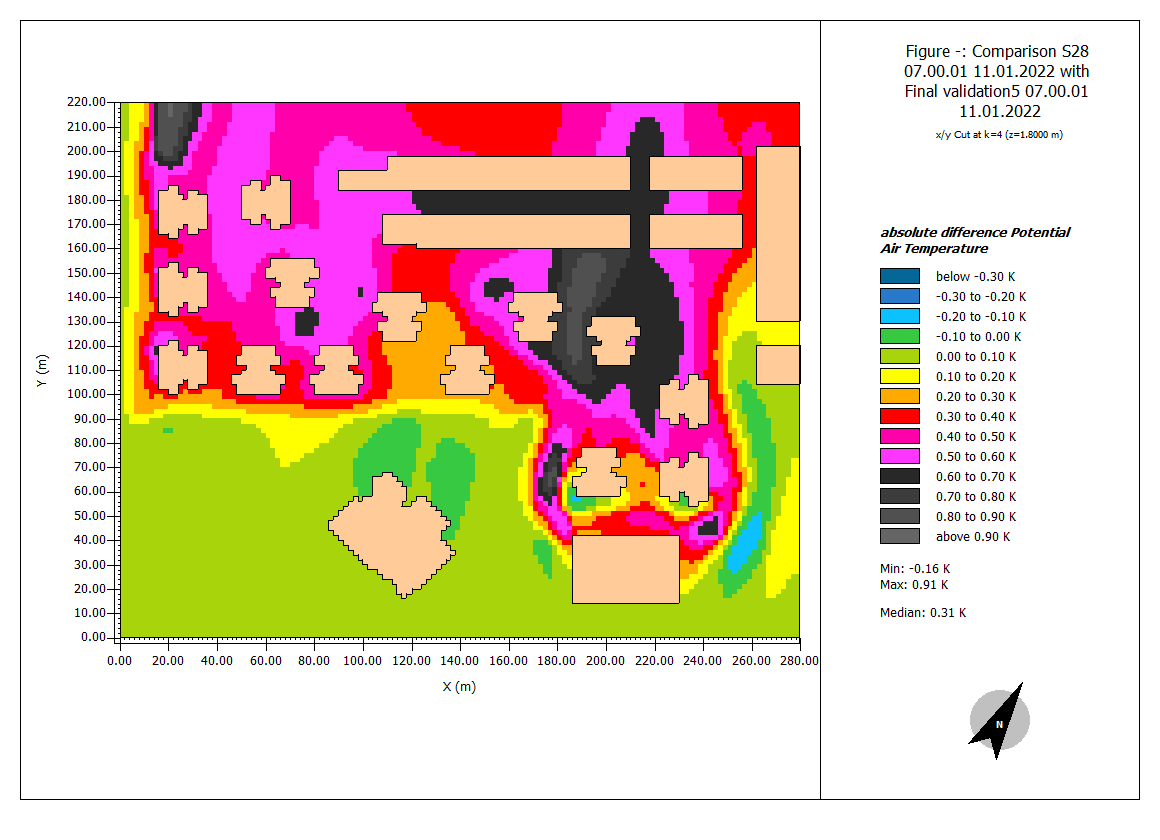 | | | 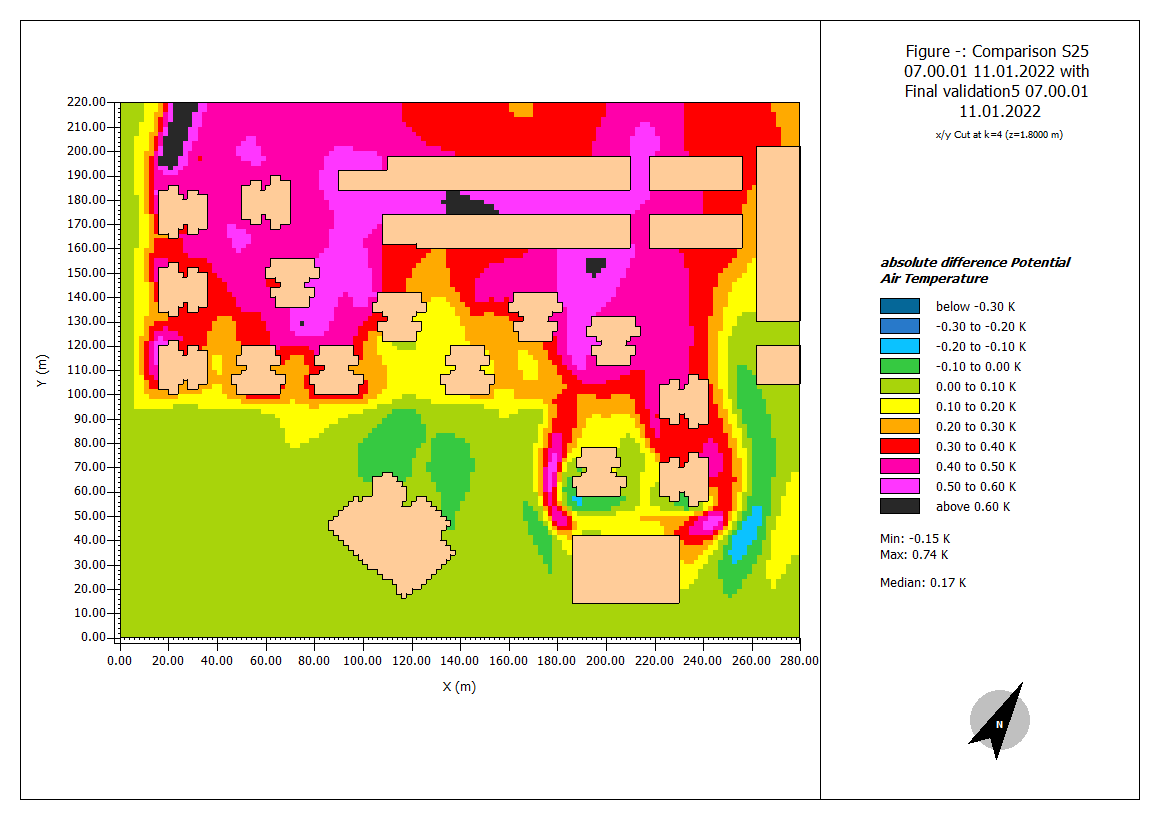 | | | | 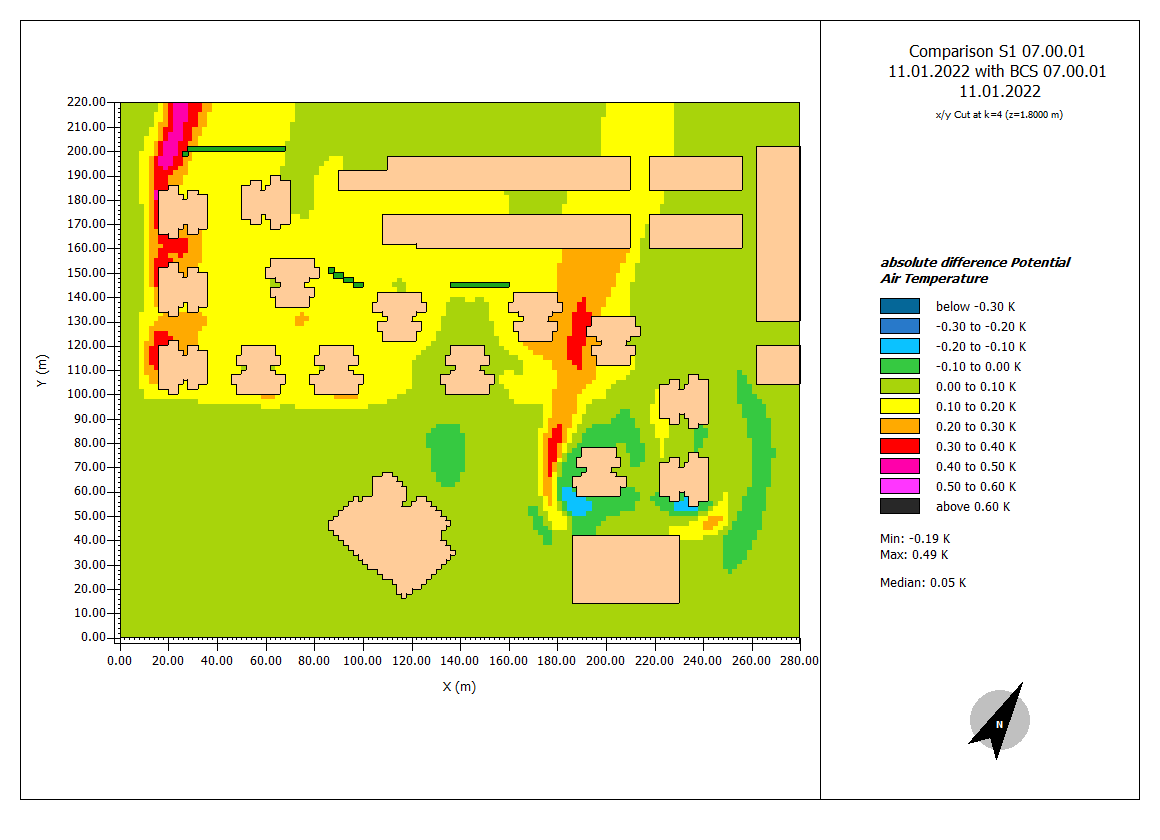 |
| 1. S28 (Double rows) (Cono. LAD=5) | | | 1. S25 (Single rows) (Cono. LAD=5) | | | | Scale |
| Figure B 19. (AT) at the pedestrian level differences between different scenarios and BCS at 7.00. | | | | | | | |
| 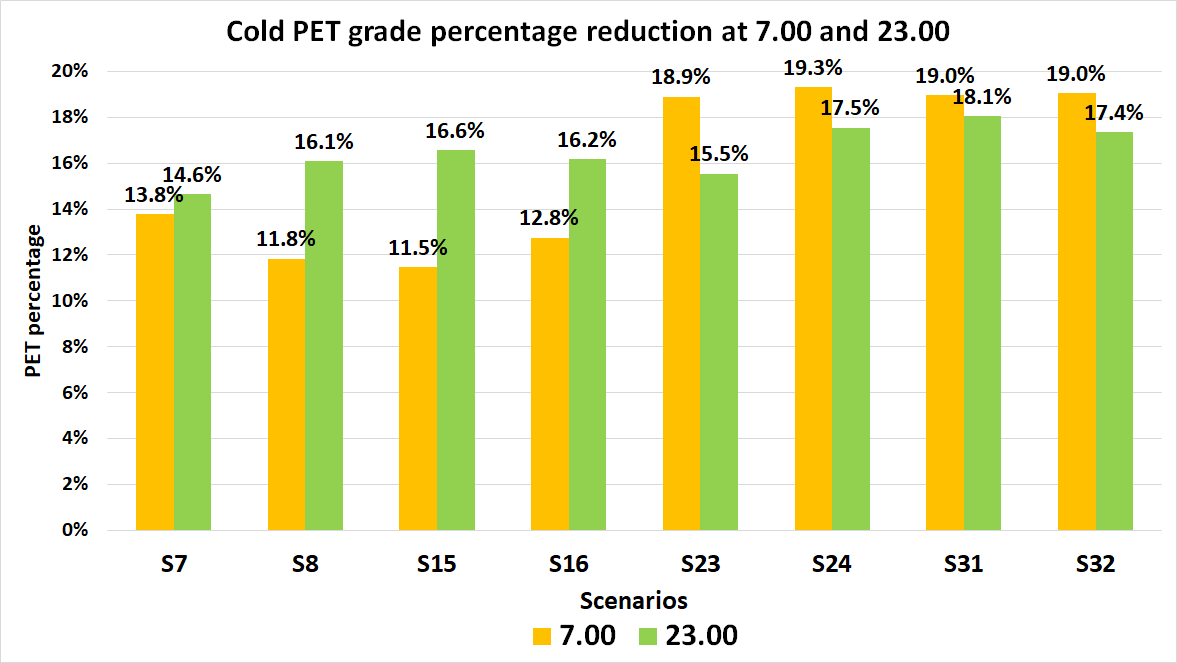 | | | | | | | |
| 1. Cold PET grade area reduction percentage for different tree distribution scenarios compared to BCS in the early morning (7.00) and night (23.00). | | | | | | | |
| 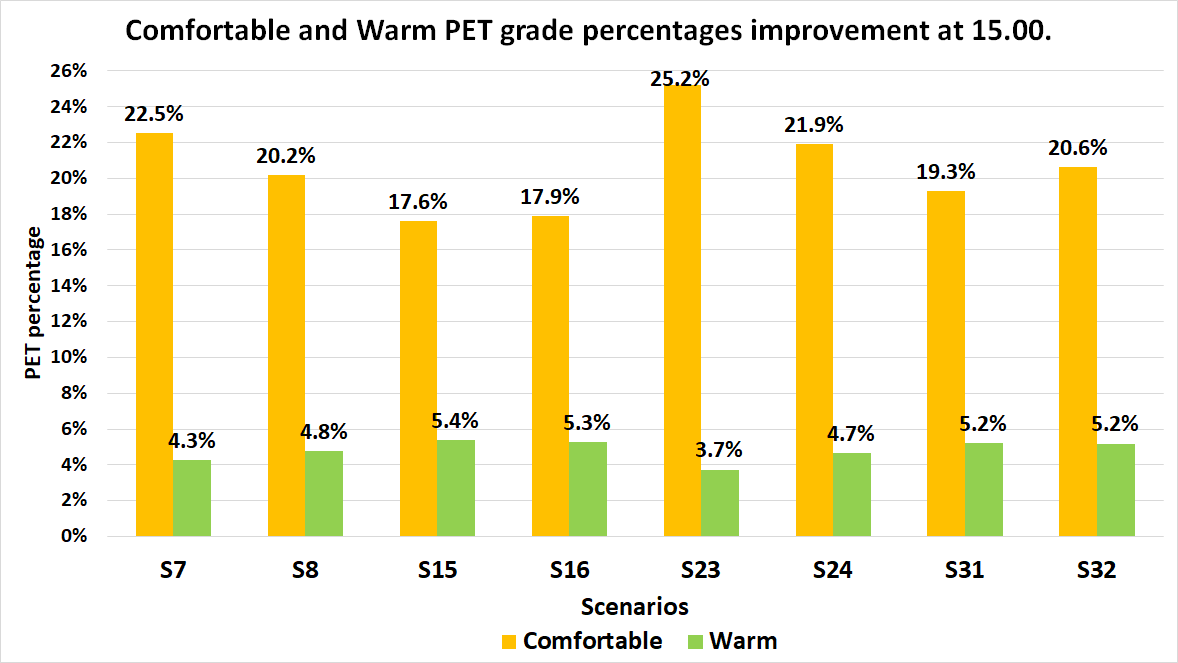 | | | | | | | |
| 1. Increasing comfortable grade area and reducing warm PET grade area percentage for different tree distribution scenarios compared to BCS during daytime (15.00). | | | | | | | |
| Figure B 20. Cold, comfortable and warm PET grade area change percentage for different tree distribution scenarios compared to BCS at different times. | | | | | | | |
| 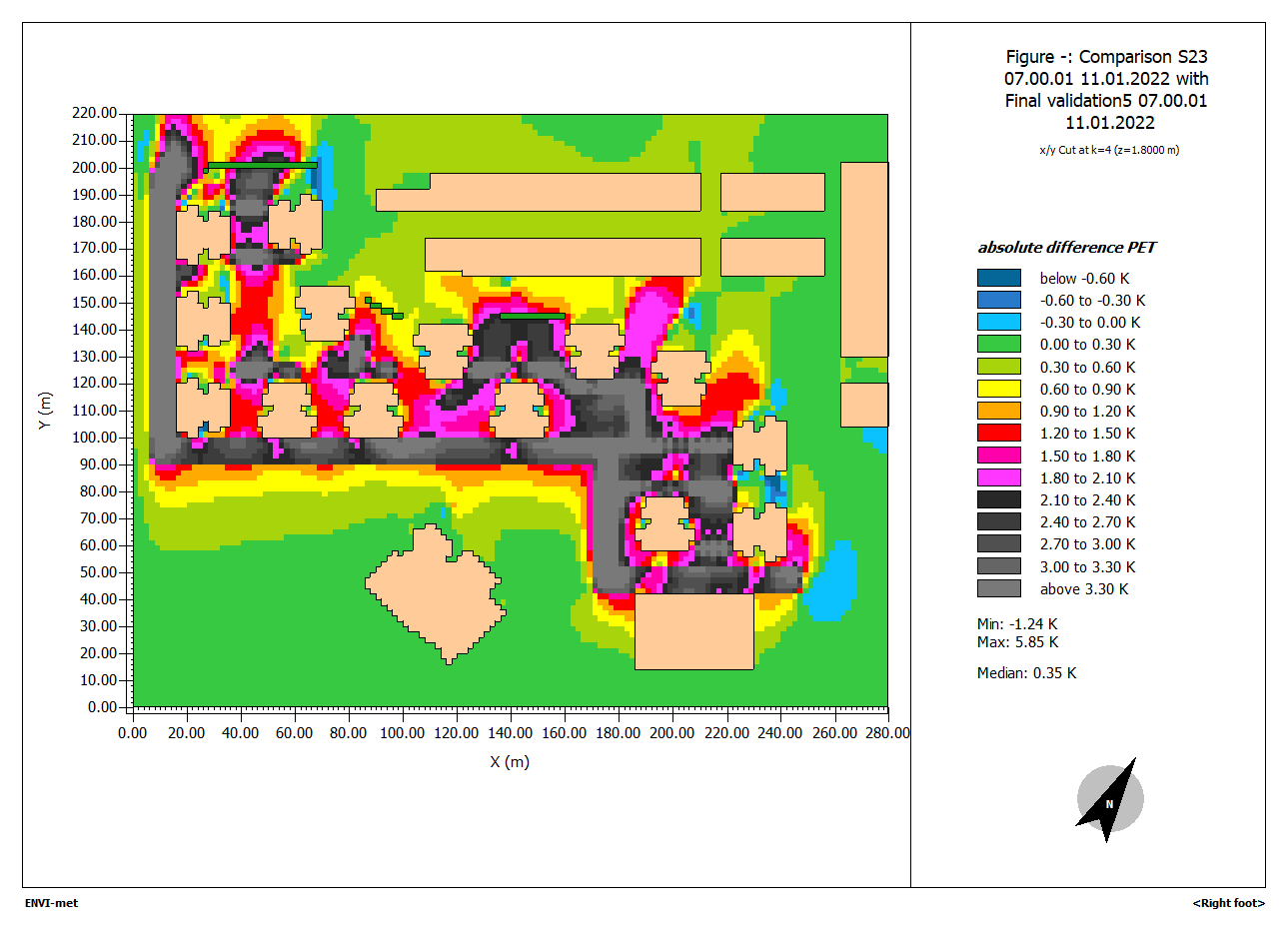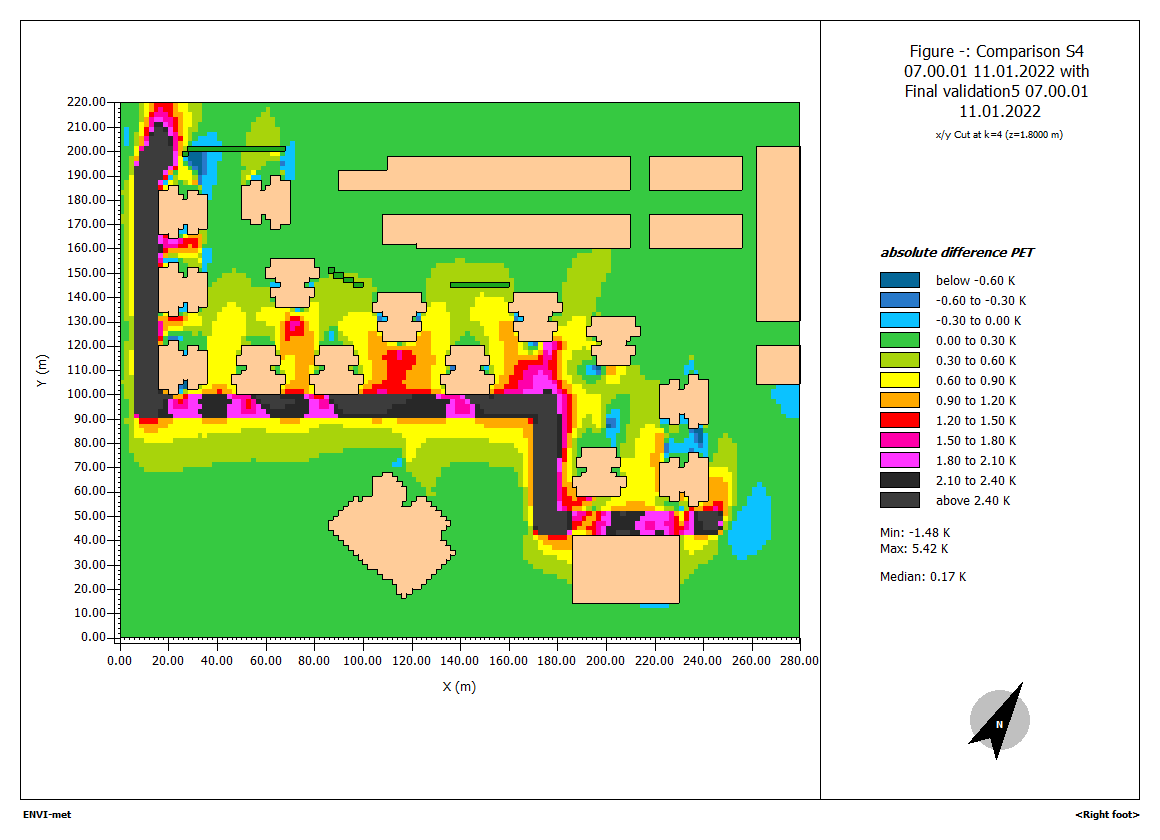 | | | | 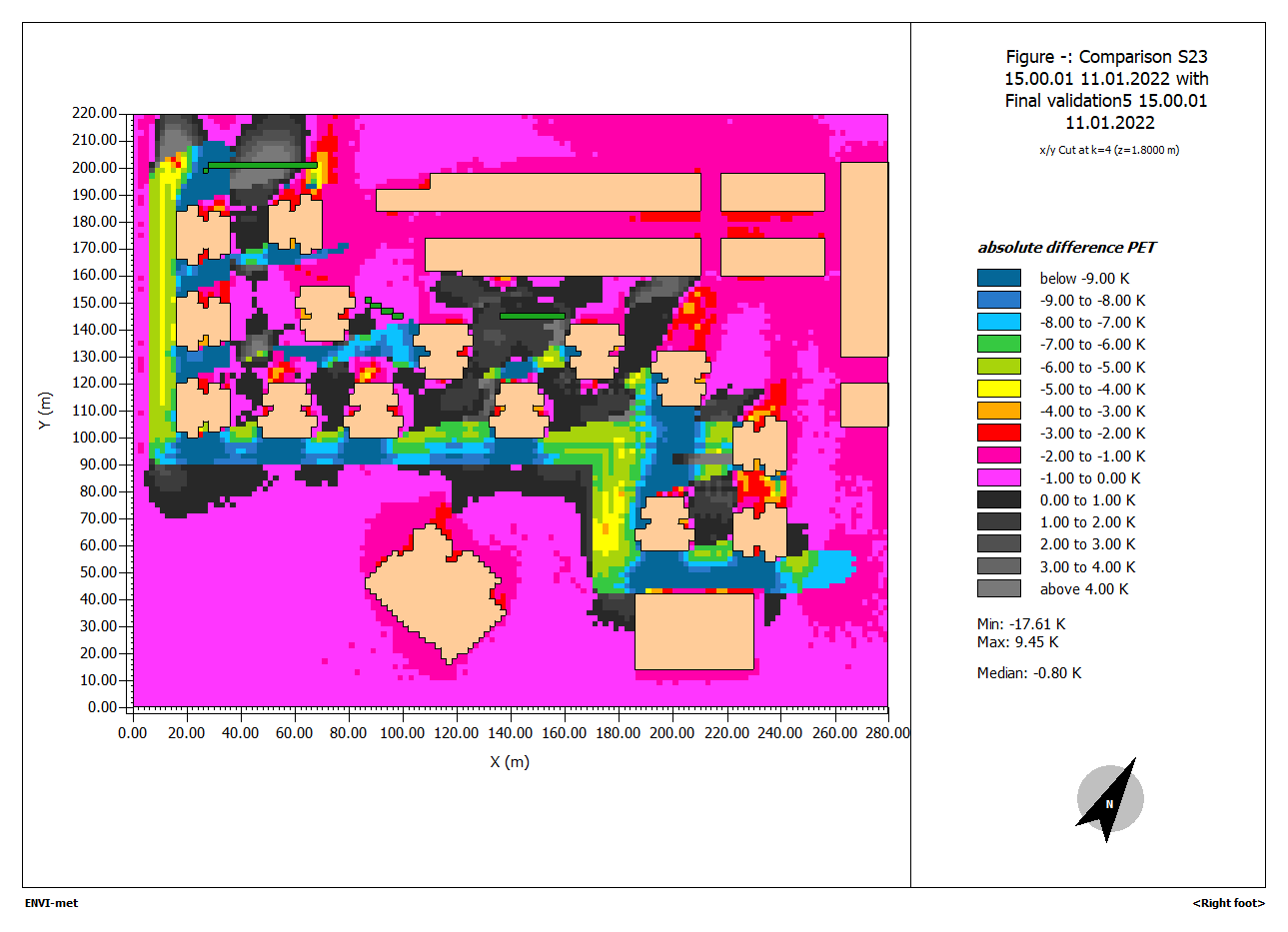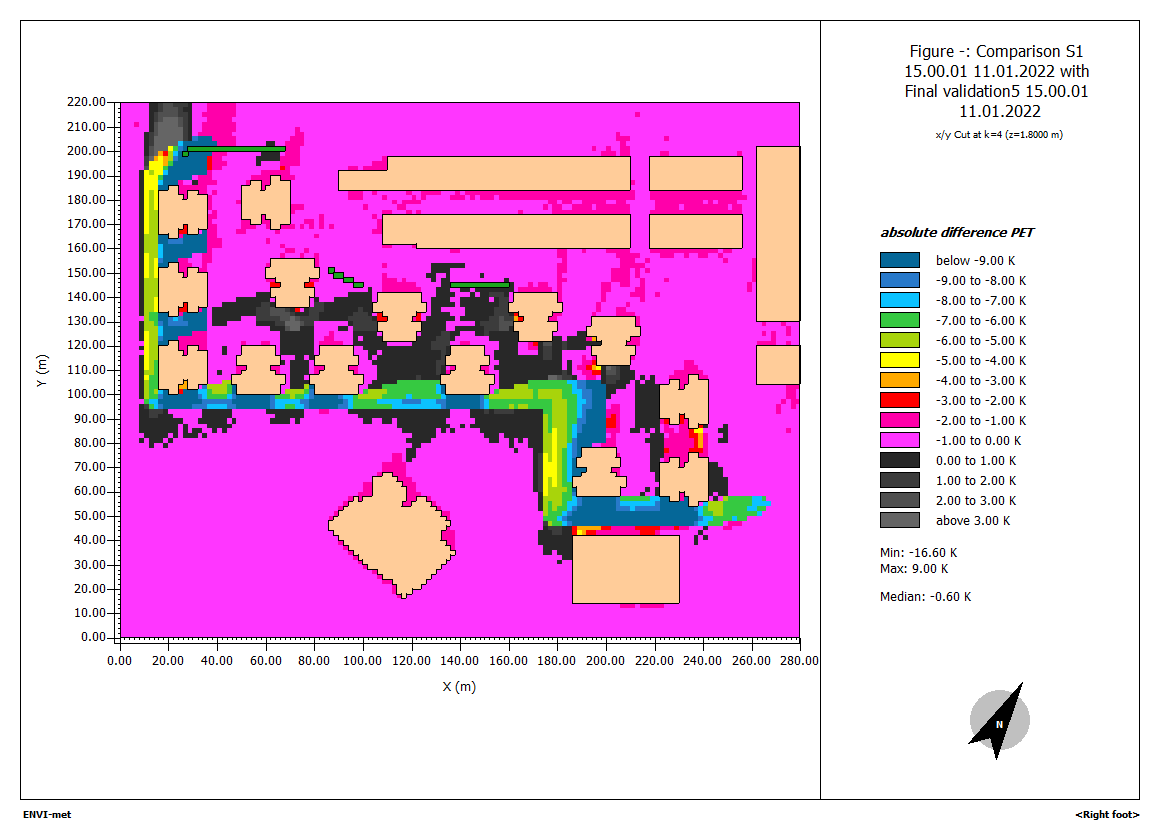 | | | |
| 1. Early morning (7.00) | | | | 1. Noon (15.00) | | | |
| Figure B 21. PET differences between S23 and BCS at 7.00 and 15.00 | | | | | | | |
| 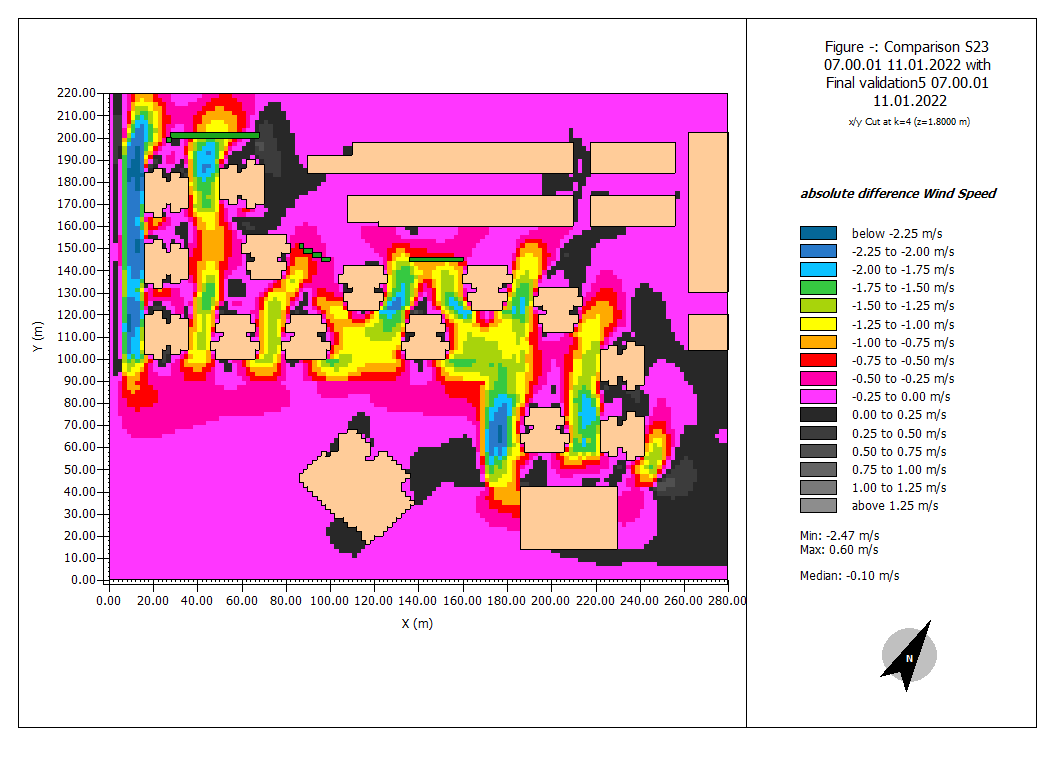 | | 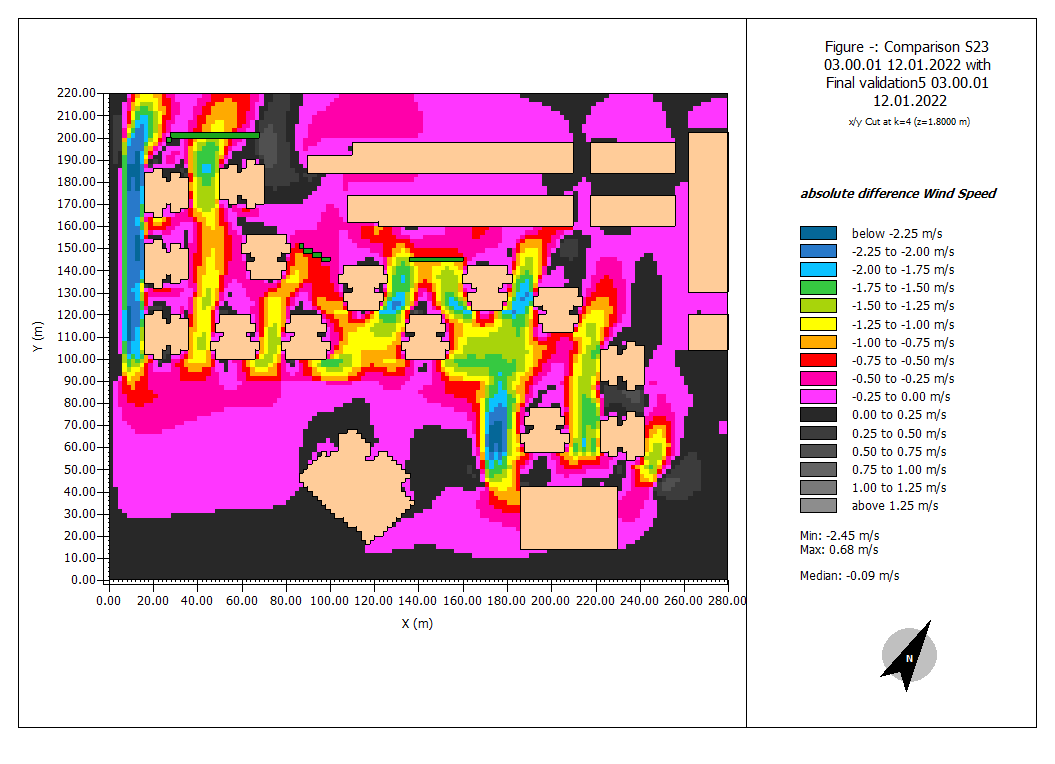 | | | | 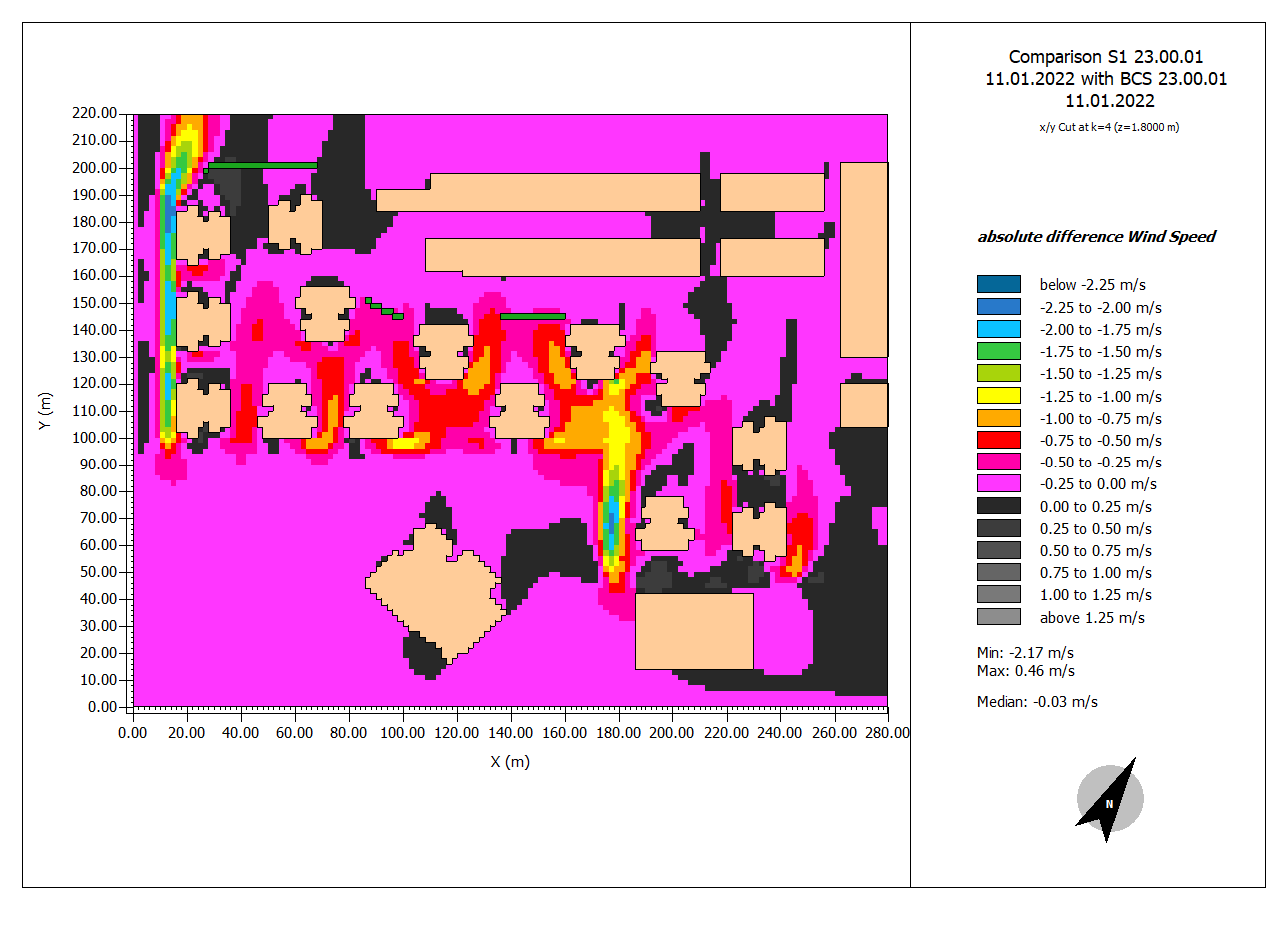 | |
| 1. At 7.00 | | 1. At 23.00 | | | | Scale | |
| Figure B 22. Wind speed differences between S23 and BCS at 7.00 and 23.00 | | | | | | | |
| 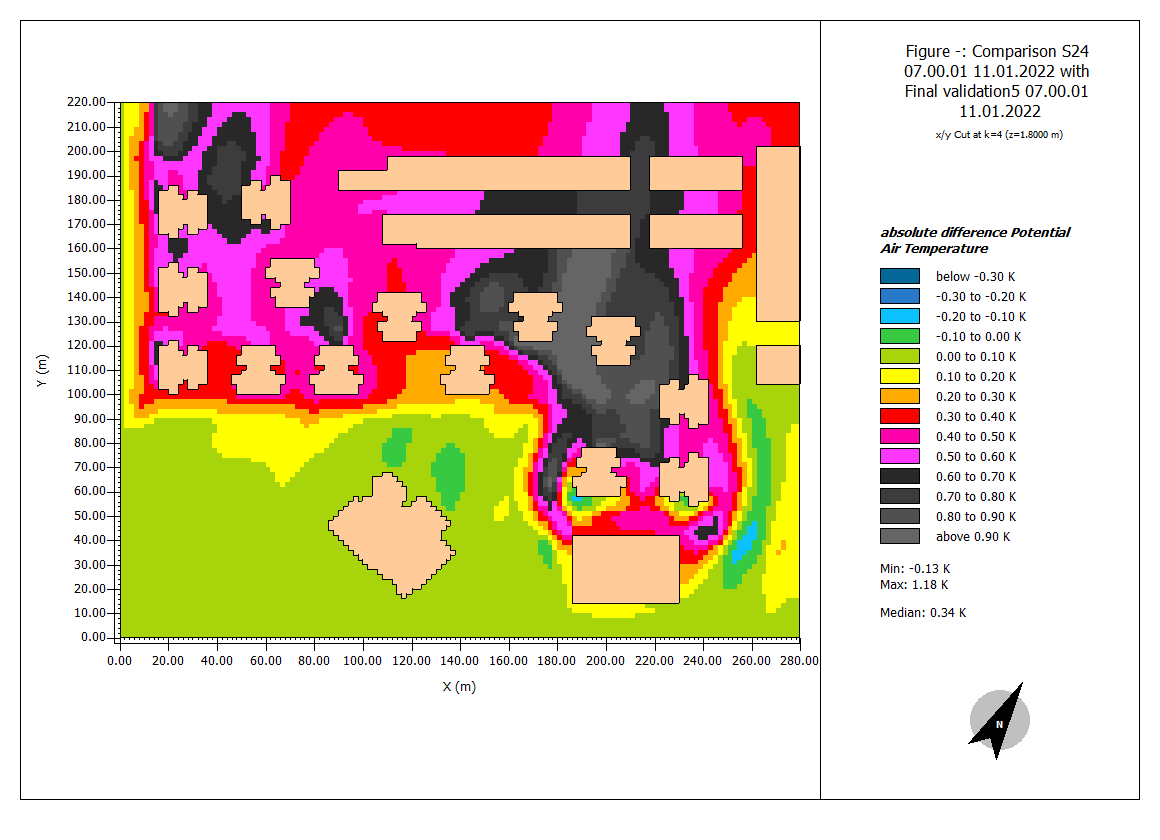 | | | 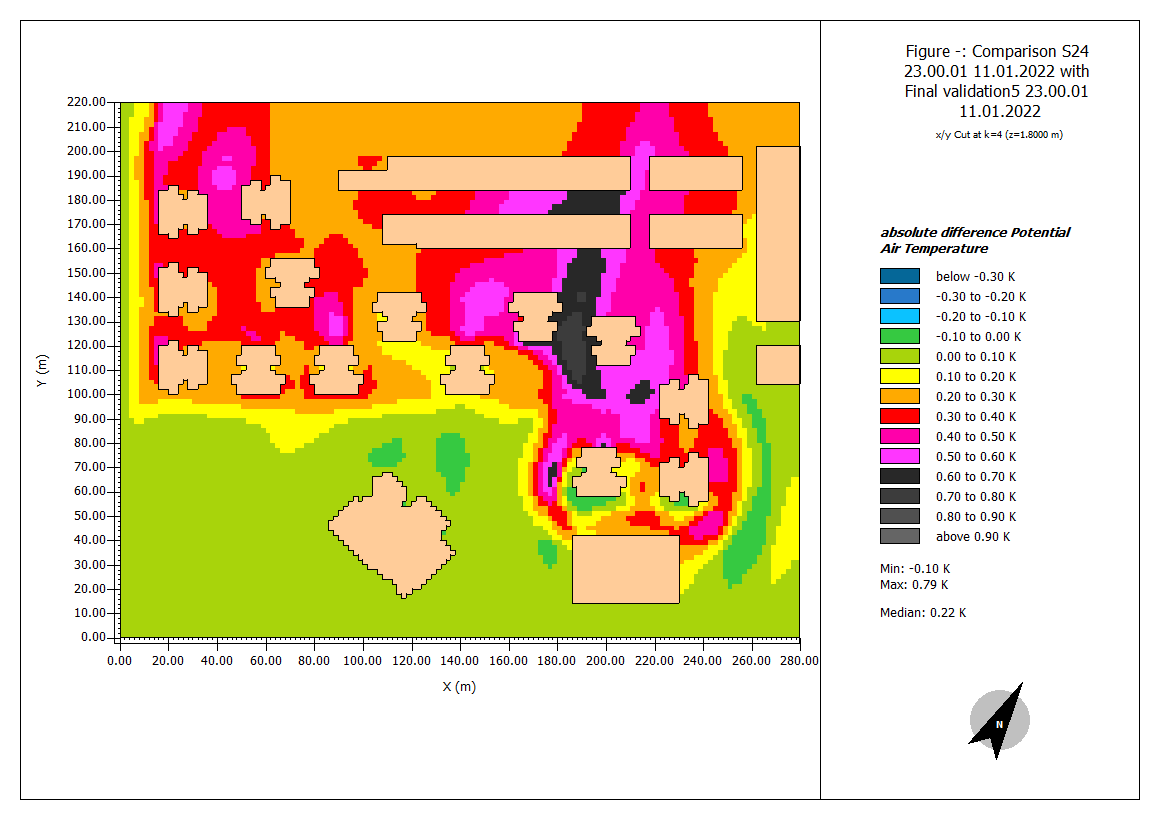 | | | 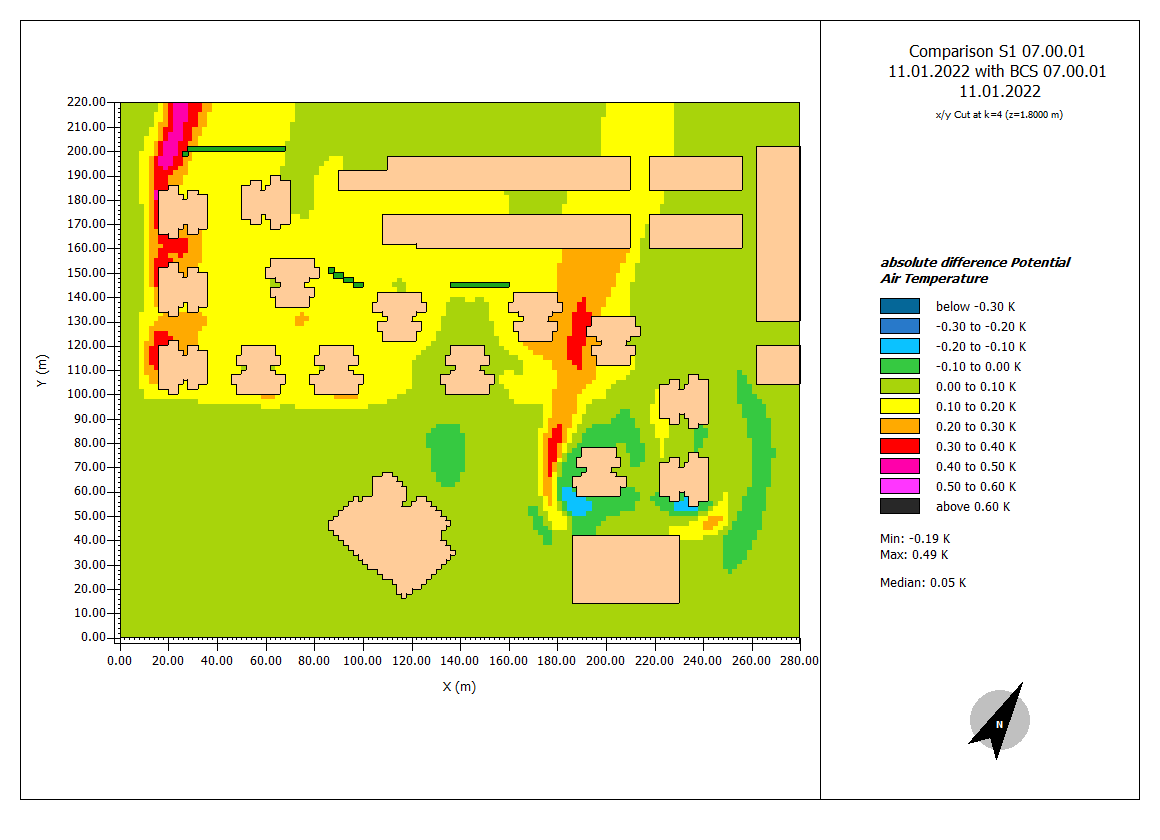 | |
| 1. At 7.00 | | | 1. At 23.00 | | | Scale | |
| Figure B 23. AT differences between S24 and BCS at 7.00 and 23.00 | | | | | | | |
| 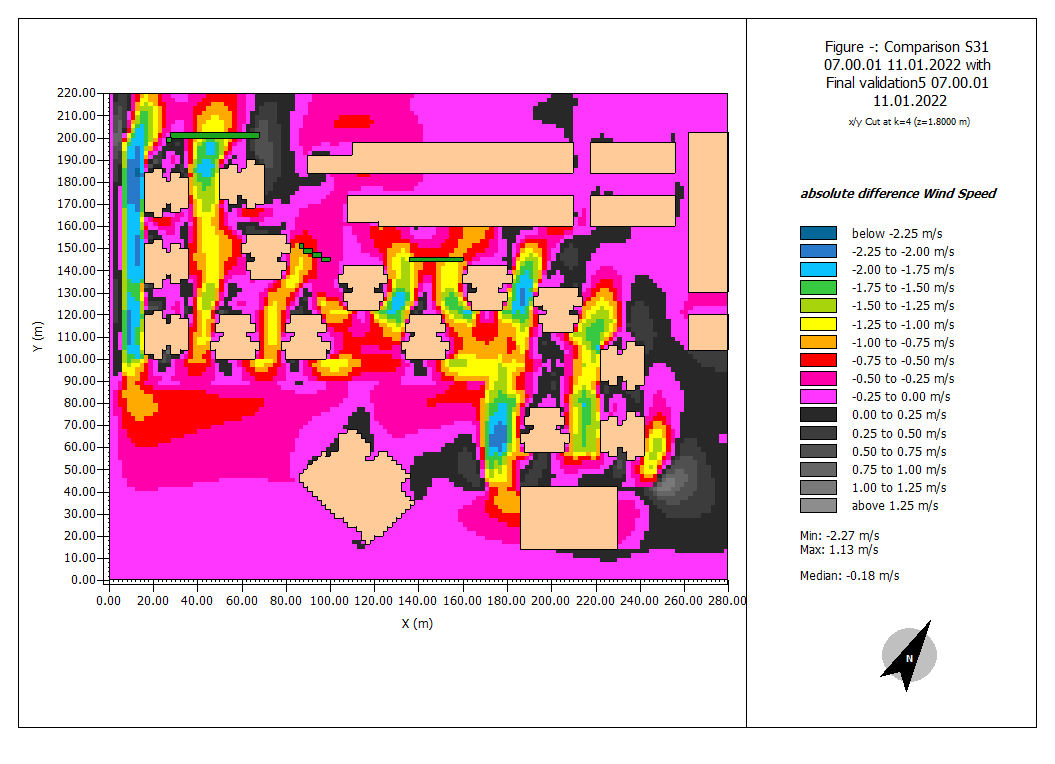 | 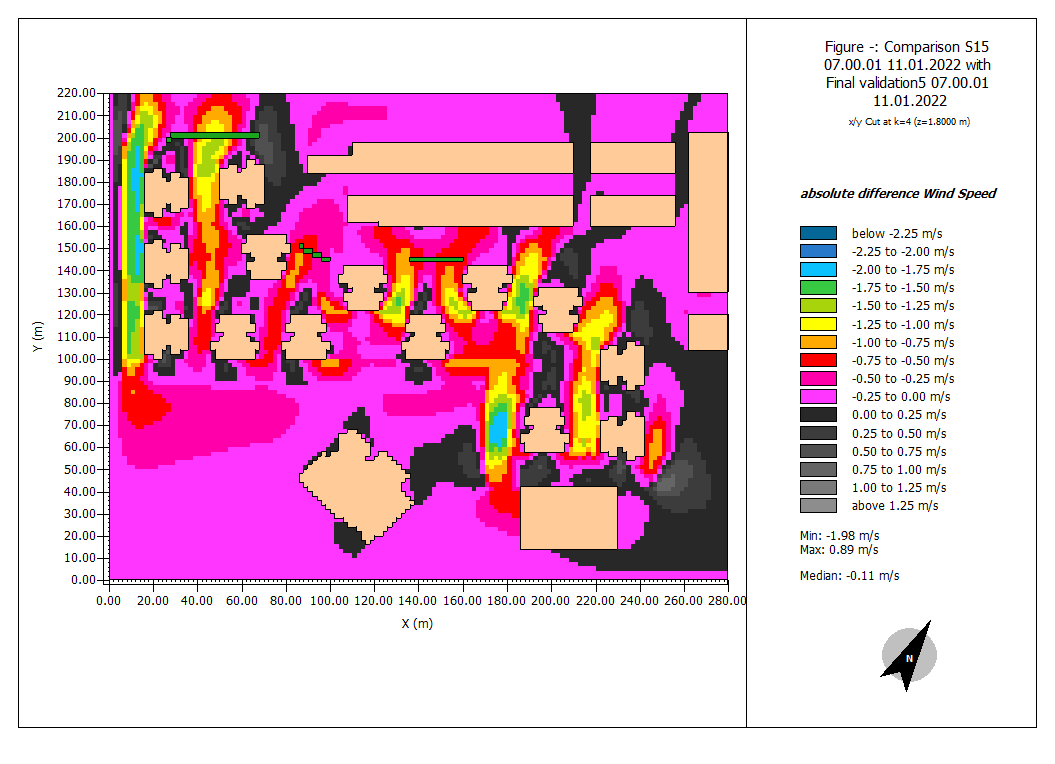 | | | | 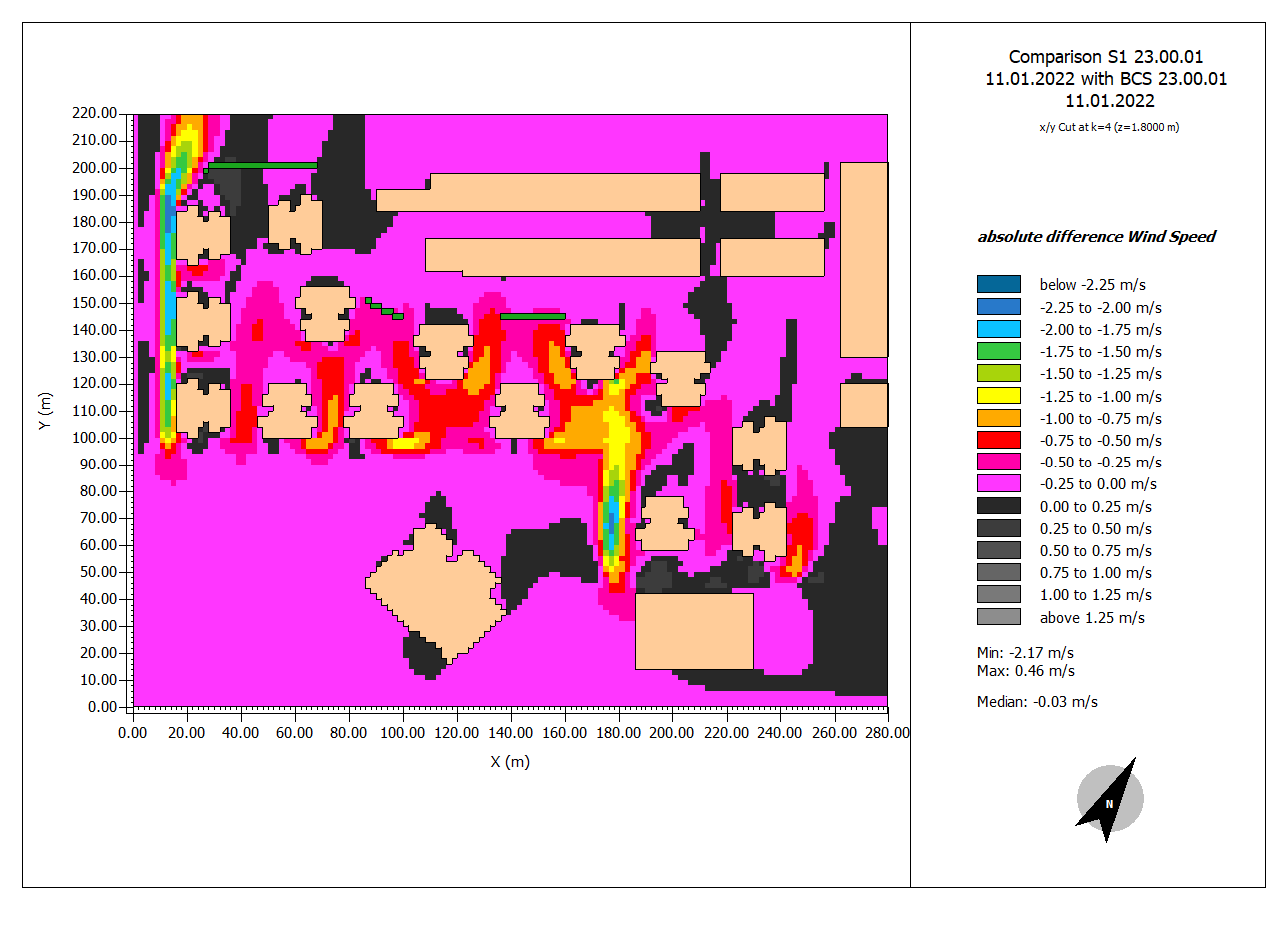 | | |
| 1. S31 (LAD=5) | 1. S15 (LAD=2) | | | | Scale | | |
|  |  | | | |  | | |
| 1. S23 (LAD=5) | 1. S7 (LAD=2) | | | | Scale | | |
| Figure B 24. Wind speed differences between different scenarios and BCS at 7.00. | | | | | | | |
|  |  | | | |  | | |
| 1. S31 (LAD=5) | 1. S15 (LAD=2) | | | | Scale | | |
|  |  | | | |  | | |
| 1. S23 (LAD=5) | 1. S7 (LAD=2) | | | | Scale | | |
| Figure B 25. (AT) at the pedestrian level differences between different scenarios and BCS at 7.00. | | | | | | | |
|  | | | | | | | |
| 1. At 7.00 | | | | | | | |
|  | | | | | | | |
| 1. At 23.00 | | | | | | | |
| Figure B 26. Maximum reduction of cold PET grade area for each parameter at 7.00 and 23.00. | | | | | | | |
|  | | | | | | | |
| Figure B 27. Maximum reduction of comfortable PET grade area improvement for each parameter at 15.00. | | | | | | | |
